# Between-Generation Phenotypic and Epigenetic Stability in a Clonal Snail

**DOI:** 10.1093/gbe/evaa181

**Published:** 2020-09-02

**Authors:** Mark Smithson, Jennifer L M Thorson, Ingrid Sadler-Riggleman, Daniel Beck, Michael K Skinner, Mark Dybdahl

**Affiliations:** School of Biological Sciences, Center for Reproductive Biology, Washington State University

**Keywords:** evolution, epigenetics, DNA methylation, adaptation, transgenerational plasticity

## Abstract

Epigenetic variation might play an important role in generating adaptive phenotypes by underpinning within-generation developmental plasticity, persistent parental effects of the environment (e.g., transgenerational plasticity), or heritable epigenetically based polymorphism. These adaptive mechanisms should be most critical in organisms where genetic sources of variation are limited. Using a clonally reproducing freshwater snail (*Potamopyrgus antipodarum*), we examined the stability of an adaptive phenotype (shell shape) and of DNA methylation between generations. First, we raised three generations of snails adapted to river currents in the lab without current. We showed that habitat-specific adaptive shell shape was relatively stable across three generations but shifted slightly over generations two and three toward a no-current lake phenotype. We also showed that DNA methylation specific to high-current environments was stable across one generation. This study provides the first evidence of stability of DNA methylation patterns across one generation in an asexual animal. Together, our observations are consistent with the hypothesis that adaptive shell shape variation is at least in part determined by transgenerational plasticity, and that DNA methylation provides a potential mechanism for stability of shell shape across one generation.

**Significance**

The role of epigenetic variation in adaptive responses remains controversial, partially because it is difficult to disentangle genetic and epigenetic effects on traits. Clonal organisms that exhibit adaptive phenotypic variation across habitats provide a powerful experimental system for studying the different ways epigenetic variation could affect adaptive phenotypic variation. This study suggests adaptive phenotypic variation in a clonal snail results from a transgenerational response to the environment, which is associated with stability in DNA methylation. This study provides the first evidence of stability of DNA methylation patterns across one generation in an asexual animal. Observations suggest that epigenetic responses to environmental change provide a potential mechanism for the adaptation of asexual animals.

## Introduction

Because the Modern Synthesis, adaptation by natural selection has been thought to rely on random genetic mutations that slowly accumulate over time (Huxley 1942). However, adaptation is often too rapid to be explained by rare genetic mutation, and can occur in populations that lack much genetic variation (Kistner and Dybdahl 2013; Harland et al. 2016; Vásquez et al. 2016). Rapid and adaptive phenotypic responses can also result from within-generation developmental plasticity or transgenerational plasticity mediated by changes in gene expression (Kelly et al. 2012; Mathers et al. 2016). Changes to the epigenome, which can occur more frequently than genetic mutations (Rando and Verstrepen 2007; Becker et al. 2011; Schmitz et al. 2011; Klironomos et al. 2013), provide a potential mechanism for these adaptive phenotypic responses by changing the expression of genes without changing their DNA sequence (Jaenisch and Bird 2003; Dixon et al. 2014). These epigenetic changes, or epimutations, can either occur spontaneously similar to genetic mutations, or they can be induced (Anway et al. 2005; Richards 2006; Verhoeven et al. 2010; Skinner et al. 2013). Epimutations caused by environmental stimuli within generations provide a potential mechanism for developmental phenotypic plasticity. Epimutations caused by ancestral environmental stimuli that are passed on to offspring provide a potential mechanism for persistent parental effects of the environment on phenotypes (Curley et al. 2011; Rando 2012; Kaufmann et al. 2014; McNamara et al. 2014; Alsdurf et al. 2016; Putnam et al. 2016). This could lead to a transgenerational plastic response. Spontaneous epimutations could result in adaptation if they are inherited across multiple generations and respond to selection similarly to genetic mutations (Bossdorf et al. 2008; Skinner 2015). The role of these mechanisms in explaining adaptive phenotypic variation in the absence of genetic variation remains unclear.

**Fig. 1:**
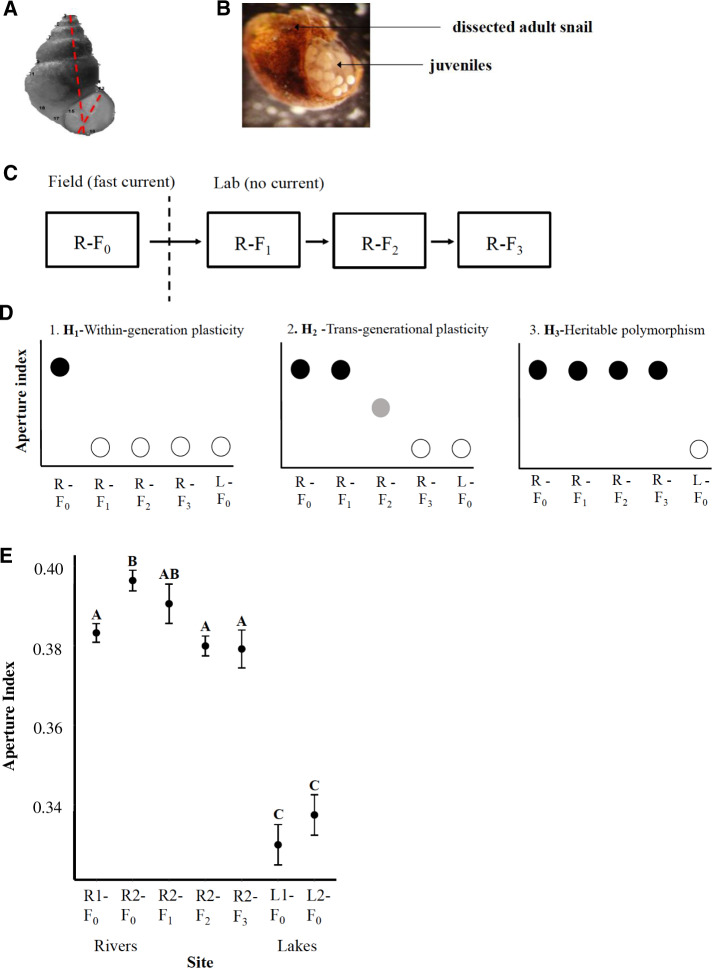
Snail shell shape analysis. (*A*) Shell shape and aperture index. (*B*) Photograph of mature adult snail and juveniles. (*C*) Experimental design and propagation of generations. (*D*) Expected results for phenotypic change over generations in response to the lab environment without current under developmental plasticity, transgenerational plasticity, and genetic/epigenetic inheritance. Black shows aperture index characteristic of the river environment. White shows aperture index characteristic of the lake environment. Gray shows an intermediate aperture index. (*E*) Mean Aperture index of all seven populations. Aperture index for four populations from the field, and three lab-reared generations. R1-F0 and R2-F0 show the aperture index for two populations collected from the river sites, R2-F1 generation, R2-F2 generation, and R2-F3 generation show the aperture index for three lab-reared generations from the R2 population, and L1 (Lake Lytle) and L2 (Lake Washington) represent the two lake populations. Significantly different means from a Tukey’s post hoc test on an ANOVA of aperture index across all populations are indicated by different letters. Error bars indicate one standard error from the mean.

A recent study identified adaptive differences in shell morphology and differential DNA methylation between populations of a single clonal genotype of the New Zealand freshwater snail inhabiting lakes and rivers in the western United States. Specifically, snails in river environments have wider apertures for their height (greater aperture index), allowing for larger foot muscles to grip substrates in environments with high-current velocity (Kistner and Dybdahl 2014; Thorson et al. 2017). Two lines of evidence suggest that this variation in shell shapes within a clonal lineage is adaptive. First, the shell shape variation that is observed between lakes and rivers (Thorson et al. 2017) matches adaptive morphological variation observed in other snail species (Vermeij 1980,^ 1995^). Second, in a comparison of shell shape variation between *Potamopyrgus antipodarum* and a native snail species across sites with different current speed, the same morphological differences were observed. This finding was consistent with an adaptive hypothesis (Kistner and Dybdahl 2014). The adaptive variation in shell shape in river environments could result from developmental plasticity, persistent maternal effects of the environment, or divergence in genetic or epigenetic polymorphisms resulting from selection for beneficial variants that are heritable across generations. In the event these phenotypic differences represent developmental plasticity and respond to the environment, they should disappear after one generation when exposed to a new environment. Alternatively, should these differences remain after the first generation in the new environment *then shift in subsequent generations*, they would represent transgenerational plasticity. Finally, if the differences remain unchanged across multiple generations, they then represent heritable epigenetic or genetic divergence (fig. 1*D*). These alternative scenarios are by no means mutually exclusive.

It appears less likely that phenotypic divergence in a relatively young invasive population of a single *P. antipodarum* clonal lineage will involve selection based on variation at a genetic level. Previous analyses of genetic differences (including microsatellites, allozymes and mtDNA) could not detect any genetic variation among study populations (Dybdahl and Drown 2011). Consequently, we expected adaptive plasticity to explain divergence, and asked whether epigenetic variation (DNA methylation) was associated with habitat and phenotypic divergence. Epigenetics has been defined as “molecular factors and processes around DNA that regulate genome activity independent of DNA sequence, and are mitotically stable,” which includes DNA methylation, histone alterations, noncoding RNA, chromatin structure, and RNA methylation (Nilsson et al. 2018). Interestingly, we identified more differential methylated DNA regions (DMR) between the lake and river habitats than between replicate sites within the same environment (Thorson et al. 2017), suggesting a role for DNA methylation in phenotypic divergence. These differences in DNA methylation could be responsive to a new environment and disappear after one generation, or they could persist relatively unchanged in a new environment, suggesting transgenerational plasticity, or epigenetic polymorphisms.

This study was designed to investigate the stability of phenotypic and epigenetic variation when exposed to a new environment. The offspring of snails from a natural river habitat with higher current speed than lake habitats were raised for three generations in a controlled laboratory environment with no water current. To determine whether the adaptive phenotypic variation results from developmental plasticity, transgenerational plasticity, or heritable genetic or epigenetic polymorphism, we compared shell aperture index of the wild river population to that of the lab generations. To determine whether DNA methylation is stable between generations, we compared patterns of DNA methylation among these populations and the F1 generation offspring using methylated DNA immunoprecipitation (MeDIP). Our observations suggest that shell shape of river snails was unchanged in the first lab generation and changed gradually afterward with some proportion of the variation stable across three generations. Furthermore, the stability of shell shape in the first generations was associated with stable transmission of epigenetic marks for one generation.

## Results

### Shell Shape Analysis

To test whether the adaptive variation in shell shape in the fast-current river site results from within-generation plasticity, transgenerational plasticity, or heritable variation, the reproductive snails from the River 2 (fast current) site were cultured in a controlled environmental chamber with no water current at Washington State University, as described in the Materials and Methods section. The breeding strategy with five different iso-female lines and sample sizes for each generation are summarized in supplementary figure S1, Supplementary Material online. All five F0 females contributed offspring to the F1 generation. Three of the five females contributed to the F2 and F3 generation offspring. The first, second, and third generation descendants of these reproductive females were propagated and cultured in the lab without water current, figure 1*B* and *C*. In the event the River 2 first generation of lab snails (R2-F1) shifts to the shape of snails found in lakes, the adaptive status of shell shape likely results from a within generation response to the new current-speed environment, figure 1*D*. In the event the first generation of lab snails (R2-F1) retains the shell shape found in rivers and the second and third generations (R2-F2 and R2-F3) shift toward the lake shell shape, the adaptive differences in shell shape likely are a result of a “transgenerational plasticity” response, figure 1*D*. In the event the shell shape of the first, second, and third generation lab snails (R2-F1, R2-F2, and R2-F3) remains similar to that of river (R-F0) snails, the adaptive differences in shell shape likely result from a heritable polymorphism (fig. 1*D*).

Shell shape differences between populations inhabiting different current environments can be summarized by comparing aperture index (aperture width/shell height, fig. 1*A*) of snails from two rivers (R1-F0 and R2-F0) and two lakes (L1-F0 and L2-F0). In Thorson et al. (2017) (22), it was shown that aperture index significantly differed among populations (one way ANOVA, *F* = 42.6, *P* = 2e−16). In this study, it was also found that aperture index significantly differed between the slow- and fast-current river sites (*P* = 0.0415) (fig. 1*E* and supplementary table S1, Supplementary Material online). Consistent with adaptation to a fast-current environment the aperture index of snails collected from the fast-current river site was significantly larger than that of snails collected from slow-current river site (fig. 1*E*).

To test whether this variation in aperture index among snails collected from different currents was stable or responsive to the environment, we compared the aperture index of fast-current river snails cultured in a zero-current lab environment to that of wild-caught river and lake snails. The aperture index of first lab-reared generation (R2-F1) did not significantly differ from its parental generation (R2-F0). However, the aperture index of the subsequent generations (R2-F2 and R2-F3) was significantly smaller (fig. 1*E* and supplementary table S1, Supplementary Material online). Furthermore, the aperture index of the second and third lab-reared generations (R2-F2 and R2-F3) did not significantly differ from the wild-caught river snails from the R1 site (R1-F0) (fig. 1*E* and supplementary table S1, Supplementary Material online). In addition, the aperture index of the first, second, and third generation lab-reared river snails (R2-F1, R2-F2, and R2-F3) significantly differed from both lake populations (L1-F0 and L2-F0) (supplementary table S1, Supplementary Material online). Although the second and third generation snails aperture index decreased toward the aperture index of wild caught lake snails, they were still more similar to the river than lake populations. There was no significant difference in shell shape between the F1, F2, and F3 generations. These observations suggest no significant within-generation developmental plasticity, and a transgenerational shift in aperture index in generations two and three toward a smaller aperture index in response to the zero-current lab environment. Differences between third-generation and lake snails suggest that at least some of the shell shape variation between lake and river habitats is stable across three generations.

To check the possibility that the shift in shell shape was the result of the change in maternal lineages represented across generations, we analyzed shell shape variation for only the lineages (2, 3, and 5) that were represented across the F1, F2, and F3 generations (supplementary fig. S2, Supplementary Material online). Although this analysis lacks power to isolate the effects of maternal lineage on shell shape, it tests whether underrepresentation of lineages 1 and 4 in the F2 and F3 generations contributed to the differences in shell shape across generations. When lineages 1 and 4 were completely excluded from our analysis, the same relative differences in shell shape detected before (fig. 1) were still observed among the F0, F1, F2, and F3 generations (supplementary fig. S3, Supplementary Material online). Therefore, we conclude that the underrepresentation of lineages 1 and 4 across the F1, F2, and F3 generations did not account for the observed shift in shell shape.

### DNA Methylation Analysis

DNA methylation of the wild-caught populations and the F1 generation of lab reared snails (two lakes, two rivers, and the F1 from R2) were analyzed using three pools of 10–20 individuals from each population. Sixty snails from each wild-caught river population were sequenced in three pools, with 20 individuals in each pool. Thirty snails from each wild-caught lake population were sequenced in three pools, 10 individuals in each pool. Thirty R2-F1 snails from each wild-caught lake population were sequenced in three pools, with 10 individuals in each pool. The methylated DNA was isolated for each pool using MeDIP of the fragmented (300–500 bp) genomic DNA. MeDIP DNA pools were used to create sequencing libraries, using a MeDIP-Seq procedure previously described (Thorson et al. 2017; Skinner et al. 2018). The pools were sequenced on an Illumina HiSeq 2500 sequencing platform, which yielded approximately 60 million reads per pool. As a reference genome, an expression cDNA library previously described was used (26).

**Fig. 2:**
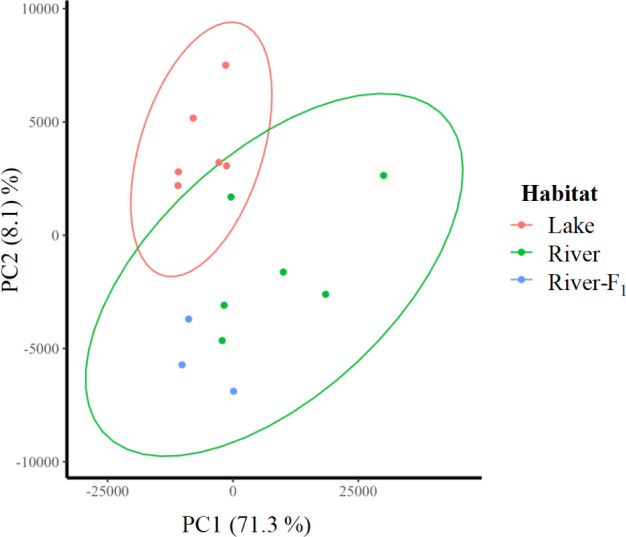
PCA based on normalized read counts (RPKM) of DNA methylation for all genomic windows. Three DNA pools are plotted for each population. Lake and river populations separate along PC2. The F1 generation population clusters with the river populations on PC2 suggesting stability of DNA methylation of genomic windows that load on that axis.

To test the stability of epigenetic variation specific to the high-current river site, we conducted three different analyses. First, we conducted a principal components analysis (PCA) on the read depths of 739,509 windows (100 bp in size) across the genome. In a plot of the first and second principal components, the river populations clustered together, as did the lake populations. If methylation patterns were stable, we would expect the three pools of the R2-F1 population to cluster closer to the two river populations than to the two lake populations, which they did (fig. 2 and Supplementary fig. S4, Supplementary Material online).

Next, we conducted two analyses based on differentially methylated regions (DMR). DMR were identified for single 100 bp sites (windows). We also checked for how many 100 bp windows the DMR extended in each direction. Because we explored DMR using a range of *P* value thresholds, we included DMR identified at each threshold in supplementary table S2, Supplementary Material online. The DMR for each comparison are presented in supplementary tables S3–S7, Supplementary Material online with all the genomic characteristics, statistics, and associated genes listed. A summary of DMR lengths and locations in CpG deserts or islands is provided in the methods section.

**Fig. 3:**
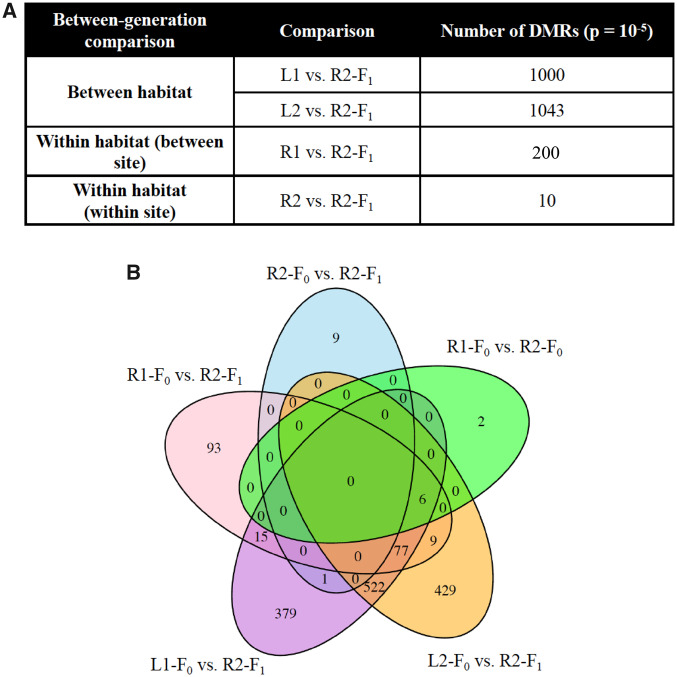
(*A*). Between-generation DMR (between habitat, between site, and within site). (*B*) Overlap of between-generation DMR.

If methylation patterns are stable between generations, we expected fewer DMR between R2-F1 and river populations compared with the number of DMR between R2-F1 and lake populations. In fact, we identified almost an order of magnitude fewer DMR between the R2-F1 population and both river populations than between the R2-F1 population and both lake populations (*P* value threshold of *P* < 1e−05, fig. 3*A*). These DMR all had an FDR adjusted *P* value of less than 0.01. We also expected fewer DMR between generations from the same population (R2-F0 and R2-F1) compared with the number of DMR between generations from the other river population (R2-F1 and the R1-F0). We again identified an order of magnitude fewer DMR between the R2-F1 population and the R2-F0 population than between the R2-F1 population and the R1_F0 population at the same threshold (*P* value threshold of *P* < 1e−05, fig. 3*A*). The DMR in these two comparisons all had an FDR adjusted *P* value less than 0.1.

If these stably inherited DMR were associated with lake and river differences, then we expected a large number of the same DMR (overlap) in comparisons of R2-F1 and each lake (L1 and L2). Indeed, DMR identified between R2-F1 and both lakes showed a lot of overlap (fig. 3*B*). To extend the overlap analysis, less stringent statistical comparisons were made using the *P* < 1e−05 DMR set with the others at *P* < 0.05. Most notably, this analysis showed 98–99% overlap between the DMR sets comparing the F1 river generation and each of the two lakes (R2-F1 vs L1 and R2-F1 vs L2) (supplementary fig. S5*A*, Supplementary Material online). This observation suggests that methylation patterns of the R2-F1 differ from both lakes in a similar way.

**Fig. 4:**
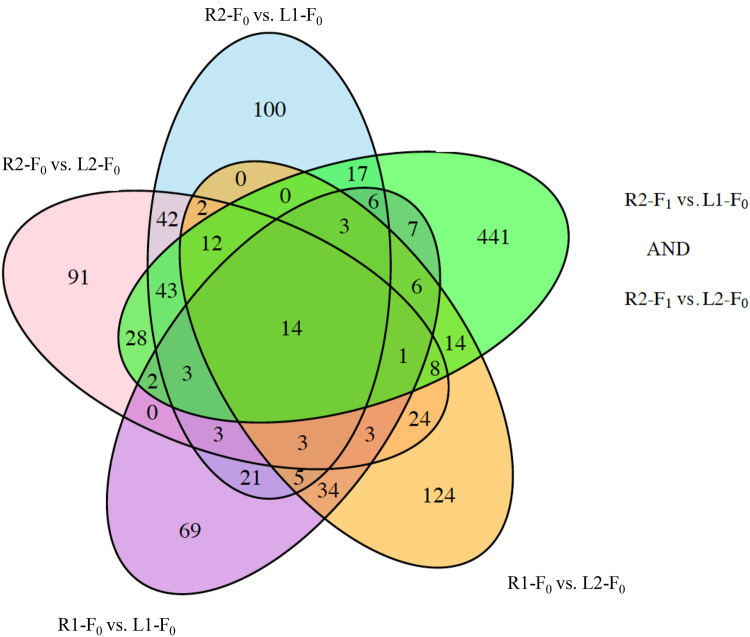
Proportion of site-specific DMR that are stable across one generation. (*A*) Overlap of DMR between R2-F0 and Lakes. (*B*) Overlap of DMR between R2-F1 and Lakes. (*C*) Site-specific DMR that are stable across at least one generation.

If DNA methylation specific to river habitats was stable, we expected the DMR between wild-caught lake and river populations to overlap with DMR between wild-caught lake populations and the lab reared R2-F1 population. First, we identified overlapping DMR in the R2-F1 versus L1 and R2-F1 versus L2 comparisons. We found 605 DMR in this overlap (Green oval in fig. 4). Next we asked how many of these overlapping DMR between the R2-F1 and lakes (Green oval) are also identified in lake versus river comparisons (Parts of green oval that overlaps with other ovals in fig. 4). Of the 605 DMR, 164 were also identified in one of the lake versus river comparisons. These represent putatively stable DMR between lake and river sites. In the most conservative analysis, we asked how many of the overlapping 605 DMR in the R2-F1 versus lake comparisons were present in all 4 lake versus river comparisons. Although a smaller subset, 14 DMR showed this pattern of habitat-specific stability.

Functional analyses of DMR between the R2-F1 population and the field populations are summarized in supplementary figure S5*B*, Supplementary Material online. No functional relationship between differentially methylated genes and variation in shell shape was identified. The majority of DMR between the R2-F1 population and the lake populations were near genes related to metabolism. For a similar analysis of DMR between lakes and rivers, see Thorson et al. (2017). Investigating functions of genes near DMR provides some value in investigating correlations between DNA methylation and trait variation. However, it is important to realize that methylation can regulate distant genes whose function cannot be identified. It is also important to note is that using a transcriptome as a reference restricted our analysis to methylation of transcribed genes. This approach focuses on gene body methylation, which is thought to play an important role in gene regulation for invertebrates (Roberts and Gavery 2012). However, it does not provide information on promoter methylation.

## Discussion

The importance of epigenetic variation in evolutionary change and adaptation depends on the stability of epigenetic variation and its effect on phenotypic variation. We were interested in the determinants of adaptive shell shape variation among populations of an invasive clonal snail. The stability of phenotypic and epigenetic variation between populations from river and lake environments was investigated in shell aperture dimensions and DNA methylation patterns (Thorson et al. 2017). We found that phenotypic variation was unchanged for one generation and changed slowly in subsequent generations in a new environment. We also found that epigenetic variation was stable for the first generation.

When snails from a fast-current river environment were cultured in a lab environment without current for three generations, the mean aperture index of the first generation population did not differ significantly from the mean aperture index of their mothers from the river population, but did in subsequent generations (fig. 1 and supplementary table S1, Supplementary Material online). Stability of environmentally specific shell morphology for one generation in a divergent lab environment suggests that adaptive shell morphology in the fast-current river population was not solely the result of within-generation developmental plasticity. In response to the zero-current lab environment, the second and third generations developed shell morphology that was slightly closer to the lake populations than was the F1-generation river population. These observations are consistent with transgenerational plasticity (fig. 1*D*), where the traits of offspring were determined by parental environments. Overall, the shift in shell shape appears to be affected to some extent by transgenerational plasticity, although a role for developmental plasticity cannot be entirely ruled out. Because after three generations in a lab environment without current, the mean aperture index of snails propagated from a river population was still significantly different from both lake populations (supplementary table S1, Supplementary Material online), heritable genetic and/or epigenetic polymorphism may also contribute to shell shape variation.

It is possible that shifts in shell shapes observed in generations two and three could have been caused by an environmental variable, other than current flow, that differed between lab and field populations (e.g., temperature, diet). Future studies can test this by comparing shell shapes of snails from lake and river populations across generations in both slow- and fast-current environments. This test would also be important, because it is possible that responses of shell shape of snails going from no current to high current might be more rapid. The speed and magnitude of plastic responses might depend on the direction of environmental change. If we had the technology to create a river current in the lab environment, a powerful design would have been to split broods from each isolated mother into fast and slow-current speed.

We cannot rule out the hypothesis that shell shape adaptation to lake and river environments resulted from genetic variation. However, for a few reasons, a nongenetic explanation may be more likely. First, genetic variation would have required rapid accumulation of beneficial mutations affecting shell shape because the colonization of its invasive range in the western United States in 1987. Yet, allozymes, mtDNA, and microsatellites all failed to detect genetic variation within this clonal lineage throughout the invaded range (Dybdahl and Drown 2011). Second, if genetic variation is lacking, then divergent selection would have had to be strong to produce such rapid phenotypic divergence between all lake and river populations in this study. Finally, if the adaptive variation in shell morphology was completely genetically encoded, we would not have expected any response in shell shape across the three generations in the zero-current lab conditions. Because the lack of recombination in asexual reproduction (Fisher 1930) prevents combination of mutations from different iso-female lines, mutations affecting shell morphology would have had to occur independently in multiple lines. It is possible that the plasticity in shell shape results from an adaptive genome by environment interaction. For example, a previous study showed parallel adaptive shell shape variation between *P. antipodarum*, a recent invader, and *Pyrgulopsis robusta*, a native snail (Kistner and Dybdahl 2014). We hypothesize that the adaptive plasticity involved in the response of the invasive and native snails was present (and likely genetically encoded) in the common ancestor of this species and other mollusks. Although beyond the scope of this study, future work will use higher resolution methods to measure genetic variation.

Based on the expected dearth of genetic variation within and among recently founded invasive populations, we were curious about the role of nongenetic mechanisms that might lead to more rapid phenotypic divergence. One such mechanism is epigenetic modifications, such as DNA methylation, which occurs orders of magnitude more rapidly than genetic mutations (Rando and Verstrepen 2007; Becker et al. 2011; Schmitz et al. 2011). There are two main ways in which DNA methylation variation could account for adaptive phenotypic variation between environments (Platt et al. 2015). First, it is possible that epimutations are reliably inherited and respond to selection, forming stable epigenetic polymorphisms that have accumulated as a result of environmental selection. Inheritance of DNA methylation through the germ line has been shown in other animals (Reik and Surani 2015), and inheritance of DNA methylation might be more likely in asexual organisms like *P. antipodarum*, which bypass critical steps in de-methylation during meiosis (Verhoeven and Preite 2014). Theory suggests that the faster rate of epimutations causes them to respond to natural selection before genetic mutations (Klironomos et al. 2013), and might play a critical role in local adaptation to peripheral habitats (Smithson et al. 2019). If these selectively favored epimutations determined shell shape, we might have expected no change over three generations of lab rearing, similar to our predictions about genetic variation. Given the gradual change in shell shape across three generations (R2-F0 through R2-F3, fig. 1*E*), adaptation through selection on genetic variation seems unlikely.

Second, it is possible that DNA methylation changes were induced directly by lake and river environments. Environmentally triggered methylation changes could account for developmental plasticity and the appearance of adaptive shell morphology within a generation. However, in our experiment, shell shape did not respond to environmental conditions within a generation, arguing against developmental plasticity as the sole source of shell shape variation. However, we recognize that it is possible, but not likely, that F1 generation eggs might have received environmental signals whereas brooded by F0 mothers that were living in river currents, resulting in a within-generation plasticity by “developmental conversion” during very early development in utero. However, the vast majority of development time and exposure to the current environment for the F1 generation occurs after the 2- and 3-week brooding period. Nevertheless, we cannot rule out that the shell shape of the F1 generation was influenced by the F0 mother’s environment (within generation plasticity), although we would argue that the influence is likely relatively small.

Alternatively, environmentally triggered epimutations could account for transgenerational plasticity in shell shape if they are partially stable. Stability of these environmentally induced changes across one generation could explain the stability of shell morphology across one generation. The transgenerational dynamics of shell shape observed in this study are consistent with an “epigenetic washout,” where F0 environment completely determines the trait expression of the F1 generation, and the effect of the F0 environment on F2 and F3 generations is gradually degraded [see fig. 1*A* from {31}].

Given the stability of shell shapes across one generation, we might have expected DNA methylation to be stable across the first generation if it contributes to shell shape variation. In fact, multiple analyses of DNA methylation data suggest that DNA methylation specific to the river habitat was stable across one generation. Genome-wide DNA methylation levels across all genomic windows (i.e., DMR and non-DMR) showed that the F1 population clustered closer to river than to lake populations (fig. 2). The lowest number of DMR of all comparisons was detected between the F1 population and their F0 parental population. The FDR adjusted *P* value also shows 0 DMR at *P* < 0.05. Finally, DMR identified between wild-caught lake and river snails overlapped with DMR identified between F1 and the wild-caught lake snails. Like the observed stability in shell shape variation, we cannot completely rule out that genetic variation underlies DNA methylation variation, whether related to shell shape variation or not. However, one advantage to studying DNA methylation variation in asexuals is that the contribution of genetic variation to trait variation is more restricted. The results are equally consistent with environmentally induced DNA methylation that is stable across one generation. Stability of environment-specific DNA methylation across one generation has been reported in asexual plants (Verhoeven et al. 2010), yet our study may provide the first evidence of stability of DNA methylation patterns across one generation in an asexual animal. This finding supports the hypothesis that critical steps in demethylation are bypassed during asexual reproduction (Verhoeven and Preite 2014).

The stability of both shell morphology and DNA methylation across one generation is consistent with a role of epigenetic variation in the rapid and adaptive phenotypic divergence between lakes and rivers, but by no means causal evidence. Testing causality was beyond the scope of this study. The stability of shell morphology rejects the hypothesis that the phenotypic variation results from developmental plasticity, and the slight shift in phenotype observed in generations two and three matches a pattern of transgenerational plasticity via epigenetic washout. However, an appreciable portion of the variation in aperture index between invasive populations may result from heritable polymorphism, because the phenotype of generations two and three was still closer to that of rivers than lake snails. Future analyses of DNA methylation in generations two and three would further test the contribution of changes in DNA methylation to the shift in shell shape. Epigenetic mechanisms of transgenerational plasticity might increase phenotypic variation, persistence and fitness of asexual populations, which do not generate genetic variation through sexual recombination. The faster route to adaptive trait variation provided by epigenetic mechanisms of transgenerational plasticity could alter the fate of populations during range expansions, invasions, and rapidly changing climates.

## Conclusion

Habitat-specific adaptive shell shape variation in *P. antipodarum* is stable across one generation. Therefore it seems unlikely that adaptive shell shape variation results solely from developmental plasticity. Habitat-specific DNA methylation was stable for one generation, and provides a potential mechanism for the phenotypic stability. Future studies are now needed to determine if the DNA methylation changes observed are stable in subsequent generations. An adaptive, yet incomplete, shift in shell shapes relative to the wild-caught F0 generation occurred in generations two and three, which suggests that adaptive phenotypic variation between environments might result from a transgenerational response to local environments. Overall, our findings suggest that adaptive phenotypic variation in clonal animals might arise from a transgenerational response to the environment, and that epigenetics (e.g., DNA methylation) provides a potential mechanism.

## Materials and Methods

### Sample Collection

A previous study examined shell shape and methylation from two lake and two river sites (Thorson et al. 2017). Lake snails were collected from Lake Lytle in Rockaway Beach, OR (referred to as L1: 45.6272°N, 123.9392°W), and a beach area of Lake Washington in Seattle, WA (referred to as L2: 47.6971°N, 122.2711°W). River snails were collected from tributary spring stream of the Snake River at Ritter Island (referred to as R2: 42.7439°N, 114.8420°W), and the main channel of the river (referred to as R1: 42.7439°N, 114.8416°W) near Wendell, ID. Sixty snails were collected from each of the river sites and 30 snails were collected from each of the lake sites. For the transgenerational observations in this study, we collected reproductive snails from the R2 site. This site was chosen to maximize the difference in current from the laboratory environment where no current is present. Along with having the highest and most consistent current rate of the sites from Thorson et al. (2017), the population of snails at this site also had the highest mean aperture index, which is consistent with morphological adaptation to high-current rates among gastropods (Vermeij 1980, 1995). Samples were obtained by searching the substrate and scraping snails off the underside of rocks and woody debris. The samples were maintained on wet paper towels and kept cool until they reached the laboratory at Washington State University, Pullman, WA.

### Iso-Female Lines and Shell Shape Analysis

The F1 generation snails experienced a river environment as embryos and shelled juveniles within F0 mothers. These early F1 stages are brooded in the mantle cavity for approximately 2 and 3 weeks, so this represents the maximum time of exposure within the F0 mother that was living in the river environment. As soon as shelled juveniles leave the brood chambers, they were isolated in a zero-current environment.

For the transgenerational observations of shell shape, five iso-female lines were initiated with snails collected from the R2 site. Snails were isolated in plastic cups to start iso-female lines. All lab snails were maintained at a temperature of 14 °C, and a salinity of 2–4 ppt on 12:12 light-dark cycles. Water was changed and snails were fed 2–3 times a week*.* Offspring of the five females were isolated into smaller plastic cups where they were raised until they were approximately 1 mm in length. When they reached this size, they were transferred to larger cups. After the first generation reproduced, snails were photographed under a dissecting microscope and their tissue was harvested for analysis of DNA methylation. The second and third generations were raised under the same conditions and shell shapes were measured after females were reproductive. A summary of when offspring were isolated and harvested across the three generations is included in supplementary figure S1, Supplementary Material online. Supplementary figure S2, Supplementary Material online shows the sample sizes of each iso-female line across the three generations. Thirty snails from R2-F1 were sampled from the 32 total offspring that were reared to maximize evenness in the contribution of each iso-female line and make three pools of 10 individuals in the methylation analysis. Specifically, two snails from the maternal lineage with the most offspring (lineage 2) were excluded from analysis of both shell shape and DNA methylation in the F1 generation to maximize evenness in the number of snails analyzed for each lineage. Shell height and aperture width was measured with ImageJ. The aperture index (aperture width/shell height) of individual snails from the river and lake population was determined.

### Tissue

For the transgenerational study of DNA methylation, we dissected a homogenous population of cells from a thin slice of foot tissue of R2-F1 generation snails, as in Thorson et al. (2017). This tissue was used to control for tissue based differences in DNA methylation. Tissue from 10 individuals was pooled (3 pools total/population) and DNA was extracted from these pools. Foot tissue, and the phenotype measured in this study, aperture index, is likely correlated traits because the aperture is the opening where the snail foot protrudes. Both traits are known to affect performance in fast currents. The foot tissue serves as a marker for environmental exposure consistently from one generation to the next, including those inherited from the germ line and those resulting from exposure to the current speed environment in a tissue related to performance.

### Genomic DNA Preparation

Genomic DNA isolation from the field populations is described in Thorson et al (2017). Genomic DNA from the F1 lab-reared population was isolated at a later date, due to time needed to rear the F1 generation, and with the following protocol. Genomic DNA from snail foot tissue of the F1 laboratory population was sonicated then suspended in 100 μl of 1 × Phosphate Buffered Saline (PBS), then 820 μl DNA extraction buffer (50 mM Tris pH8, 10 mM EDTA pH8, 0.5% SDS) and 80 μl Proteinase K (20 mg/ml) was added and the sample incubated on a rotator at 55 °C for 2–3 h. After incubation, 300 μl of protein precipitation solution (Promega, A795A) was added, the sample was mixed and incubated on ice for 15 min, then it was spun at 4 °C at 13,000 rpm for 20 min. The supernatant was transferred to a fresh tube, then precipitated over night with the same volume 100% isopropanol and 2 μl glycoblue at −20 °C. The sample was then centrifuged and the pellet was washed with 75% ethanol, then air-dried, and resuspended in 100 μl H_2_O. DNA concentration was measured using the Nanodrop (Thermo Fisher).

### Methylated DNA Immunoprecipitation

MeDIP from genomic DNA was performed to quantify levels of methylation. Approximately 6 μg of each genomic DNA pool was diluted to 130 μl with TE buffer into the appropriate Covaris tube. Covaris was set to 300–500 bp program. A volume of 10 μl of each sonicated DNA was run on 1.5% agarose gel to verify fragment size. The sonicated DNA was transferred from the Covaris tube to a 1.7 ml microfuge tube, and the volume was measured. The sonicated DNA was then diluted with TE buffer (10 mM Tris HCl, pH7.5; 1 mM EDTA) to 400 μl, heat-denatured for 10 min at 95 °C, then immediately cooled on ice for 5 min. Then 100 μl of 5× IP buffer and 5 μg of antibody (monoclonal mouse anti 5-methyl cytidine; Diagenode no. C15200006) were added to the denatured sonicated DNA. The DNA–antibody mixture was incubated overnight on a rotator at 4 °C.

The following day, magnetic beads (Dynabeads M-280 Sheep anti-Mouse IgG; 11201D) were prewashed as follows: the beads were resuspended in the vial, then the appropriate volume (50 μl per sample) was transferred to a microfuge tube. The same volume of Washing Buffer (at least 1 ml) was added and the bead sample was resuspended. The tube was then placed onto a magnetic rack for 1–2 min, and the supernatant was discarded. The tube was removed from the magnetic rack and the washed beads were resuspended in the same volume of 1× IP buffer as the initial volume of beads. A 50 μl of beads were added to the 500 μl of DNA–antibody mixture from the overnight incubation, then incubated for 2 h on a rotator at 4 °C.

After the incubation, the beads were washed three times with 1× IP buffer as follows: the tube was placed into a magnetic rack for 1–2 min, and the supernatant was discarded, then washed with 1× IP buffer 3 times. The washed beads were then resuspended in 250 μl digestion buffer (50 mM Tris-HCI pH8, 10 mM EDTA pH8, 0.5% SDS) and with 3.5 μl Proteinase K (20 mg/ml). The sample was then incubated for 2–3 h on a rotator at 55 °C. Following incubation, the tube was put again back into the magnetic rack for 3 min, and the supernatant was removed to a new microfuge tube. The beads were discarded. A 250 μl of buffered phenol–chloroform–isoamyl alcohol solution were added to the supernatant and the tubes were vortexed for 30 s, then centrifuged at 14,000 rpm for 5 min at room temperature. The aqueous supernatant was carefully removed and transferred to a fresh microfuge tube. Then 250 μl of chloroform were added to the supernatant from the previous step, vortexed for 30 s, and centrifuged at 14,000 rpm for 5 min at room temperature. The aqueous supernatant was removed and transferred to a fresh microfuge tube; 2 μl of glycoblue (20 mg/ml), 20 μl of 5 M NaCl and 500 μl ethanol were added to the supernatant and mixed well, then precipitated in a −20 °C freezer overnight.

The precipitate was centrifuged at 14,000 rpm for 20 min at 4 °C and the supernatant was removed while not disturbing the pellet. The pellet was washed with 500 μl cold 70% ethanol in −20 °C freezer for 15 min, then centrifuged again at 14,000 rpm for 5 min at 4 °C and the supernatant was discarded. The tube was spun again briefly to collect residual ethanol, and then as much liquid as possible was removed with a gel loading tip. The remaining pellet was air-dried at RT until it looked dry (about 5 min), then resuspended in 25 μl H_2_O or TE. DNA concentration was measured in Qubit with ssDNA kit.

### Bioinformatics and Statistics

DMR are identified based on differences in the number of sequenced methylated fragments (i.e., reads) that align to that region of the genome. For the DMR analyses, the basic read quality was verified using summaries produced by the FastQC program http://www.bioinformatics.babraham.ac.uk/projects/fastqc/. The raw reads were trimmed and filtered using Trimmomatic (version 0.36) with a minimum phred score of 33 and the minimum length of reads to keep set at 20. The reads for each MeDIP sample were mapped to the *P. antipodarum* (Wilton et al. 2013) partial genome using Bowtie2 (Bolger et al. 2014) with default parameter options. The mapped read files were then converted to sorted BAM files using SAMtools (Langmead and Salzberg 2012). To identify DMR, the reference genome was broken into 100 bp windows. The MEDIPS and edgeR (Robinson et al. 2010) R packages were used to calculate differential coverage between control and exposure sample groups. The edgeR *P* value was used to determine the relative difference between the two groups for each genomic window. Windows with an edgeR *P* value less than 10^-5^ were considered DMR. The DMR edges were extended until no genomic window with a *P* value <0.1 remained within 1,000 bp of the DMR. CpG density and other information were then calculated for the DMR based on the reference genome. DMR were annotated using BLAST results for each contig (Wilton et al. 2013). The associated genes were then sorted into functional groups by consulting information provided by the DAVID (Huang et al. 2009) and Panther (Mi et al. 2013) databases incorporated into an internal curated database (www.skinner.wsu.edu under genomic data). All molecular data have been deposited into the public database at NCBI (GEO nos GSE93836, GSE133502, and pending).

The majority of differentially methylated DNA regions were single windows (i.e., 100 bp in length), but some DMR extended across multiple 100 bp windows. Analysis of the genomic features of the DMR demonstrated lengths of 200–600 bp, Supplementary figure S4, Supplementary Material online. The differentially methylated DNA regions were located in low density CpG deserts with <10 CpG/100 bp and 1–8 CpG/100 bp being predominant (Supplementary fig. S5, Supplementary Material online). DMR were identified in all comparisons (fig. 3). The DMR for each comparison are presented in supplementary tables S3–S8, Supplementary Material online with all the genomic characteristics, statistics, and associated genes listed. The DMR-associated gene functional categories are also presented and a summary of the DMR-associated gene categories is presented in Supplementary figure S3, Supplementary Material online. The predominant gene categories were metabolism and signaling.

## Supplementary Material

evaa181_Supplementary_Data
