# Supplementary material for: Between-Generation Phenotypic and Epigenetic Stability in a Clonal Snail
Source: Genome Biol Evol. 2020 Sep 2;12(9):1604–15. doi: 10.1093/gbe/evaa181 (PMC7513791; doi:10.1093/gbe/evaa181)
Supplement: evaa181_Supplementary_Data [file evaa181_supplementary_data.pdf]

Supplemental Material Captions

Supplemental Figure S1. Timeline of when the F1, F2, and F3 generations were isolated and harvested.

Supplemental Figure S2. Sample sizes of maternal lineages 1-5 in the F1, F2, and F3 generations.

Supplemental Figure S3. Mean aperture index of snails from the R2-F0 field population compared to the mean aperture index of snails in maternal lineages 2,3, and 5 from the R2-F1, R2-F2, and R2-F3 populations. Error bars indicate standard error. Aperture index of snails significantly differed among generations (ANOVA, Tukey's post-hoc). Significantly different groups are indicated by the bold letters.

Supplemental Figure S4. PCA based on normalized read counts (RPKM) of DNA methylation for all genomic windows. Habitats are denoted by colors and sites are denoted by shape.

Supplemental Figure S5. (A) Comparison of each DMR comparison from Figure 3 at $p<1e-05$ with others at $p<0.05$. The overlapped DMR number is presented and corresponding percentage (%). **(B)** Gene functional categories associated with DMRs in each comparison (in Figure 3).

Supplemental Figure S6. The DMR lengths. All DMRs at a p-value threshold of $p<1e-05$ are shown here. **(A)** R1 (slow) versus R2 (fast). **(B)** R2-F1 versus R1 **(C)** R2-F1 versus R2 **(D)** R2-F1 versus L1 (Lytle). **(E)** R2-F1 versus L2 (Washington).

Supplemental Table S1. Results of the Tukey's post hoc test from an ANOVA of aperture index across all seven populations. P-values adjusted for multiple comparisons using Tukey's HSD (p adj.) are reported and significant comparisons are denoted with an asterisk. All possible pairwise comparisons between populations are separated into five categories.

Supplemental Table S2. DNA methylation analysis. The number of DMRs found using different p-value cutoff thresholds. The All Window column shows all DMRs. The Multiple Window column shows the number of DMRs containing at least two significant windows. **(A)** R1 versus R2. **(B)** R2-F1 generation versus R1 **(C)** R2-F1 generation versus R2 **(D)** R2-F1 generation versus L1 (Lytle). **(E)** R2-F1 generation versus Lake 2 (Washington).

Supplemental Table S3. DMRs identified between R1 and R2 with genomic characteristics, statistics, and associated genes.

Supplemental Table S4. DMRs identified between R2 and R2-F_1 with genomic characteristics, statistics, and associated genes.

Supplemental Table S5. DMRs identified between L1 and R2-F_1 with genomic characteristics, statistics, and associated genes.

Supplemental Table S6. DMRs identified between R1 and R2-F_1 with genomic characteristics, statistics, and associated genes.

Supplemental Table S7. DMRs identified between L2 and R2-F_1 with genomic characteristics, statistics, and associated genes.

Supplementary Figures and Tables

Supplemental Figure S1

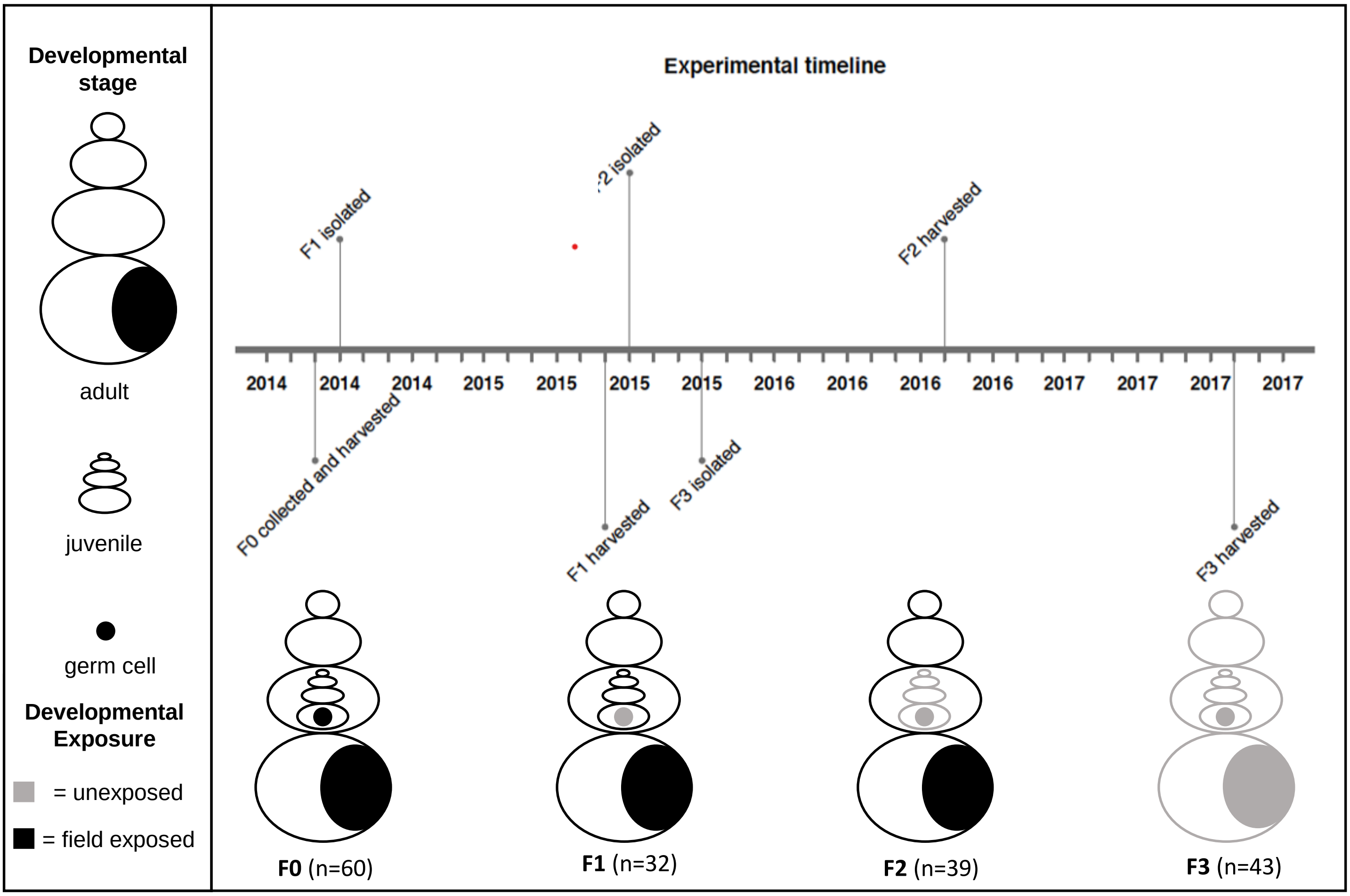

Supplemental Figure S2

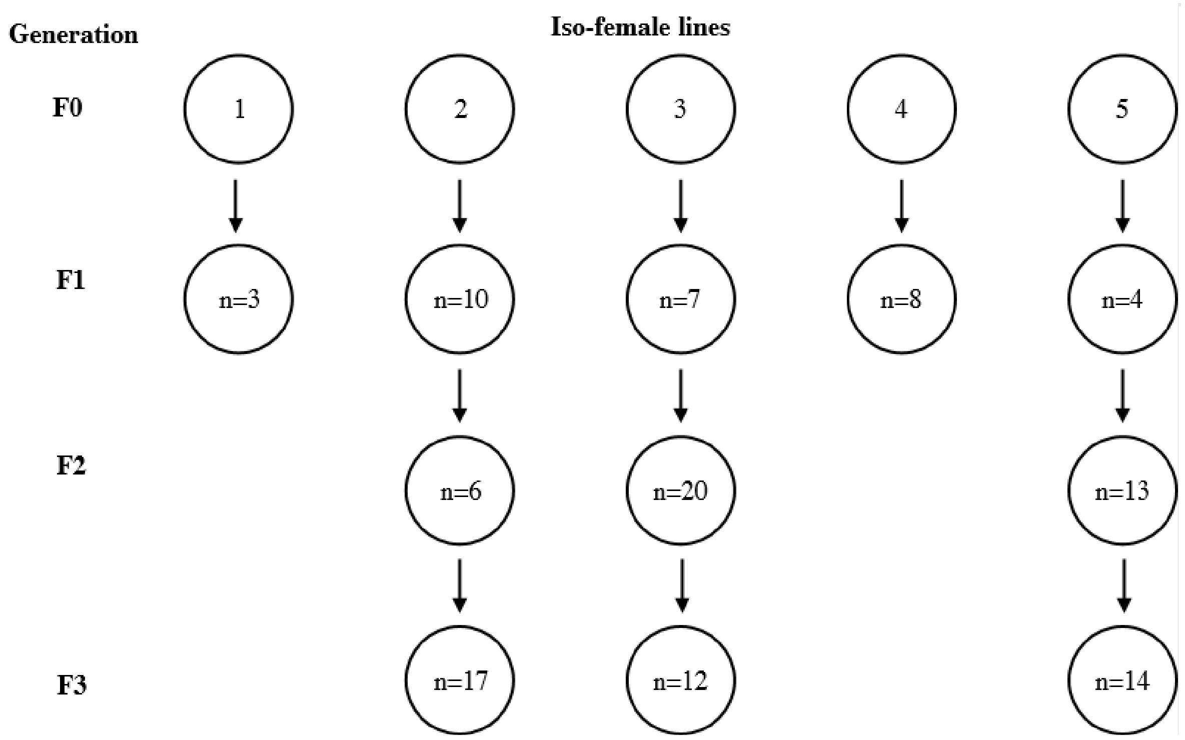

Supplemental Figure S3

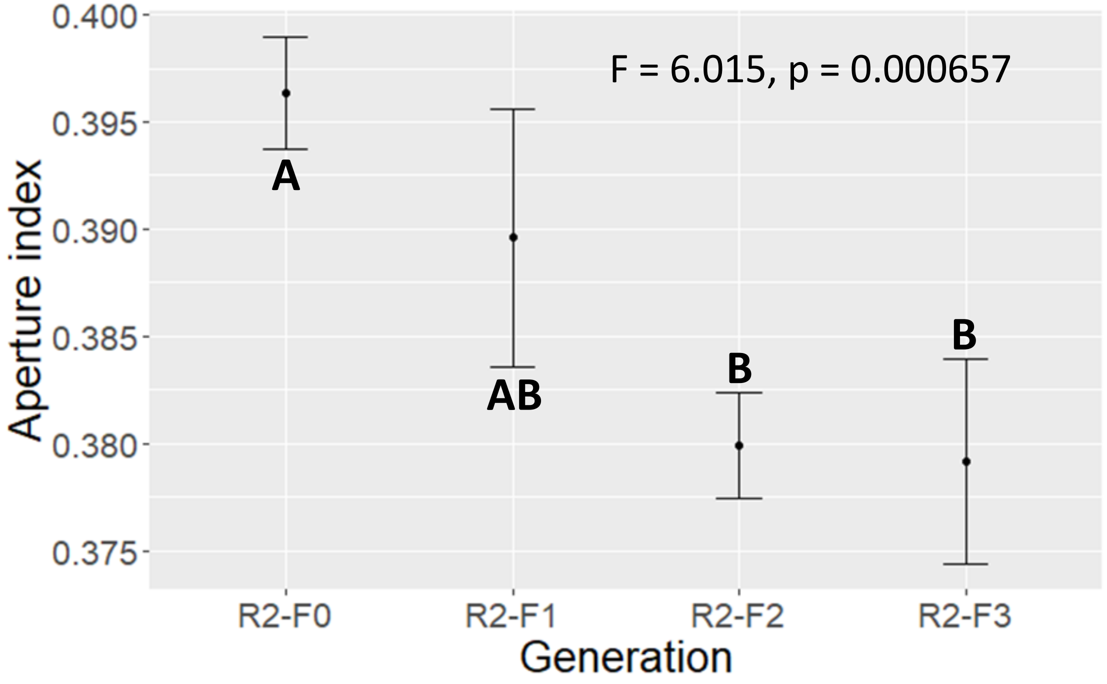

Supplemental Figure S4

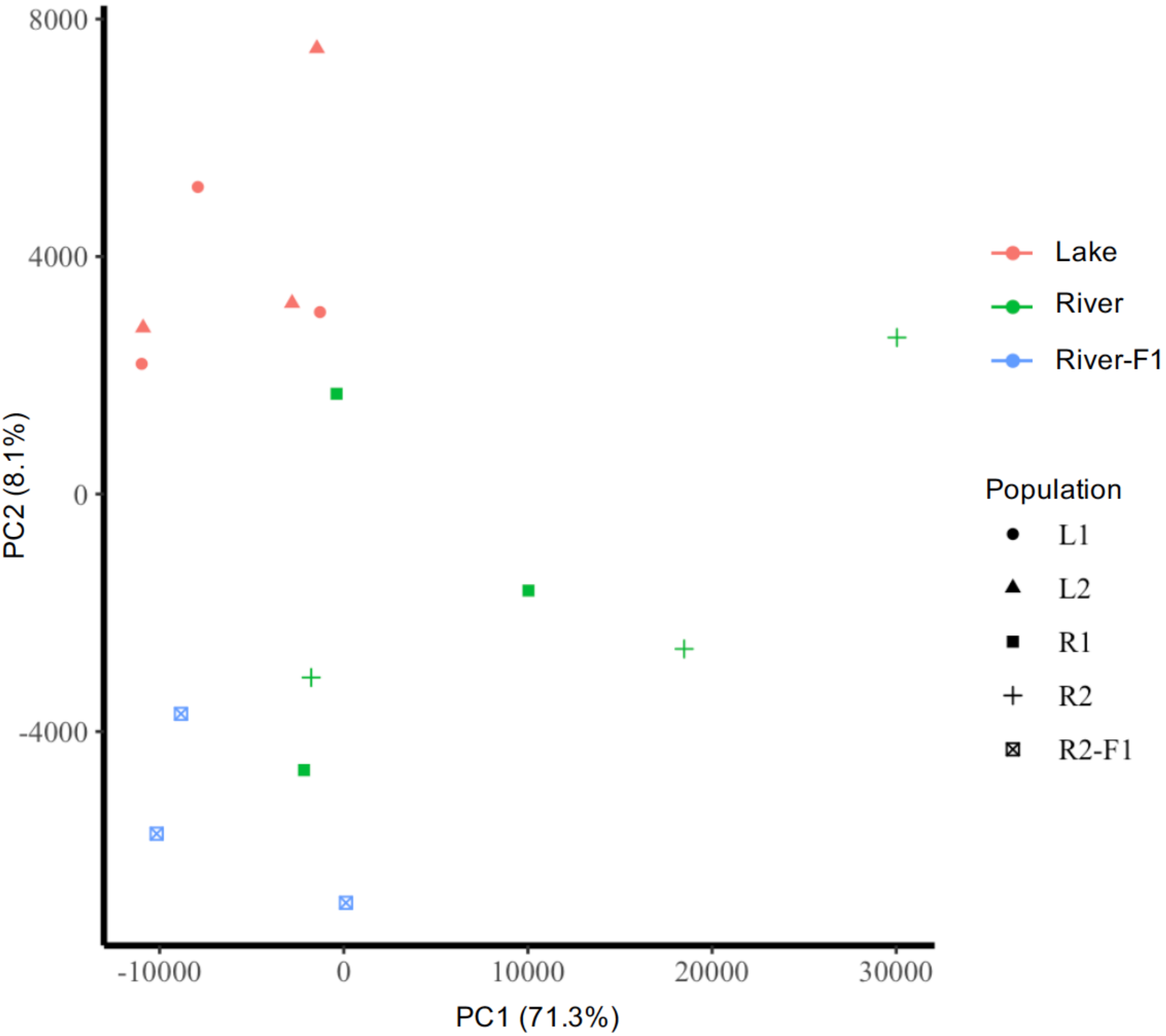

Supplemental Figure S5

A DMR Overlaps

p<1e-05 \ p<0.05	R2-F1 vs L1	R2-F1 vs L2	R2-F1 vs R1	R2-F1 vs R2
R2-F1 vs L1	1000 (100%)	986 (99%)	681 (68%)	363 (36%)
R2-F1 vs L2	1027 (98%)	1043 (100%)	728 (70%)	379 (36%)
R2-F1 vs R1	189 (94%)	184 (92%)	200 (100%)	154 (77%)
R2-F1 vs R2	5 (50%)	7 (70%)	7 (70%)	10 (100%)

B DMR Associated Gene Categories

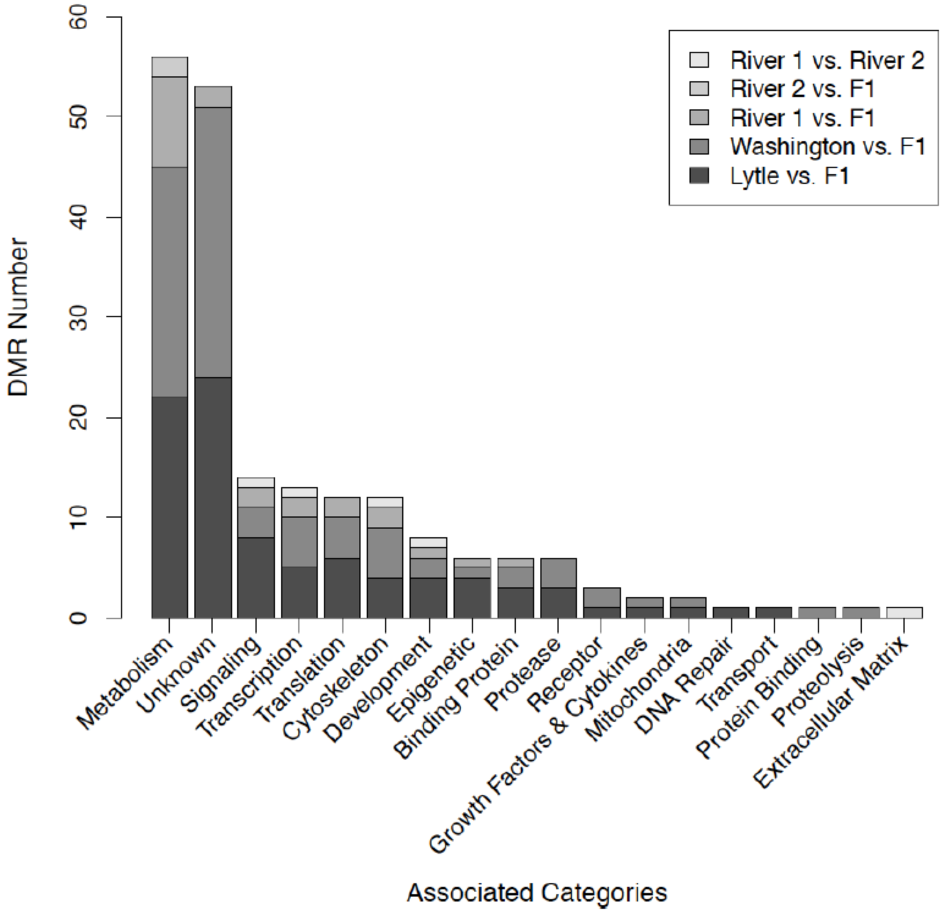

Supplemental Figure S6

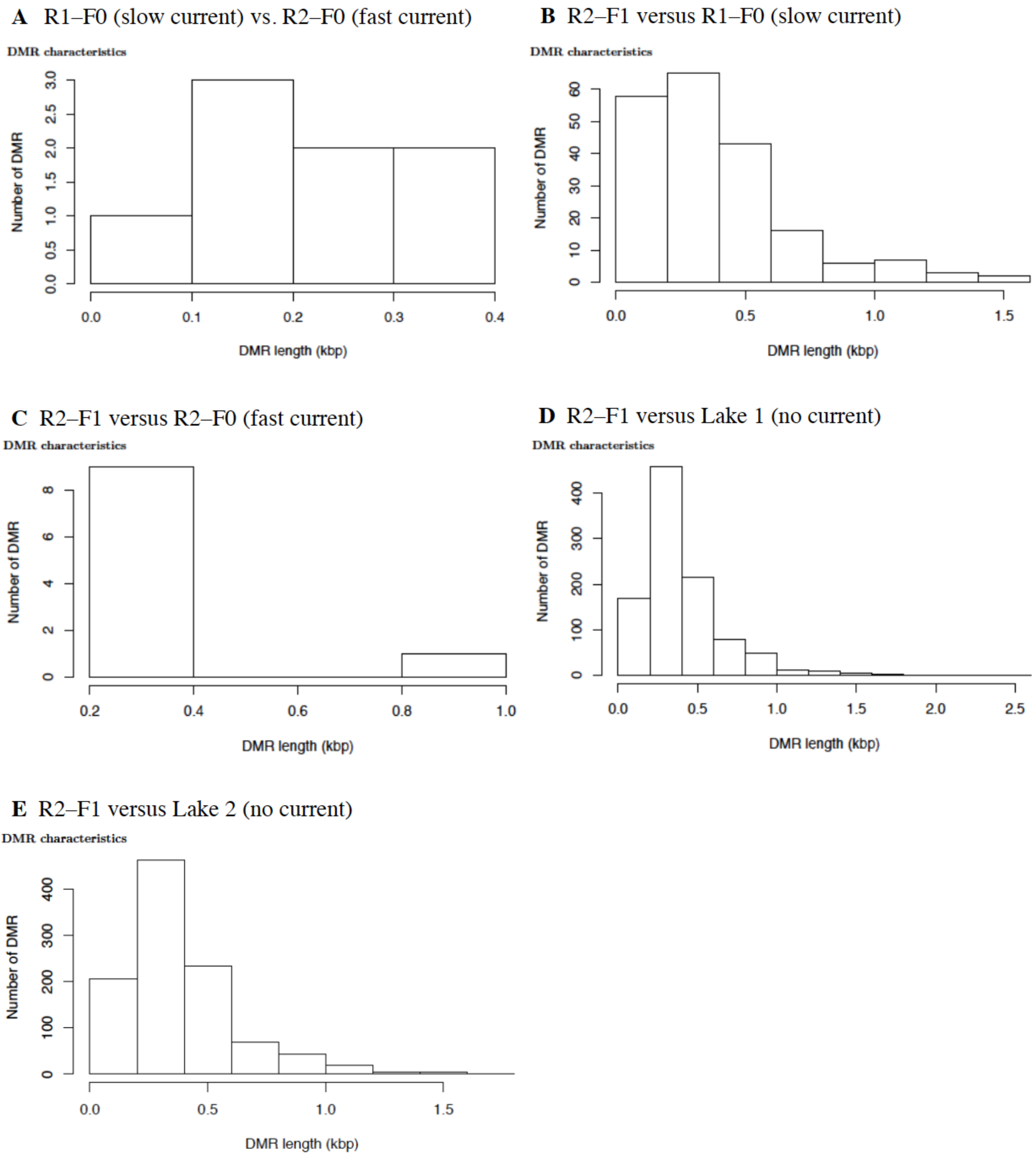

Supplemental Figure S7

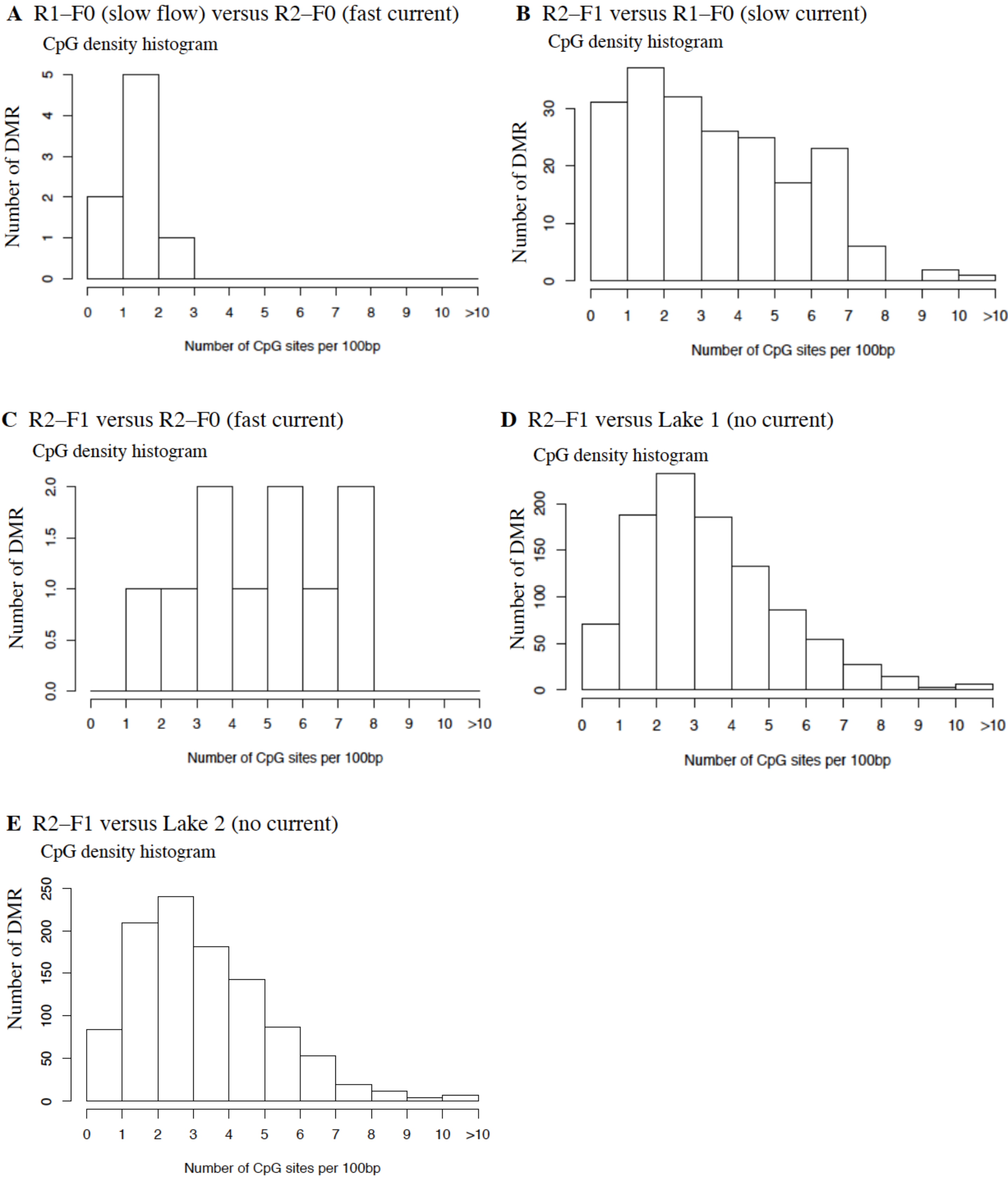

Supplemental Table S1

Category		Comparison	p adj.
**Field vs. Field**	Lake vs. Lake	L1 vs. L2	0.884
	River vs. River	R1 vs. R2	0.0415*
	Lake vs. River	L1 vs. R1	7.92e-13*
		L1 vs R2	7.24e-13*
		L2 vs. R1	7.98e-13*
		L2 vs. R2	7.59e-13*
**Lab vs. Field**	R2-F1 vs. Field	R2-F1 vs. L1	7.91e-13*
		R2-F1 vs. L2	7.94e-13*
		R2-F1 vs. R1	0.797
		R2-F1 vs. R2	0.920
	R2-F2 vs. Field	R2-F2 vs. L1	7.91e-13*
		R2-F2 vs. L2	2.39e-13*
		R2-F2 vs. R1	0.993
		R2-F2 vs. R2	0.0127*
	R2-F3 vs. Field	R2-F3 vs. L1	7.94e-13*
		R2-F3 vs. L2	3.47e-11*
		R2-F3 vs. R1	0.979
		R2-F3 vs. R2	0.00621*
**Lab vs. Lab**		R2-F1 vs. R2-F2	0.486
		R2-F1 vs. R2-F3	0.999
		R2-F2 vs. R2-F3	0.385

Supplemental Table S2

A R1–F_0 (slow current) versus R2–F_0 (fast current)

P-value	All Window	Multiple Window
0.001	125	11
1e-04	23	4
**1e-05**	**8**	**4**
1e-06	3	1
1e-07	2	1

Number of significant windows	1	2
Number of DMR	4	4

B R2–F_1 versus R1–F_0 (slow current)

P-value	All Window	Multiple Window
0.001	1461	417
1e-04	472	108
**1e-05**	**200**	**41**
1e-06	95	18
1e-07	48	13

Number of significant windows	1	2	3
Number of DMR	159	28	9

C R2–F_1 versus R2–F_0 (fast current)

P-value	All Window	Multiple Window
0.001	1305	367
1e-04	68	6
**1e-05**	**10**	**1**
1e-06	0	0
1e-07	0	0

Number of significant windows	1	2
Number of DMR	9	1

D R2–F_1 versus L1-F_0 (no current)

P-value	All Window	Multiple Window
0.001	3630	1507
1e-04	1842	707
**1e-05**	**1000**	**340**
1e-06	587	184
1e-07	350	106

Number of significant windows	1	2	3
Number of DMR	660	236	68

E R2–F_1 versus L2-F_0 (no current)

P-value	All Window	Multiple Window
0.001	3733	1539
1e-04	1900	707
**1e-05**	**1043**	**377**
1e-06	595	203
1e-07	369	125

Number of significant windows	1	2	3	≥4
Number of DMR	666	266	73	38

Supplemental Table S3
R1-F0 vs R2-F0 DMR Table 1e-05

DMR Name	Chr	Start	Stop	Length	# Sig Win	minP	CpG #	CpG Density	Gene Annotation	Gene Category
DMRcontig00982:2103401	contig00982	2103401	2103800	400	2	1.48E-06	8	2	zbtb8os	Transcription
DMRcontig01749:3631101	contig01749	3631101	3631400	300	2	5.48E-06	5	1.666666667	NA	NA
DMRcontig10273:18040401	contig10273	18040401	18040600	200	1	5.65E-06	2	1	NA	NA
DMRcontig13936:23548401	contig13936	23548401	23548600	200	1	6.49E-06	4	2	LOC107987209;LOC107984688;LOC108168064;LOC107973554;HOXD10;Spon1;PDE1B	Development;Cytoskeleton;Signaling;Extracellular Matrix
DMRcontig15247:25481801	contig15247	25481801	25482200	400	1	3.87E-06	7	1.75	NA	NA
DMRcontig20610:33289320	contig20610	33289320	33289500	181	2	4.10E-14	0	0	NA	NA
DMRcontig28086:42442715	contig28086	42442715	42442775	61	1	4.88E-07	1	1.639344262	NA	NA
DMRcontig51282:73236101	contig51282	73236101	73236311	211	2	1.94E-13	6	2.843601896	NA	NA

Supplemental Table S4
R2-F1 vs R2-F0 DMR Table 1e-05

DMR Name	Chr	Start	Stop	Length	# Sig Win	minP	CpG #	CpG Density	Gene Annotation	Gene Category
DMRcontig00696:1519601	contig00696	1519601	1520600	1000	2	1.98E-06	15	1.5	NA	NA
DMRcontig00949:2036601	contig00949	2036601	2036971	371	1	9.81E-06	15	4.043126685	LOC101686556;RPS14	Metabolism
DMRcontig06672:12297001	contig06672	12297001	12297200	200	1	2.05E-06	15	7.5	NA	NA
DMRcontig07647:13877001	contig07647	13877001	13877300	300	1	2.13E-06	11	3.666666667	NA	NA
DMRcontig09761:17247738	contig09761	17247738	17248000	263	1	6.91E-06	19	7.224334601	NA	NA
DMRcontig15313:25578941	contig15313	25578941	25579200	260	1	6.11E-06	15	5.769230769	Pepck	Metabolism
DMRcontig15371:25665001	contig15371	25665001	25665200	200	1	6.43E-06	13	6.5	NA	NA
DMRcontig17448:28664748	contig17448	28664748	28664962	215	1	5.74E-06	8	3.720930233	NA	NA
DMRcontig18476:30123801	contig18476	30123801	30124042	242	1	9.75E-06	7	2.892561983	NA	NA
DMRcontig19917:32268401	contig19917	32268401	32268800	400	1	2.55E-06	21	5.25	NA	NA

Supplemental Table S5
R2-F1 vs. Lake Lytle L1 DMR Table 1e-05

DMR Name	Chr	Start	Stop	Length	# Sig Win	minP	CpG #	CpG Density	Gene Annotation	Gene Category
DMRcontig00004:9955	contig00004	9955	12500	2546	2	1.29E-06	56	2.199528672	COX1;ATP8	Metabolism
DMRcontig00012:29574	contig00012	29574	31100	1527	7	4.28E-14	30	1.964636542	COX1	Metabolism
DMRcontig00029:67801	contig00029	67801	68800	1000	1	8.00E-06	34	3.4	NA	NA
DMRcontig00034:79101	contig00034	79101	80100	1000	3	5.42E-09	35	3.5	LOC109538865;ndufv2	Unknown;Metabolism
DMRcontig00041:97501	contig00041	97501	98000	500	1	2.34E-08	15	3	NA	NA
DMRcontig00049:115972	contig00049	115972	117298	1327	1	5.00E-06	27	2.034664657	ATP8;COX1	Metabolism
DMRcontig00059:142301	contig00059	142301	143530	1230	2	2.97E-11	77	6.260162602	NA	NA
DMRcontig00069:166301	contig00069	166301	168047	1747	1	8.31E-06	86	4.922724671	ddx39ab;Ddx39b	Development
DMRcontig00087:207101	contig00087	207101	208000	900	3	1.17E-08	35	3.888888889	LOC107219277	Cytoskeleton
DMRcontig00092:218401	contig00092	218401	219300	900	1	1.95E-06	17	1.888888889	NA	NA
DMRcontig00105:246801	contig00105	246801	247300	500	2	9.82E-07	18	3.6	NA	NA
DMRcontig00155:360401	contig00155	360401	361200	800	1	1.72E-06	6	0.75	NA	NA
DMRcontig00215:494001	contig00215	494001	494200	200	1	6.94E-06	6	3	NA	NA
DMRcontig00246:562701	contig00246	562701	563200	500	1	8.33E-06	8	1.6	NA	NA
DMRcontig00259:591301	contig00259	591301	592000	700	1	8.38E-06	23	3.285714286	NA	NA
DMRcontig00279:634537	contig00279	634537	635600	1064	2	9.61E-08	11	1.033834586	ATP8	Metabolism
DMRcontig00285:648209	contig00285	648209	649131	923	5	1.25E-09	17	1.841820152	COX1	Metabolism
DMRcontig00335:761401	contig00335	761401	761600	200	1	6.34E-06	14	7	LOC102794729;rpl23a	Translation
DMRcontig00386:868919	contig00386	868919	869200	282	2	7.04E-23	4	1.418439716	NA	NA
DMRcontig00396:891101	contig00396	891101	891600	500	3	3.88E-09	13	2.6	NA	NA
DMRcontig00417:936401	contig00417	936401	937400	1000	3	8.14E-08	60	6	NA	NA
DMRcontig00428:961001	contig00428	961001	961600	600	1	3.72E-06	15	2.5	SPRG_12594	Cytoskeleton
DMRcontig00446:999201	contig00446	999201	999400	200	1	7.55E-07	11	5.5	NA	NA
DMRcontig00449:1005054	contig00449	1005054	1005700	647	2	6.93E-12	51	7.882534776	NA	NA
DMRcontig00472:1056001	contig00472	1056001	1056300	300	1	3.81E-06	3	1	NA	NA
DMRcontig00498:1111401	contig00498	1111401	1111800	400	1	8.61E-07	22	5.5	NA	NA
DMRcontig00508:1132301	contig00508	1132301	1133700	1400	2	3.46E-08	66	4.714285714	DDOST	Metabolism
DMRcontig00522:1161200	contig00522	1161200	1161900	701	2	1.33E-10	12	1.711840228	NA	NA
DMRcontig00524:1165101	contig00524	1165101	1166100	1000	2	1.18E-08	67	6.7	NA	NA
DMRcontig00546:1210301	contig00546	1210301	1210500	200	1	7.86E-09	5	2.5	LOC106461013	Unknown
DMRcontig00548:1213301	contig00548	1213301	1214000	700	1	6.20E-08	18	2.571428571	NA	NA
DMRcontig00550:1217701	contig00550	1217701	1218100	400	1	9.01E-06	5	1.25	NA	NA
DMRcontig00558:1235101	contig00558	1235101	1235526	426	1	9.50E-06	25	5.868544601	NA	NA
DMRcontig00563:1245501	contig00563	1245501	1246200	700	1	3.75E-06	31	4.428571429	NA	NA
DMRcontig00576:1273401	contig00576	1273401	1274800	1400	1	1.02E-07	95	6.785714286	NA	NA
DMRcontig00589:1301001	contig00589	1301001	1301500	500	1	5.37E-06	9	1.8	NA	NA
DMRcontig00638:1403813	contig00638	1403813	1404200	388	3	1.84E-10	11	2.835051546	Smp_212110.1	Signaling
DMRcontig00665:1457201	contig00665	1457201	1458000	800	1	1.05E-06	20	2.5	NA	NA
DMRcontig00711:1552501	contig00711	1552501	1552800	300	3	9.73E-11	13	4.333333333	LOC101857978	Binding Protein
DMRcontig00731:1594925	contig00731	1594925	1595900	976	1	2.31E-06	35	3.586065574	NA	NA
DMRcontig00739:1612601	contig00739	1612601	1613600	1000	2	3.38E-08	6	0.6	NA	NA
DMRcontig00752:1640301	contig00752	1640301	1640500	200	1	4.50E-06	5	2.5	LOC106951031;EIF5A	Translation
DMRcontig00763:1662301	contig00763	1662301	1662600	300	3	2.20E-12	18	6	NA	NA
DMRcontig00767:1670201	contig00767	1670201	1671700	1500	2	5.76E-09	8	0.533333333	Gm7993;Cnot6l	Transcription;Metabolism
DMRcontig00797:1729601	contig00797	1729601	1729900	300	1	1.41E-06	20	6.666666667	NA	NA
DMRcontig00818:1771101	contig00818	1771101	1771300	200	1	2.05E-06	10	5	NA	NA
DMRcontig00832:1798418	contig00832	1798418	1798800	383	1	1.70E-07	8	2.088772846	NA	NA
DMRcontig00873:1881070	contig00873	1881070	1882300	1231	1	5.75E-06	29	2.355808286	NA	NA
DMRcontig00930:1999401	contig00930	1999401	1999800	400	1	1.40E-07	7	1.75	NA	NA
DMRcontig00941:2020117	contig00941	2020117	2020400	284	1	2.01E-06	15	5.281690141	NA	NA
DMRcontig00965:2068436	contig00965	2068436	2069300	865	5	1.27E-15	37	4.277456647	NA	NA
DMRcontig00982:2103401	contig00982	2103401	2103800	400	4	9.23E-32	8	2	zbtb8os	Transcription
DMRcontig01004:2147301	contig01004	2147301	2148100	800	2	3.80E-08	56	7	NA	NA
DMRcontig01028:2197501	contig01028	2197501	2198732	1232	1	3.60E-06	82	6.655844156	NA	NA
DMRcontig01042:2228201	contig01042	2228201	2228900	700	2	1.65E-09	21	3	NA	NA
DMRcontig01069:2284864	contig01069	2284864	2285920	1057	6	2.31E-10	16	1.51371807	COX1;ATP8	Metabolism
DMRcontig01099:2343201	contig01099	2343201	2344300	1100	1	8.14E-08	19	1.727272727	NA	NA
DMRcontig01101:2349401	contig01101	2349401	2350000	600	1	1.87E-06	10	1.666666667	NA	NA
DMRcontig01117:2381201	contig01117	2381201	2382271	1071	1	9.56E-07	48	4.481792717	NA	NA
DMRcontig01156:2458001	contig01156	2458001	2459569	1569	3	6.09E-07	44	2.804333971	NA	NA
DMRcontig01160:2466801	contig01160	2466801	2467755	955	2	7.88E-07	68	7.120418848	NA	NA
DMRcontig01167:2479901	contig01167	2479901	2480300	400	1	4.16E-06	19	4.75	LOC105888664	Epigenetic

DMRcontig01178:2502001	contig01178	2502001	2502100	100	1	7.64E-06	4	4	NA	NA
DMRcontig01200:2547401	contig01200	2547401	2548700	1300	1	1.91E-07	38	2.923076923	NA	NA
DMRcontig01222:2591484	contig01222	2591484	2592400	917	1	3.73E-06	17	1.85387132	LOC101861190	Signaling
DMRcontig01287:2722901	contig01287	2722901	2723100	200	1	1.23E-06	10	5	NA	NA
DMRcontig01308:2763401	contig01308	2763401	2764100	700	4	6.63E-09	25	3.571428571	NA	NA
DMRcontig01314:2775501	contig01314	2775501	2775800	300	2	2.59E-08	16	5.333333333	NA	NA
DMRcontig01316:2779001	contig01316	2779001	2779700	700	1	1.28E-07	12	1.714285714	Ubl5	Protease
DMRcontig01356:2856101	contig01356	2856101	2856400	300	1	8.87E-06	24	8	NA	NA
DMRcontig01375:2894733	contig01375	2894733	2895000	268	1	2.39E-07	32	11.94029851	NA	NA
DMRcontig01450:3053701	contig01450	3053701	3054560	860	1	4.65E-07	45	5.23255814	NA	NA
DMRcontig01453:3059201	contig01453	3059201	3059900	700	2	1.45E-06	19	2.714285714	NA	NA
DMRcontig01509:3170901	contig01509	3170901	3171300	400	1	8.53E-07	10	2.5	NA	NA
DMRcontig01525:3201001	contig01525	3201001	3202000	1000	2	5.24E-07	38	3.8	NA	NA
DMRcontig01567:3282801	contig01567	3282801	3283600	800	1	2.06E-07	38	4.75	LOC101863981	Transcription
DMRcontig01603:3349012	contig01603	3349012	3349600	589	3	4.46E-09	18	3.056027165	NA	NA
DMRcontig01607:3356220	contig01607	3356220	3357100	881	1	4.02E-10	19	2.156640182	NA	NA
DMRcontig01631:3403401	contig01631	3403401	3403700	300	2	1.54E-06	26	8.666666667	NA	NA
DMRcontig01649:3437001	contig01649	3437001	3437394	394	3	1.85E-08	20	5.076142132	NA	NA
DMRcontig01707:3551459	contig01707	3551459	3551700	242	1	1.33E-07	21	8.67768595	NA	NA
DMRcontig01741:3615569	contig01741	3615569	3616000	432	2	1.04E-08	12	2.777777778	COX1	Metabolism
DMRcontig01749:3631057	contig01749	3631057	3632100	1044	5	4.39E-35	27	2.586206897	NA	NA
DMRcontig01780:3692502	contig01780	3692502	3692700	199	2	2.58E-07	4	2.010050251	NA	NA
DMRcontig01790:3712001	contig01790	3712001	3712300	300	1	9.47E-09	11	3.666666667	NA	NA
DMRcontig01793:3717901	contig01793	3717901	3719000	1100	1	4.14E-07	34	3.090909091	NA	NA
DMRcontig01796:3724601	contig01796	3724601	3725091	491	1	1.43E-07	16	3.258655804	NA	NA
DMRcontig01808:3748501	contig01808	3748501	3748967	467	1	1.02E-06	1	0.214132762	NA	NA
DMRcontig01829:3789601	contig01829	3789601	3790600	1000	2	1.86E-08	27	2.7	NA	NA
DMRcontig01842:3816501	contig01842	3816501	3817100	600	1	5.34E-07	30	5	NA	NA
DMRcontig01884:3896701	contig01884	3896701	3897500	800	2	9.59E-08	15	1.875	NA	NA
DMRcontig01945:4014201	contig01945	4014201	4014500	300	2	5.01E-08	16	5.333333333	Dvir\GJ16291	Unknown
DMRcontig01964:4053401	contig01964	4053401	4054140	740	1	1.35E-07	17	2.297297297	NA	NA
DMRcontig01984:4090703	contig01984	4090703	4091700	998	1	9.29E-06	29	2.905811623	NA	NA
DMRcontig01987:4096801	contig01987	4096801	4097454	654	1	6.09E-07	26	3.975535168	NA	NA
DMRcontig01988:4098601	contig01988	4098601	4099100	500	2	4.54E-10	22	4.4	NA	NA
DMRcontig02044:4206201	contig02044	4206201	4206500	300	1	4.73E-08	26	8.666666667	NA	NA
DMRcontig02051:4218701	contig02051	4218701	4219100	400	1	4.03E-06	14	3.5	NA	NA
DMRcontig02055:4225706	contig02055	4225706	4226207	502	4	2.09E-09	8	1.593625498	ATP8;COX1	Metabolism
DMRcontig02059:4232201	contig02059	4232201	4233000	800	1	1.78E-06	41	5.125	LOC101851338;PPP2R1A	Signaling
DMRcontig02089:4292001	contig02089	4292001	4292806	806	1	7.28E-06	18	2.23325062	NA	NA
DMRcontig02101:4316001	contig02101	4316001	4316300	300	1	1.22E-06	19	6.333333333	LOC101846824	Growth Factors & Cytokines
DMRcontig02110:4333849	contig02110	4333849	4335600	1752	7	9.13E-10	61	3.48173516	NA	NA
DMRcontig02132:4373601	contig02132	4373601	4373900	300	1	3.14E-06	14	4.666666667	NA	NA
DMRcontig02181:4466266	contig02181	4466266	4466700	435	2	1.35E-07	14	3.218390805	NA	NA
DMRcontig02228:4553101	contig02228	4553101	4554000	900	2	1.12E-06	41	4.555555556	NA	NA
DMRcontig02231:4559301	contig02231	4559301	4559600	300	1	1.87E-08	12	4	NA	NA
DMRcontig02240:4575601	contig02240	4575601	4576300	700	1	1.95E-06	28	4	NA	NA
DMRcontig02261:4615501	contig02261	4615501	4616400	900	1	2.35E-07	36	4	NA	NA
DMRcontig02325:4731758	contig02325	4731758	4732100	343	1	2.11E-06	10	2.915451895	NA	NA
DMRcontig02340:4760301	contig02340	4760301	4760961	661	4	9.06E-16	8	1.210287443	COX1	Metabolism
DMRcontig02385:4842536	contig02385	4842536	4842900	365	2	4.58E-06	8	2.191780822	BRAFLDRAFT_81006	Unknown
DMRcontig02389:4850601	contig02389	4850601	4851000	400	3	7.59E-08	19	4.75	NA	NA
DMRcontig02392:4856663	contig02392	4856663	4856900	238	1	4.91E-07	3	1.260504202	NA	NA
DMRcontig02412:4894801	contig02412	4894801	4895300	500	1	3.49E-06	19	3.8	NA	NA
DMRcontig02429:4927305	contig02429	4927305	4927800	496	1	4.46E-07	10	2.016129032	NA	NA
DMRcontig02444:4954601	contig02444	4954601	4954875	275	1	7.52E-11	13	4.727272727	NA	NA
DMRcontig02446:4957716	contig02446	4957716	4958600	885	2	8.39E-08	17	1.920903955	NA	NA
DMRcontig02473:5006401	contig02473	5006401	5007132	732	2	1.02E-06	17	2.322404372	NA	NA
DMRcontig02495:5046401	contig02495	5046401	5046700	300	1	2.80E-06	12	4	NA	NA
DMRcontig02513:5075901	contig02513	5075901	5076175	275	2	1.10E-06	12	4.363636364	VOLCADRAFT_115993	Unknown
DMRcontig02533:5111001	contig02533	5111001	5111400	400	1	9.31E-06	11	2.75	NA	NA
DMRcontig02574:5189838	contig02574	5189838	5190782	945	1	6.34E-06	18	1.904761905	NA	NA
DMRcontig02629:5292519	contig02629	5292519	5293900	1382	1	5.09E-08	50	3.617945007	LOC106052646;rab21	Signaling
DMRcontig02636:5307359	contig02636	5307359	5307600	242	1	2.76E-06	7	2.892561983	NA	NA
DMRcontig02664:5359142	contig02664	5359142	5359600	459	1	2.65E-06	11	2.396514161	NA	NA
DMRcontig02696:5418001	contig02696	5418001	5418500	500	1	2.14E-06	11	2.2	NA	NA

DMRcontig02717:5455017	contig02717	5455017	5455400	384	2	1.81E-07	12	3.125	NA	NA
DMRcontig02744:5505401	contig02744	5505401	5505500	100	1	4.19E-07	6	6	NA	NA
DMRcontig02745:5506504	contig02745	5506504	5506900	397	2	6.74E-07	13	3.274559194	NA	NA
DMRcontig02757:5527701	contig02757	5527701	5528400	700	1	1.55E-06	41	5.857142857	LOC105896849	Metabolism
DMRcontig02779:5568101	contig02779	5568101	5569600	1500	1	1.74E-08	46	3.066666667	NA	NA
DMRcontig02800:5607701	contig02800	5607701	5608100	400	1	5.84E-08	23	5.75	NA	NA
DMRcontig02864:5721601	contig02864	5721601	5722100	500	3	8.31E-09	7	1.4	NA	NA
DMRcontig02932:5851901	contig02932	5851901	5852800	900	3	1.26E-06	28	3.111111111	NA	NA
DMRcontig02979:5933301	contig02979	5933301	5934000	700	2	1.90E-09	18	2.571428571	NA	NA
DMRcontig03104:6157901	contig03104	6157901	6158400	500	2	4.56E-12	9	1.8	NA	NA
DMRcontig03129:6204155	contig03129	6204155	6204818	664	1	8.09E-06	16	2.409638554	LOC105371236	Unknown
DMRcontig03173:6282963	contig03173	6282963	6283300	338	2	3.16E-08	26	7.692307692	NA	NA
DMRcontig03205:6339801	contig03205	6339801	6340100	300	1	6.21E-07	4	1.333333333	NA	NA
DMRcontig03213:6355001	contig03213	6355001	6355247	247	1	8.41E-06	5	2.024291498	NA	NA
DMRcontig03247:6415501	contig03247	6415501	6415900	400	1	5.67E-07	11	2.75	BRAFLDRAFT_74748	Unknown
DMRcontig03283:6481301	contig03283	6481301	6481600	300	1	5.36E-09	10	3.333333333	NA	NA
DMRcontig03285:6484701	contig03285	6484701	6485700	1000	1	1.34E-06	56	5.6	LOC100160485	Unknown
DMRcontig03333:6569449	contig03333	6569449	6569800	352	1	6.99E-07	3	0.852272727	NA	NA
DMRcontig03357:6614201	contig03357	6614201	6614500	300	1	3.04E-06	24	8	NA	NA
DMRcontig03390:6673801	contig03390	6673801	6674100	300	2	1.61E-06	14	4.666666667	NA	NA
DMRcontig03415:6717401	contig03415	6717401	6717800	400	2	2.47E-10	20	5	NA	NA
DMRcontig03435:6751501	contig03435	6751501	6752034	534	1	3.94E-06	8	1.498127341	NA	NA
DMRcontig03480:6830833	contig03480	6830833	6831435	603	3	1.85E-09	31	5.140961857	NA	NA
DMRcontig03487:6843701	contig03487	6843701	6844400	700	1	3.73E-08	20	2.857142857	NA	NA
DMRcontig03515:6891601	contig03515	6891601	6892900	1300	2	1.70E-07	26	2	LOC102350821;ppp2cb	Signaling
DMRcontig03521:6903601	contig03521	6903601	6903900	300	2	4.46E-08	8	2.666666667	NA	NA
DMRcontig03524:6909201	contig03524	6909201	6909800	600	2	6.85E-08	20	3.333333333	NA	NA
DMRcontig03533:6925116	contig03533	6925116	6925400	285	2	1.17E-06	19	6.666666667	NA	NA
DMRcontig03534:6926901	contig03534	6926901	6927581	681	1	4.43E-06	18	2.643171806	NA	NA
DMRcontig03555:6965375	contig03555	6965375	6966100	726	1	2.28E-09	26	3.581267218	NA	NA
DMRcontig03558:6970801	contig03558	6970801	6971200	400	1	1.02E-06	11	2.75	NA	NA
DMRcontig03569:6991201	contig03569	6991201	6992000	800	1	1.55E-06	31	3.875	NA	NA
DMRcontig03574:6999401	contig03574	6999401	7000100	700	1	2.59E-06	23	3.285714286	Ssc5d	Unknown
DMRcontig03586:7019401	contig03586	7019401	7020000	600	1	3.00E-06	10	1.666666667	NA	NA
DMRcontig03624:7084001	contig03624	7084001	7084388	388	2	2.02E-14	33	8.505154639	NA	NA
DMRcontig03663:7151701	contig03663	7151701	7152834	1134	4	4.48E-08	26	2.292768959	NA	NA
DMRcontig03710:7238441	contig03710	7238441	7238700	260	2	1.45E-07	2	0.769230769	NA	NA
DMRcontig03723:7259757	contig03723	7259757	7260200	444	1	7.10E-07	19	4.279279279	NA	NA
DMRcontig03824:7439701	contig03824	7439701	7440000	300	1	1.09E-10	26	8.666666667	NA	NA
DMRcontig03844:7476618	contig03844	7476618	7477300	683	2	6.74E-11	17	2.489019034	NA	NA
DMRcontig03861:7507401	contig03861	7507401	7507900	500	1	9.90E-06	21	4.2	NA	NA
DMRcontig03864:7512043	contig03864	7512043	7512300	258	1	2.83E-06	18	6.976744186	LOC101855053	Epigenetic
DMRcontig03887:7551659	contig03887	7551659	7552200	542	3	2.51E-09	31	5.719557196	NA	NA
DMRcontig03903:7579301	contig03903	7579301	7579800	500	1	9.64E-06	23	4.6	NA	NA
DMRcontig03911:7593555	contig03911	7593555	7594100	546	1	1.63E-07	10	1.831501832	NA	NA
DMRcontig03945:7652201	contig03945	7652201	7652500	300	1	1.05E-06	1	0.333333333	NA	NA
DMRcontig03961:7678974	contig03961	7678974	7679300	327	1	4.67E-06	10	3.058103976	NA	NA
DMRcontig03976:7705535	contig03976	7705535	7705900	366	1	2.36E-07	11	3.005464481	NA	NA
DMRcontig03981:7713516	contig03981	7713516	7713900	385	1	7.08E-06	6	1.558441558	NA	NA
DMRcontig04035:7812601	contig04035	7812601	7812800	200	2	1.71E-10	7	3.5	NA	NA
DMRcontig04057:7850468	contig04057	7850468	7850900	433	2	1.58E-10	9	2.07852194	NA	NA
DMRcontig04064:7861801	contig04064	7861801	7862353	553	1	2.48E-06	34	6.148282098	NA	NA
DMRcontig04084:7897235	contig04084	7897235	7897663	429	1	2.65E-08	14	3.263403263	NA	NA
DMRcontig04117:7955443	contig04117	7955443	7956000	558	1	5.04E-08	33	5.913978495	NA	NA
DMRcontig04156:8022201	contig04156	8022201	8022900	700	2	1.96E-06	13	1.857142857	COX1	Metabolism
DMRcontig04167:8041601	contig04167	8041601	8041900	300	1	3.94E-07	14	4.666666667	NA	NA
DMRcontig04219:8130901	contig04219	8130901	8131094	194	1	8.71E-07	9	4.639175258	NA	NA
DMRcontig04261:8202801	contig04261	8202801	8203435	635	3	7.51E-12	29	4.566929134	NA	NA
DMRcontig04266:8211901	contig04266	8211901	8212100	200	1	8.76E-06	4	2	NA	NA
DMRcontig04275:8227901	contig04275	8227901	8228400	500	1	1.95E-06	26	5.2	NA	NA
DMRcontig04287:8249401	contig04287	8249401	8249700	300	3	8.71E-07	14	4.666666667	NA	NA
DMRcontig04344:8347801	contig04344	8347801	8348092	292	1	9.33E-07	9	3.082191781	NA	NA
DMRcontig04384:8420167	contig04384	8420167	8420400	234	1	2.32E-09	5	2.136752137	Gm11675;Gm46346;Myt1l	Unknown;Transcription
DMRcontig04415:8475301	contig04415	8475301	8475816	516	2	7.01E-07	15	2.906976744	NA	NA
DMRcontig04487:8600801	contig04487	8600801	8601300	500	1	5.31E-06	7	1.4	NA	NA
DMRcontig04530:8675901	contig04530	8675901	8676200	300	1	1.23E-07	5	1.666666667	NA	NA

DMRcontig04542:8697431	contig04542	8697431	8698089	659	2	4.49E-12	26	3.945371775	NA	NA
DMRcontig04554:8717113	contig04554	8717113	8717300	188	1	3.35E-12	12	6.382978723	NA	NA
DMRcontig04621:8831001	contig04621	8831001	8831700	700	5	1.22E-12	46	6.571428571	NA	NA
DMRcontig04678:8928347	contig04678	8928347	8929200	854	2	3.69E-14	15	1.756440281	LOC101070845	Metabolism
DMRcontig04714:8993250	contig04714	8993250	8993700	451	3	1.66E-09	13	2.88248337	NA	NA
DMRcontig04741:9038773	contig04741	9038773	9039000	228	1	3.04E-06	1	0.438596491	NA	NA
DMRcontig04752:9058601	contig04752	9058601	9059600	1000	1	1.73E-06	39	3.9	NA	NA
DMRcontig04758:9070212	contig04758	9070212	9070500	289	3	6.07E-07	12	4.152249135	NA	NA
DMRcontig04767:9087101	contig04767	9087101	9087700	600	1	1.62E-06	12	2	LOC107162375	Epigenetic
DMRcontig04782:9113601	contig04782	9113601	9114300	700	1	2.09E-07	26	3.714285714	NA	NA
DMRcontig04899:9310301	contig04899	9310301	9310600	300	1	1.06E-08	6	2	NA	NA
DMRcontig04930:9362721	contig04930	9362721	9363300	580	1	1.26E-06	17	2.931034483	NA	NA
DMRcontig04949:9395301	contig04949	9395301	9395740	440	1	1.78E-07	13	2.954545455	NA	NA
DMRcontig04962:9416801	contig04962	9416801	9417000	200	1	8.23E-07	6	3	NA	NA
DMRcontig05018:9513737	contig05018	9513737	9514100	364	1	7.11E-07	26	7.142857143	NA	NA
DMRcontig05024:9524101	contig05024	9524101	9524400	300	1	9.00E-06	6	2	LOC105777419	Metabolism
DMRcontig05029:9532101	contig05029	9532101	9532480	380	2	1.61E-08	10	2.631578947	NA	NA
DMRcontig05064:9591401	contig05064	9591401	9592100	700	2	6.43E-06	20	2.857142857	NA	NA
DMRcontig05096:9648301	contig05096	9648301	9649000	700	1	1.39E-06	28	4	NA	NA
DMRcontig05156:9754512	contig05156	9754512	9755400	889	1	1.21E-07	28	3.149606299	NA	NA
DMRcontig05181:9797201	contig05181	9797201	9797500	300	1	7.02E-07	4	1.333333333	NA	NA
DMRcontig05196:9823201	contig05196	9823201	9824100	900	1	9.29E-06	51	5.666666667	NA	NA
DMRcontig05207:9841701	contig05207	9841701	9842175	475	1	7.95E-06	12	2.526315789	NA	NA
DMRcontig05209:9845601	contig05209	9845601	9846200	600	1	4.17E-07	30	5	NA	NA
DMRcontig05235:9887262	contig05235	9887262	9888200	939	4	5.55E-11	23	2.449414271	NA	NA
DMRcontig05242:9900401	contig05242	9900401	9901600	1200	1	6.87E-07	66	5.5	NA	NA
DMRcontig05252:9917701	contig05252	9917701	9918100	400	1	1.99E-06	10	2.5	NA	NA
DMRcontig05295:9989901	contig05295	9989901	9990544	644	1	7.88E-07	21	3.260869565	NA	NA
DMRcontig05316:10025807	contig05316	10025807	10026000	194	1	1.92E-07	5	2.577319588	NA	NA
DMRcontig05357:10096701	contig05357	10096701	10097000	300	1	5.87E-12	14	4.666666667	NA	NA
DMRcontig05360:10101301	contig05360	10101301	10101571	271	1	1.86E-07	5	1.84501845	NA	NA
DMRcontig05367:10114101	contig05367	10114101	10114500	400	1	5.71E-06	27	6.75	NA	NA
DMRcontig05467:10281001	contig05467	10281001	10281399	399	1	8.59E-06	9	2.255639098	NA	NA
DMRcontig05494:10326424	contig05494	10326424	10326700	277	2	1.26E-10	6	2.166064982	NA	NA
DMRcontig05509:10352001	contig05509	10352001	10352500	500	1	2.01E-06	10	2	NA	NA
DMRcontig05519:10370203	contig05519	10370203	10370900	698	1	6.94E-10	30	4.297994269	NA	NA
DMRcontig05538:10402101	contig05538	10402101	10402502	402	1	3.35E-07	28	6.965174129	NA	NA
DMRcontig05574:10465201	contig05574	10465201	10465400	200	1	8.40E-06	4	2	NA	NA
DMRcontig05596:10501001	contig05596	10501001	10501329	329	2	4.90E-09	6	1.823708207	NA	NA
DMRcontig05597:10502330	contig05597	10502330	10502800	471	1	2.05E-07	10	2.123142251	NA	NA
DMRcontig05622:10544001	contig05622	10544001	10544690	690	2	3.22E-09	12	1.739130435	NA	NA
DMRcontig05623:10545701	contig05623	10545701	10546100	400	1	8.87E-08	7	1.75	NA	NA
DMRcontig05643:10577927	contig05643	10577927	10578600	674	1	3.11E-06	10	1.483679525	NA	NA
DMRcontig05671:10624615	contig05671	10624615	10625263	649	3	9.68E-08	13	2.003081664	NA	NA
DMRcontig05708:10686901	contig05708	10686901	10687379	479	1	2.30E-06	9	1.878914405	NA	NA
DMRcontig05738:10737801	contig05738	10737801	10738723	923	1	1.09E-06	28	3.033586132	NA	NA
DMRcontig05763:10780301	contig05763	10780301	10780800	500	1	7.78E-06	16	3.2	NA	NA
DMRcontig05789:10824488	contig05789	10824488	10824700	213	1	7.50E-07	11	5.164319249	NA	NA
DMRcontig05794:10832701	contig05794	10832701	10833000	300	2	1.99E-09	5	1.666666667	NA	NA
DMRcontig05822:10880201	contig05822	10880201	10880500	300	1	7.27E-09	6	2	NA	NA
DMRcontig05826:10887401	contig05826	10887401	10887520	120	1	1.92E-07	2	1.666666667	NA	NA
DMRcontig05866:10956052	contig05866	10956052	10956400	349	2	2.14E-06	14	4.011461318	NA	NA
DMRcontig06037:11241001	contig06037	11241001	11241200	200	1	3.50E-09	11	5.5	NA	NA
DMRcontig06076:11307964	contig06076	11307964	11308500	537	3	6.39E-08	14	2.60707635	NA	NA
DMRcontig06079:11312801	contig06079	11312801	11313497	697	1	2.75E-06	32	4.591104735	NA	NA
DMRcontig06095:11338417	contig06095	11338417	11339000	584	2	5.07E-08	15	2.568493151	NA	NA
DMRcontig06133:11402379	contig06133	11402379	11403013	635	1	5.92E-07	11	1.732283465	NA	NA
DMRcontig06145:11422544	contig06145	11422544	11423000	457	1	2.25E-06	8	1.750547046	NA	NA
DMRcontig06163:11453701	contig06163	11453701	11454928	1228	1	1.55E-06	21	1.71009772	NA	NA
DMRcontig06167:11461501	contig06167	11461501	11461700	200	1	1.03E-07	11	5.5	NA	NA
DMRcontig06187:11495401	contig06187	11495401	11496100	700	1	1.03E-06	17	2.428571429	NA	NA
DMRcontig06247:11593801	contig06247	11593801	11594162	362	1	5.76E-06	8	2.209944751	NA	NA
DMRcontig06312:11703301	contig06312	11703301	11704200	900	1	3.76E-06	55	6.111111111	NA	NA
DMRcontig06350:11767201	contig06350	11767201	11767500	300	1	3.75E-06	6	2	NA	NA
DMRcontig06377:11812825	contig06377	11812825	11813800	976	1	6.96E-08	21	2.151639344	NA	NA
DMRcontig06412:11867764	contig06412	11867764	11868000	237	1	2.27E-07	0	0	NA	NA

DMRcontig06441:11916701	contig06441	11916701	11917000	300	1	6.73E-06	6	2	NA	NA
DMRcontig06442:11918301	contig06442	11918301	11918600	300	2	3.64E-12	10	3.333333333	NA	NA
DMRcontig06475:11973501	contig06475	11973501	11973900	400	2	9.56E-13	29	7.25	NA	NA
DMRcontig06497:12010504	contig06497	12010504	12011300	797	2	2.56E-06	16	2.007528231	NA	NA
DMRcontig06504:12021865	contig06504	12021865	12022800	936	1	3.20E-07	66	7.051282051	NA	NA
DMRcontig06512:12035501	contig06512	12035501	12036700	1200	1	2.96E-08	31	2.583333333	NA	NA
DMRcontig06553:12103501	contig06553	12103501	12103800	300	1	7.16E-06	15	5	NA	NA
DMRcontig06572:12134501	contig06572	12134501	12134900	400	2	9.37E-07	14	3.5	NA	NA
DMRcontig06591:12164401	contig06591	12164401	12164900	500	2	6.33E-15	23	4.6	NA	NA
DMRcontig06599:12178601	contig06599	12178601	12178900	300	1	1.16E-10	11	3.666666667	NA	NA
DMRcontig06605:12188701	contig06605	12188701	12188895	195	1	1.11E-08	7	3.58974359	NA	NA
DMRcontig06666:12286901	contig06666	12286901	12287471	571	4	6.43E-12	26	4.553415061	NA	NA
DMRcontig06701:12344501	contig06701	12344501	12345065	565	2	2.78E-10	6	1.061946903	NA	NA
DMRcontig06713:12364401	contig06713	12364401	12364900	500	2	1.03E-09	19	3.8	NA	NA
DMRcontig06717:12371501	contig06717	12371501	12371959	459	1	2.72E-07	4	0.871459695	UCRNP2_6158	Unknown
DMRcontig06740:12408491	contig06740	12408491	12408900	410	3	6.12E-10	15	3.658536585	NA	NA
DMRcontig06769:12454901	contig06769	12454901	12455300	400	1	3.43E-06	13	3.25	NA	NA
DMRcontig06775:12464375	contig06775	12464375	12465261	887	1	1.29E-06	50	5.636978579	NA	NA
DMRcontig06804:12512432	contig06804	12512432	12512700	269	2	1.59E-06	8	2.973977695	NA	NA
DMRcontig06817:12534701	contig06817	12534701	12535108	408	1	5.76E-07	12	2.941176471	NA	NA
DMRcontig06824:12546310	contig06824	12546310	12546500	191	1	4.99E-06	4	2.094240838	NA	NA
DMRcontig06845:12579601	contig06845	12579601	12579900	300	1	2.29E-11	22	7.333333333	NA	NA
DMRcontig06876:12629801	contig06876	12629801	12629900	100	1	4.42E-06	8	8	NA	NA
DMRcontig06877:12631524	contig06877	12631524	12632216	693	1	1.57E-06	34	4.906204906	NA	NA
DMRcontig06919:12700901	contig06919	12700901	12701200	300	1	1.88E-08	10	3.333333333	NA	NA
DMRcontig07045:12907601	contig07045	12907601	12908300	700	2	5.62E-06	3	0.428571429	NA	NA
DMRcontig07066:12941401	contig07066	12941401	12941700	300	1	1.66E-06	2	0.666666667	LOC106163552	Metabolism
DMRcontig07108:13010701	contig07108	13010701	13011600	900	1	1.32E-06	20	2.222222222	NA	NA
DMRcontig07149:13078001	contig07149	13078001	13078300	300	2	4.26E-14	4	1.333333333	NA	NA
DMRcontig07172:13114801	contig07172	13114801	13115261	461	2	1.63E-11	7	1.518438178	NA	NA
DMRcontig07187:13138601	contig07187	13138601	13139400	800	8	9.51E-16	31	3.875	NA	NA
DMRcontig07315:13344406	contig07315	13344406	13344885	480	1	2.20E-06	14	2.916666667	NA	NA
DMRcontig07318:13348701	contig07318	13348701	13349558	858	3	1.75E-07	16	1.864801865	NA	NA
DMRcontig07397:13474326	contig07397	13474326	13474747	422	2	3.97E-07	29	6.872037915	NA	NA
DMRcontig07413:13500301	contig07413	13500301	13500763	463	1	1.77E-08	14	3.023758099	NA	NA
DMRcontig07448:13555501	contig07448	13555501	13555900	400	1	2.05E-06	8	2	NA	NA
DMRcontig07458:13571501	contig07458	13571501	13572000	500	1	8.50E-06	22	4.4	NA	NA
DMRcontig07537:13698601	contig07537	13698601	13699000	400	2	1.79E-06	8	2	NA	NA
DMRcontig07544:13709301	contig07544	13709301	13709600	300	1	7.28E-09	13	4.333333333	NA	NA
DMRcontig07547:13714001	contig07547	13714001	13714673	673	3	1.47E-07	22	3.268945022	NA	NA
DMRcontig07566:13744301	contig07566	13744301	13744700	400	1	3.99E-06	22	5.5	ACTG1	Cytoskeleton
DMRcontig07587:13778901	contig07587	13778901	13779100	200	1	2.99E-08	3	1.5	NA	NA
DMRcontig07612:13821111	contig07612	13821111	13821500	390	1	5.33E-08	6	1.538461538	NA	NA
DMRcontig07639:13863401	contig07639	13863401	13863800	400	1	3.80E-06	16	4	NA	NA
DMRcontig07664:13903915	contig07664	13903915	13904300	386	2	2.27E-07	14	3.626943005	NA	NA
DMRcontig07680:13931801	contig07680	13931801	13932700	900	2	1.37E-06	12	1.333333333	NA	NA
DMRcontig07711:13982467	contig07711	13982467	13982988	522	3	6.86E-08	4	0.766283525	NA	NA
DMRcontig07714:13987701	contig07714	13987701	13988900	1200	3	8.04E-10	52	4.333333333	NA	NA
DMRcontig07825:14169297	contig07825	14169297	14170000	704	6	2.52E-13	15	2.130681818	COX1;ND3	Metabolism
DMRcontig07833:14182101	contig07833	14182101	14182800	700	1	6.09E-11	22	3.142857143	NA	NA
DMRcontig07851:14211101	contig07851	14211101	14211700	600	1	1.17E-09	17	2.833333333	NA	NA
DMRcontig07854:14215801	contig07854	14215801	14216000	200	1	4.25E-09	13	6.5	NA	NA
DMRcontig07912:14309601	contig07912	14309601	14309800	200	1	4.76E-06	6	3	NA	NA
DMRcontig07943:14356947	contig07943	14356947	14357200	254	1	1.55E-06	19	7.480314961	NA	NA
DMRcontig07963:14391501	contig07963	14391501	14392000	500	1	6.97E-06	21	4.2	NA	NA
DMRcontig08027:14495201	contig08027	14495201	14495600	400	2	2.85E-15	37	9.25	NA	NA
DMRcontig08038:14512724	contig08038	14512724	14513500	777	1	3.23E-06	51	6.563706564	NA	NA
DMRcontig08050:14532485	contig08050	14532485	14533000	516	1	4.45E-07	15	2.906976744	NA	NA
DMRcontig08206:14781330	contig08206	14781330	14781600	271	2	5.55E-16	12	4.42804428	NA	NA
DMRcontig08213:14793401	contig08213	14793401	14793800	400	1	4.06E-06	3	0.75	NA	NA
DMRcontig08238:14833601	contig08238	14833601	14834200	600	1	4.20E-07	6	1	NA	NA
DMRcontig08240:14836601	contig08240	14836601	14837000	400	1	2.50E-06	22	5.5	NA	NA
DMRcontig08282:14904801	contig08282	14904801	14906300	1500	1	3.19E-06	63	4.2	NA	NA
DMRcontig08324:14972601	contig08324	14972601	14972900	300	2	1.40E-09	8	2.666666667	VDAG_02253	DNA Repair
DMRcontig08350:15015785	contig08350	15015785	15016091	307	2	7.35E-09	5	1.628664495	NA	NA
DMRcontig08357:15026601	contig08357	15026601	15026900	300	1	5.98E-08	12	4	NA	NA

DMRcontig08377:15058601	contig08377	15058601	15059600	1000	3	2.75E-08	26	2.6	NA	NA
DMRcontig08400:15096101	contig08400	15096101	15096899	799	1	5.62E-06	38	4.755944931	NA	NA
DMRcontig08412:15114788	contig08412	15114788	15115240	453	1	8.05E-07	11	2.428256071	NA	NA
DMRcontig08446:15171250	contig08446	15171250	15171500	251	2	1.67E-06	1	0.398406375	NA	NA
DMRcontig08460:15193601	contig08460	15193601	15193800	200	1	5.58E-06	6	3	NA	NA
DMRcontig08492:15243901	contig08492	15243901	15244082	182	1	2.42E-06	5	2.747252747	NA	NA
DMRcontig08515:15278503	contig08515	15278503	15278800	298	1	8.04E-06	22	7.382550336	NA	NA
DMRcontig08559:15348353	contig08559	15348353	15348600	248	1	8.02E-07	8	3.225806452	NA	NA
DMRcontig08569:15365901	contig08569	15365901	15366200	300	2	4.20E-07	11	3.666666667	NA	NA
DMRcontig08588:15396301	contig08588	15396301	15397031	731	2	2.08E-07	25	3.41997264	NA	NA
DMRcontig08670:15528041	contig08670	15528041	15529000	960	1	4.86E-06	31	3.229166667	NA	NA
DMRcontig08729:15621501	contig08729	15621501	15621900	400	1	8.80E-06	18	4.5	NA	NA
DMRcontig08824:15771101	contig08824	15771101	15771398	298	2	1.94E-07	5	1.677852349	NA	NA
DMRcontig08828:15777001	contig08828	15777001	15777311	311	1	3.67E-07	13	4.180064309	NA	NA
DMRcontig08897:15886374	contig08897	15886374	15886800	427	1	8.28E-06	14	3.278688525	NA	NA
DMRcontig08913:15910601	contig08913	15910601	15911279	679	5	2.78E-09	15	2.209131075	NA	NA
DMRcontig08953:15975301	contig08953	15975301	15975500	200	1	1.57E-06	8	4	NA	NA
DMRcontig09095:16196501	contig09095	16196501	16196700	200	1	2.66E-06	7	3.5	NA	NA
DMRcontig09129:16252401	contig09129	16252401	16252900	500	4	2.65E-22	33	6.6	CpipJ_CPIJ017745;FAXDC2	Metabolism
DMRcontig09205:16373301	contig09205	16373301	16373882	582	1	1.96E-07	29	4.982817869	NA	NA
DMRcontig09212:16385301	contig09212	16385301	16385599	299	1	7.45E-11	31	10.36789298	NA	NA
DMRcontig09219:16395701	contig09219	16395701	16396000	300	2	5.56E-15	15	5	NA	NA
DMRcontig09220:16397501	contig09220	16397501	16398100	600	3	8.30E-10	19	3.166666667	NA	NA
DMRcontig09249:16441101	contig09249	16441101	16441477	377	1	3.53E-06	16	4.24403183	NA	NA
DMRcontig09254:16449001	contig09254	16449001	16449900	900	1	4.55E-06	24	2.666666667	NA	NA
DMRcontig09547:16909701	contig09547	16909701	16910245	545	1	7.59E-06	27	4.95412844	NA	NA
DMRcontig09562:16933101	contig09562	16933101	16933620	520	1	4.34E-06	14	2.692307692	NA	NA
DMRcontig09570:16945429	contig09570	16945429	16945777	349	1	5.36E-08	8	2.29226361	NA	NA
DMRcontig09574:16951601	contig09574	16951601	16952343	743	1	8.13E-06	32	4.306864065	NA	NA
DMRcontig09586:16971019	contig09586	16971019	16971565	547	1	2.17E-08	20	3.65630713	NA	NA
DMRcontig09600:16994201	contig09600	16994201	16994400	200	1	9.31E-06	8	4	NA	NA
DMRcontig09632:17045001	contig09632	17045001	17045472	472	1	1.21E-06	10	2.118644068	NA	NA
DMRcontig09634:17047910	contig09634	17047910	17048500	591	4	9.39E-11	30	5.076142132	NA	NA
DMRcontig09660:17087701	contig09660	17087701	17088000	300	1	1.47E-07	12	4	LOC106070317	Unknown
DMRcontig09685:17126987	contig09685	17126987	17127500	514	3	3.20E-11	18	3.501945525	NA	NA
DMRcontig09699:17150701	contig09699	17150701	17151249	549	1	2.00E-07	8	1.4571949	NA	NA
DMRcontig09837:17366676	contig09837	17366676	17367000	325	1	3.72E-07	2	0.615384615	NA	NA
DMRcontig09843:17376209	contig09843	17376209	17376600	392	1	1.15E-06	4	1.020408163	NA	NA
DMRcontig09860:17402901	contig09860	17402901	17403288	388	1	9.25E-07	17	4.381443299	NA	NA
DMRcontig09899:17460248	contig09899	17460248	17460730	483	5	2.59E-13	9	1.863354037	NA	NA
DMRcontig09949:17536201	contig09949	17536201	17536564	364	1	2.98E-06	10	2.747252747	NA	NA
DMRcontig09973:17573501	contig09973	17573501	17574100	600	1	1.14E-06	29	4.833333333	NA	NA
DMRcontig09993:17606224	contig09993	17606224	17606764	541	2	6.72E-08	45	8.31792976	NA	NA
DMRcontig10093:17761801	contig10093	17761801	17762290	490	2	1.72E-06	6	1.224489796	NA	NA
DMRcontig10191:17914201	contig10191	17914201	17914900	700	2	5.09E-06	36	5.142857143	NA	NA
DMRcontig10208:17940478	contig10208	17940478	17940874	397	1	1.84E-06	17	4.282115869	NA	NA
DMRcontig10249:18003425	contig10249	18003425	18003871	447	1	4.75E-06	6	1.342281879	NA	NA
DMRcontig10293:18070532	contig10293	18070532	18071727	1196	7	2.61E-12	40	3.344481605	NA	NA
DMRcontig10295:18074101	contig10295	18074101	18074200	100	1	5.58E-06	3	3	NA	NA
DMRcontig10320:18110601	contig10320	18110601	18110900	300	1	1.86E-06	8	2.666666667	NA	NA
DMRcontig10423:18268501	contig10423	18268501	18268800	300	1	2.69E-06	7	2.333333333	NA	NA
DMRcontig10530:18430512	contig10530	18430512	18431200	689	2	2.32E-06	16	2.322206096	LOC105924796	Mitochondria
DMRcontig10533:18435301	contig10533	18435301	18435600	300	1	1.34E-07	26	8.666666667	NA	NA
DMRcontig10540:18446701	contig10540	18446701	18447000	300	2	1.97E-10	10	3.333333333	NA	NA
DMRcontig10597:18535134	contig10597	18535134	18535400	267	1	6.51E-06	4	1.498127341	NA	NA
DMRcontig10628:18582472	contig10628	18582472	18582800	329	1	9.86E-06	4	1.215805471	NA	NA
DMRcontig10772:18805801	contig10772	18805801	18806189	389	1	1.79E-07	9	2.313624679	NA	NA
DMRcontig10807:18860301	contig10807	18860301	18860643	343	1	3.03E-09	6	1.749271137	NA	NA
DMRcontig10811:18865901	contig10811	18865901	18866300	400	1	7.69E-06	16	4	NA	NA
DMRcontig10818:18875801	contig10818	18875801	18876040	240	1	6.23E-06	5	2.083333333	NA	NA
DMRcontig10834:18900001	contig10834	18900001	18900799	799	1	4.72E-11	36	4.50563204	NA	NA
DMRcontig10848:18921701	contig10848	18921701	18922098	398	2	1.22E-16	14	3.51758794	NA	NA
DMRcontig10862:18942798	contig10862	18942798	18943100	303	1	6.17E-06	8	2.640264026	NA	NA
DMRcontig10874:18960901	contig10874	18960901	18961300	400	1	8.79E-06	26	6.5	NA	NA
DMRcontig10877:18966001	contig10877	18966001	18966558	558	1	1.52E-06	15	2.688172043	NA	NA
DMRcontig10893:18989469	contig10893	18989469	18989900	432	2	5.98E-07	9	2.083333333	NA	NA

DMRcontig10937:19056818	contig10937	19056818	19057161	344	1	5.16E-06	17	4.941860465	NA	NA
DMRcontig10975:19114720	contig10975	19114720	19115000	281	2	3.29E-07	2	0.711743772	NA	NA
DMRcontig11028:19195859	contig11028	19195859	19196100	242	1	2.69E-07	6	2.479338843	NA	NA
DMRcontig11037:19209483	contig11037	19209483	19209700	218	1	1.68E-08	4	1.834862385	NA	NA
DMRcontig11064:19249601	contig11064	19249601	19250000	400	2	2.29E-06	8	2	NA	NA
DMRcontig11127:19344510	contig11127	19344510	19344800	291	1	5.26E-06	4	1.374570447	NA	NA
DMRcontig11155:19386368	contig11155	19386368	19386700	333	1	2.17E-08	4	1.201201201	NA	NA
DMRcontig11187:19434201	contig11187	19434201	19434600	400	1	1.08E-09	11	2.75	NA	NA
DMRcontig11287:19586901	contig11287	19586901	19587500	600	1	3.82E-06	18	3	NA	NA
DMRcontig11306:19616001	contig11306	19616001	19616400	400	1	9.68E-06	5	1.25	NA	NA
DMRcontig11308:19619301	contig11308	19619301	19619900	600	1	3.73E-06	33	5.5	NA	NA
DMRcontig11431:19802501	contig11431	19802501	19802900	400	2	4.52E-08	7	1.75	NA	NA
DMRcontig11459:19845817	contig11459	19845817	19846059	243	1	1.66E-07	5	2.057613169	NA	NA
DMRcontig11479:19875931	contig11479	19875931	19876700	770	1	4.20E-07	15	1.948051948	NA	NA
DMRcontig11518:19936693	contig11518	19936693	19936900	208	1	5.12E-06	8	3.846153846	NA	NA
DMRcontig11521:19940901	contig11521	19940901	19941199	299	1	4.47E-06	16	5.351170569	NA	NA
DMRcontig11529:19952401	contig11529	19952401	19952800	400	2	3.63E-08	25	6.25	NA	NA
DMRcontig11534:19960001	contig11534	19960001	19960500	500	2	4.55E-07	27	5.4	NA	NA
DMRcontig11547:19979201	contig11547	19979201	19979600	400	3	8.43E-07	16	4	NA	NA
DMRcontig11554:19989801	contig11554	19989801	19990200	400	1	6.30E-06	17	4.25	NA	NA
DMRcontig11557:19994201	contig11557	19994201	19994500	300	1	6.59E-07	16	5.333333333	NA	NA
DMRcontig11593:20046749	contig11593	20046749	20047184	436	5	4.19E-25	13	2.981651376	NA	NA
DMRcontig11622:20088329	contig11622	20088329	20088831	503	2	3.98E-08	17	3.37972167	NA	NA
DMRcontig11652:20133101	contig11652	20133101	20133800	700	1	4.48E-06	11	1.571428571	MONBRDRAFT_35219	Unknown
DMRcontig11731:20254201	contig11731	20254201	20254700	500	1	5.66E-06	34	6.8	NA	NA
DMRcontig11737:20263301	contig11737	20263301	20263500	200	1	7.17E-06	6	3	NA	NA
DMRcontig11781:20327801	contig11781	20327801	20328100	300	1	6.78E-06	9	3	CGB_A0010C;CGB_A6680C;GUITHDRAFT_104274	Transport;Translation;Unknown
DMRcontig11854:20440637	contig11854	20440637	20440800	164	1	4.36E-07	6	3.658536585	NA	NA
DMRcontig11877:20475901	contig11877	20475901	20476300	400	1	3.10E-06	16	4	NA	NA
DMRcontig11955:20591501	contig11955	20591501	20591800	300	1	7.69E-07	23	7.666666667	NA	NA
DMRcontig11987:20639001	contig11987	20639001	20639400	400	1	3.11E-06	14	3.5	NA	NA
DMRcontig12018:20685201	contig12018	20685201	20685400	200	1	7.33E-12	13	6.5	NA	NA
DMRcontig12021:20689621	contig12021	20689621	20689966	346	2	7.35E-07	13	3.757225434	NA	NA
DMRcontig12031:20704801	contig12031	20704801	20705019	219	1	4.38E-08	5	2.283105023	NA	NA
DMRcontig12042:20723404	contig12042	20723404	20723600	197	2	4.43E-11	3	1.52284264	NA	NA
DMRcontig12051:20736801	contig12051	20736801	20737100	300	1	7.62E-07	12	4	NA	NA
DMRcontig12082:20783501	contig12082	20783501	20783800	300	1	8.62E-07	13	4.333333333	NA	NA
DMRcontig12185:20937701	contig12185	20937701	20938132	432	1	3.39E-06	10	2.314814815	NA	NA
DMRcontig12215:20982931	contig12215	20982931	20983645	715	3	5.51E-10	11	1.538461538	NA	NA
DMRcontig12284:21087301	contig12284	21087301	21088387	1087	2	9.00E-07	63	5.795768169	NA	NA
DMRcontig12397:21260301	contig12397	21260301	21260716	416	1	7.08E-06	5	1.201923077	NA	NA
DMRcontig12426:21303145	contig12426	21303145	21303700	556	1	1.32E-06	12	2.158273381	NA	NA
DMRcontig12455:21348801	contig12455	21348801	21349300	500	1	5.41E-07	15	3	NA	NA
DMRcontig12524:21452301	contig12524	21452301	21452500	200	1	7.68E-06	11	5.5	NA	NA
DMRcontig12526:21455437	contig12526	21455437	21455900	464	1	2.94E-06	28	6.034482759	NA	NA
DMRcontig12611:21582101	contig12611	21582101	21582454	354	1	2.13E-07	12	3.389830508	NA	NA
DMRcontig12653:21643719	contig12653	21643719	21644184	466	1	1.24E-06	10	2.145922747	NA	NA
DMRcontig12690:21698851	contig12690	21698851	21699100	250	1	1.64E-07	3	1.2	NA	NA
DMRcontig12737:21768110	contig12737	21768110	21768600	491	3	1.70E-09	6	1.221995927	NA	NA
DMRcontig12764:21808316	contig12764	21808316	21808700	385	1	2.46E-06	5	1.298701299	NA	NA
DMRcontig12978:22123001	contig12978	22123001	22123300	300	1	9.60E-06	10	3.333333333	NA	NA
DMRcontig13033:22204443	contig13033	22204443	22204700	258	1	6.64E-06	1	0.387596899	NA	NA
DMRcontig13089:22287101	contig13089	22287101	22287700	600	3	9.32E-11	36	6	NA	NA
DMRcontig13121:22334801	contig13121	22334801	22335300	500	4	1.47E-21	34	6.8	NA	NA
DMRcontig13123:22338101	contig13123	22338101	22338500	400	1	5.81E-08	11	2.75	NA	NA
DMRcontig13124:22339638	contig13124	22339638	22339883	246	1	5.36E-11	8	3.25203252	NA	NA
DMRcontig13131:22350601	contig13131	22350601	22351059	459	1	2.69E-07	11	2.396514161	NA	NA
DMRcontig13141:22365643	contig13141	22365643	22366000	358	3	2.64E-10	15	4.189944134	NA	NA
DMRcontig13227:22490283	contig13227	22490283	22490500	218	1	1.91E-06	7	3.211009174	NA	NA
DMRcontig13259:22536401	contig13259	22536401	22536800	400	1	3.99E-07	11	2.75	LOC101858697	Binding Protein
DMRcontig13269:22552201	contig13269	22552201	22552493	293	1	1.16E-06	4	1.365187713	NA	NA
DMRcontig13281:22570601	contig13281	22570601	22570900	300	2	2.58E-07	3	1	NA	NA
DMRcontig13288:22581032	contig13288	22581032	22581517	486	4	1.48E-11	25	5.144032922	NA	NA
DMRcontig13291:22585501	contig13291	22585501	22585900	400	3	3.32E-07	13	3.25	NA	NA
DMRcontig13308:22610847	contig13308	22610847	22611000	154	1	5.45E-07	3	1.948051948	NA	NA

DMRcontig13359:22689301	contig13359	22689301	22689600	300	1	2.35E-08	4	1.333333333	NA	NA
DMRcontig13413:22768680	contig13413	22768680	22769200	521	1	2.07E-07	14	2.687140115	NA	NA
DMRcontig13467:22848001	contig13467	22848001	22848200	200	2	1.37E-11	7	3.5	NA	NA
DMRcontig13527:22937007	contig13527	22937007	22937200	194	1	5.46E-07	0	0	NA	NA
DMRcontig13580:23016553	contig13580	23016553	23016900	348	2	2.79E-09	24	6.896551724	NA	NA
DMRcontig13586:23025644	contig13586	23025644	23026200	557	1	4.44E-06	18	3.231597846	NA	NA
DMRcontig13619:23076502	contig13619	23076502	23076967	466	1	9.91E-06	14	3.004291845	NA	NA
DMRcontig13629:23091801	contig13629	23091801	23092359	559	1	5.60E-07	19	3.398926655	NA	NA
DMRcontig13644:23115548	contig13644	23115548	23115800	253	1	3.61E-06	3	1.185770751	NA	NA
DMRcontig13696:23192901	contig13696	23192901	23193122	222	1	4.41E-07	4	1.801801802	NA	NA
DMRcontig13717:23223645	contig13717	23223645	23224000	356	1	4.45E-07	7	1.966292135	NA	NA
DMRcontig13733:23247301	contig13733	23247301	23247600	300	2	1.98E-08	12	4	NA	NA
DMRcontig13737:23253031	contig13737	23253031	23253674	644	1	3.27E-06	31	4.813664596	NA	NA
DMRcontig13756:23280401	contig13756	23280401	23280700	300	1	8.21E-09	15	5	NA	NA
DMRcontig13911:23511901	contig13911	23511901	23512200	300	1	4.00E-06	1	0.333333333	NA	NA
DMRcontig13936:23548143	contig13936	23548143	23548700	558	2	5.86E-09	11	1.971326165	LOC107987209;LOC107984688;LOC108168064;LOC107973554;HOXD10;Spon1;PDE1B	Development;Cytoskeleton;Unknown;Signaling
DMRcontig13944:23560801	contig13944	23560801	23561400	600	1	1.35E-06	18	3	NA	NA
DMRcontig13950:23570501	contig13950	23570501	23570645	145	1	3.79E-07	2	1.379310345	NA	NA
DMRcontig14002:23647101	contig14002	23647101	23647200	100	1	2.96E-06	0	0	NA	NA
DMRcontig14064:23738204	contig14064	23738204	23738500	297	2	3.41E-09	11	3.703703704	NA	NA
DMRcontig14077:23757501	contig14077	23757501	23757832	332	1	9.91E-06	12	3.614457831	NA	NA
DMRcontig14078:23759001	contig14078	23759001	23759141	141	1	7.94E-07	0	0	NA	NA
DMRcontig14090:23776301	contig14090	23776301	23776800	500	1	6.07E-06	13	2.6	Ubl5	Protease
DMRcontig14178:23907801	contig14178	23907801	23908000	200	1	2.99E-06	3	1.5	NA	NA
DMRcontig14218:23965716	contig14218	23965716	23966091	376	2	5.03E-09	3	0.79787234	NA	NA
DMRcontig14248:24009901	contig14248	24009901	24010400	500	1	3.82E-06	21	4.2	NA	NA
DMRcontig14287:24067301	contig14287	24067301	24067484	184	1	6.83E-06	5	2.717391304	NA	NA
DMRcontig14317:24109901	contig14317	24109901	24110200	300	1	6.61E-07	9	3	NA	NA
DMRcontig14318:24111208	contig14318	24111208	24111800	593	1	6.28E-07	39	6.576728499	THITE_2119671;THITE_2054382	Metabolism;Unknown
DMRcontig14382:24205923	contig14382	24205923	24206350	428	1	1.81E-08	16	3.738317757	NA	NA
DMRcontig14483:24355605	contig14483	24355605	24356169	565	3	2.94E-06	8	1.415929204	NA	NA
DMRcontig14531:24427501	contig14531	24427501	24427900	400	1	1.28E-07	10	2.5	NA	NA
DMRcontig14597:24524401	contig14597	24524401	24524948	548	3	2.30E-07	11	2.00729927	NA	NA
DMRcontig14615:24551401	contig14615	24551401	24551600	200	1	1.39E-07	2	1	NA	NA
DMRcontig14619:24557301	contig14619	24557301	24557600	300	1	3.84E-07	9	3	NA	NA
DMRcontig14671:24632667	contig14671	24632667	24633000	334	1	9.70E-06	2	0.598802395	NA	NA
DMRcontig14862:24917822	contig14862	24917822	24918519	698	1	3.55E-06	43	6.160458453	NA	NA
DMRcontig14872:24932201	contig14872	24932201	24932359	159	1	5.52E-06	3	1.886792453	NA	NA
DMRcontig14922:25005701	contig14922	25005701	25006000	300	1	6.86E-08	12	4	NA	NA
DMRcontig14953:25051602	contig14953	25051602	25051865	264	2	7.06E-07	13	4.924242424	NA	NA
DMRcontig14959:25060189	contig14959	25060189	25060400	212	1	1.17E-07	3	1.41509434	NA	NA
DMRcontig14960:25061601	contig14960	25061601	25061800	200	1	5.25E-08	4	2	LOC101415577;EFR3B	Unknown
DMRcontig14971:25077701	contig14971	25077701	25078100	400	2	2.58E-08	9	2.25	NA	NA
DMRcontig14979:25090301	contig14979	25090301	25090400	100	1	6.47E-06	1	1	NA	NA
DMRcontig15040:25178101	contig15040	25178101	25178395	295	2	3.54E-11	4	1.355932203	NA	NA
DMRcontig15044:25183608	contig15044	25183608	25183800	193	2	1.91E-06	5	2.590673575	NA	NA
DMRcontig15065:25214609	contig15065	25214609	25214800	192	2	4.25E-09	2	1.041666667	NA	NA
DMRcontig15083:25241901	contig15083	25241901	25242300	400	2	7.34E-07	7	1.75	NA	NA
DMRcontig15105:25274312	contig15105	25274312	25275000	689	2	4.48E-07	32	4.644412192	NA	NA
DMRcontig15250:25486401	contig15250	25486401	25486721	321	1	2.76E-06	16	4.984423676	NA	NA
DMRcontig15274:25521701	contig15274	25521701	25522300	600	3	7.24E-07	4	0.666666667	NA	NA
DMRcontig15287:25540701	contig15287	25540701	25541000	300	1	1.70E-06	4	1.333333333	NA	NA
DMRcontig15296:25553856	contig15296	25553856	25554000	145	2	2.11E-07	10	6.896551724	NA	NA
DMRcontig15314:25580889	contig15314	25580889	25581600	712	1	9.63E-07	23	3.230337079	NA	NA
DMRcontig15319:25588912	contig15319	25588912	25589376	465	4	1.43E-09	19	4.086021505	NA	NA
DMRcontig15350:25634039	contig15350	25634039	25634560	522	2	4.57E-08	16	3.0651341	NA	NA
DMRcontig15370:25663001	contig15370	25663001	25663366	366	1	1.04E-08	7	1.912568306	NA	NA
DMRcontig15378:25675381	contig15378	25675381	25675800	420	1	9.87E-06	10	2.380952381	NA	NA
DMRcontig15501:25856101	contig15501	25856101	25856384	284	1	3.10E-06	8	2.816901408	NA	NA
DMRcontig15561:25942423	contig15561	25942423	25942899	477	4	8.27E-19	4	0.838574423	NA	NA
DMRcontig15587:25979921	contig15587	25979921	25980351	431	1	1.86E-07	14	3.248259861	NA	NA
DMRcontig15604:26004793	contig15604	26004793	26005200	408	3	2.13E-11	9	2.205882353	NA	NA
DMRcontig15617:26023501	contig15617	26023501	26023805	305	1	8.45E-06	18	5.901639344	NA	NA

DMRcontig15627:26038318	contig15627	26038318	26038800	483	3	3.91E-13	12	2.48447205	NA	NA
DMRcontig15635:26049624	contig15635	26049624	26050062	439	1	1.17E-08	18	4.10022779	NA	NA
DMRcontig15692:26131101	contig15692	26131101	26131322	222	1	6.83E-08	8	3.603603604	NA	NA
DMRcontig15724:26178003	contig15724	26178003	26178200	198	1	2.50E-07	3	1.515151515	NA	NA
DMRcontig15731:26188008	contig15731	26188008	26188363	356	3	1.94E-08	17	4.775280899	LOC106177743;LOC106177744;LOC106177745	Epigenetic
DMRcontig15745:26207963	contig15745	26207963	26208500	538	2	4.50E-09	18	3.345724907	NA	NA
DMRcontig15783:26263503	contig15783	26263503	26263762	260	1	5.55E-07	6	2.307692308	NA	NA
DMRcontig15793:26277442	contig15793	26277442	26277900	459	2	9.77E-07	21	4.575163399	NA	NA
DMRcontig15839:26345437	contig15839	26345437	26345934	498	1	9.53E-06	23	4.618473896	LOC106588560	Transcription
DMRcontig15871:26391751	contig15871	26391751	26392000	250	1	2.43E-06	3	1.2	NA	NA
DMRcontig15876:26399101	contig15876	26399101	26399300	200	2	2.94E-10	7	3.5	NA	NA
DMRcontig15922:26464801	contig15922	26464801	26465252	452	1	1.70E-06	22	4.867256637	NA	NA
DMRcontig15989:26561501	contig15989	26561501	26561857	357	2	3.68E-09	7	1.960784314	NA	NA
DMRcontig15998:26574501	contig15998	26574501	26574900	400	1	7.34E-06	7	1.75	NA	NA
DMRcontig16026:26615401	contig16026	26615401	26615700	300	1	5.02E-06	5	1.666666667	NA	NA
DMRcontig16030:26621608	contig16030	26621608	26622000	393	1	3.51E-06	6	1.526717557	NA	NA
DMRcontig16038:26632901	contig16038	26632901	26633398	498	1	1.59E-10	15	3.012048193	NA	NA
DMRcontig16049:26649201	contig16049	26649201	26649846	646	1	4.36E-08	22	3.405572755	NA	NA
DMRcontig16114:26743801	contig16114	26743801	26744000	200	2	1.46E-08	6	3	NA	NA
DMRcontig16116:26746801	contig16116	26746801	26747047	247	1	8.04E-06	2	0.809716599	NA	NA
DMRcontig16142:26783624	contig16142	26783624	26783900	277	1	2.28E-10	20	7.220216606	NA	NA
DMRcontig16218:26896001	contig16218	26896001	26896300	300	1	5.66E-07	15	5	NA	NA
DMRcontig16221:26900301	contig16221	26900301	26900640	340	1	9.54E-06	18	5.294117647	NA	NA
DMRcontig16301:27017434	contig16301	27017434	27017700	267	2	1.59E-07	7	2.621722846	NA	NA
DMRcontig16488:27287413	contig16488	27287413	27287678	266	2	1.87E-07	10	3.759398496	NA	NA
DMRcontig16512:27321843	contig16512	27321843	27322129	287	1	1.10E-06	8	2.787456446	NA	NA
DMRcontig16601:27450601	contig16601	27450601	27450900	300	2	2.13E-07	7	2.333333333	NA	NA
DMRcontig16630:27491601	contig16630	27491601	27491900	300	1	8.28E-06	8	2.666666667	NA	NA
DMRcontig16638:27503501	contig16638	27503501	27503900	400	1	2.10E-06	18	4.5	NA	NA
DMRcontig16701:27595501	contig16701	27595501	27596000	500	1	9.29E-06	9	1.8	NA	NA
DMRcontig16719:27621626	contig16719	27621626	27622000	375	2	1.60E-11	10	2.666666667	NA	NA
DMRcontig16742:27655401	contig16742	27655401	27655800	400	1	1.67E-06	9	2.25	NA	NA
DMRcontig16828:27779701	contig16828	27779701	27779900	200	1	2.56E-06	6	3	NA	NA
DMRcontig17000:28030001	contig17000	28030001	28030200	200	1	8.63E-06	9	4.5	LOC106165041	Unknown
DMRcontig17096:28166201	contig17096	28166201	28166525	325	1	6.86E-06	10	3.076923077	NA	NA
DMRcontig17170:28270801	contig17170	28270801	28271700	900	1	3.69E-06	11	1.222222222	NA	NA
DMRcontig17180:28285033	contig17180	28285033	28285400	368	1	1.40E-08	22	5.97826087	NA	NA
DMRcontig17306:28464336	contig17306	28464336	28464500	165	1	2.32E-07	1	0.606060606	NA	NA
DMRcontig17352:28529401	contig17352	28529401	28529880	480	1	2.15E-06	5	1.041666667	NA	NA
DMRcontig17355:28533712	contig17355	28533712	28534100	389	1	5.60E-06	11	2.827763496	NA	NA
DMRcontig17448:28664748	contig17448	28664748	28664962	215	1	1.22E-06	8	3.720930233	NA	NA
DMRcontig17532:28783347	contig17532	28783347	28783767	421	1	7.59E-06	14	3.325415677	NA	NA
DMRcontig17543:28799001	contig17543	28799001	28799259	259	1	2.65E-07	6	2.316602317	NA	NA
DMRcontig17604:28886260	contig17604	28886260	28886632	373	2	7.51E-09	8	2.144772118	NA	NA
DMRcontig17650:28953301	contig17650	28953301	28953673	373	1	1.43E-08	12	3.217158177	NA	NA
DMRcontig17690:29009615	contig17690	29009615	29009827	213	1	8.28E-08	1	0.469483568	NA	NA
DMRcontig17739:29077444	contig17739	29077444	29077700	257	1	1.88E-07	0	0	NA	NA
DMRcontig17753:29099201	contig17753	29099201	29099400	200	1	1.34E-06	3	1.5	NA	NA
DMRcontig17888:29290134	contig17888	29290134	29290400	267	2	3.85E-07	9	3.370786517	NA	NA
DMRcontig17893:29296901	contig17893	29296901	29297100	200	1	9.03E-07	8	4	NA	NA
DMRcontig17968:29401611	contig17968	29401611	29401800	190	1	4.38E-10	4	2.105263158	NA	NA
DMRcontig17988:29429901	contig17988	29429901	29430100	200	1	4.93E-08	10	5	NA	NA
DMRcontig18015:29468635	contig18015	29468635	29468900	266	1	7.19E-07	14	5.263157895	NA	NA
DMRcontig18047:29512546	contig18047	29512546	29512777	232	1	3.93E-07	7	3.017241379	NA	NA
DMRcontig18129:29628501	contig18129	29628501	29628800	300	1	7.55E-06	7	2.333333333	NA	NA
DMRcontig18150:29657875	contig18150	29657875	29658300	426	1	6.49E-06	15	3.521126761	NA	NA
DMRcontig18177:29695401	contig18177	29695401	29696034	634	4	2.56E-09	23	3.627760252	NA	NA
DMRcontig18196:29723801	contig18196	29723801	29723925	125	1	3.81E-06	2	1.6	NA	NA
DMRcontig18220:29755175	contig18220	29755175	29755385	211	1	5.53E-06	6	2.843601896	NA	NA
DMRcontig18225:29762101	contig18225	29762101	29762377	277	1	1.90E-11	11	3.971119134	NA	NA
DMRcontig18273:29832101	contig18273	29832101	29832600	500	2	4.25E-09	6	1.2	NA	NA
DMRcontig18281:29842924	contig18281	29842924	29843157	234	1	7.23E-06	5	2.136752137	NA	NA
DMRcontig18292:29858701	contig18292	29858701	29858800	100	1	8.54E-06	9	9	NA	NA
DMRcontig18438:30067501	contig18438	30067501	30067800	300	1	1.54E-06	35	11.66666667	NA	NA
DMRcontig18443:30074301	contig18443	30074301	30074552	252	1	2.61E-12	13	5.158730159	NA	NA

DMRcontig18464:30105501	contig18464	30105501	30106400	900	1	3.21E-06	58	6.444444444	NA	NA
DMRcontig18565:30252145	contig18565	30252145	30252700	556	3	8.09E-08	14	2.517985612	NA	NA
DMRcontig18588:30286401	contig18588	30286401	30286818	418	2	3.65E-08	12	2.870813397	NA	NA
DMRcontig18622:30333601	contig18622	30333601	30333900	300	1	5.26E-08	5	1.666666667	NA	NA
DMRcontig18671:30399901	contig18671	30399901	30400296	396	1	2.20E-07	17	4.292929293	NA	NA
DMRcontig18687:30423201	contig18687	30423201	30423500	300	1	4.75E-06	9	3	NA	NA
DMRcontig18713:30466501	contig18713	30466501	30467323	823	2	9.04E-08	28	3.40218712	NA	NA
DMRcontig18737:30505414	contig18737	30505414	30506300	887	6	5.84E-13	58	6.538895152	NA	NA
DMRcontig18751:30529301	contig18751	30529301	30529780	480	1	1.95E-09	7	1.458333333	NA	NA
DMRcontig18781:30575701	contig18781	30575701	30576000	300	2	1.43E-11	2	0.666666667	NA	NA
DMRcontig18782:30577401	contig18782	30577401	30577900	500	1	8.37E-06	20	4	NA	NA
DMRcontig18824:30644361	contig18824	30644361	30644900	540	2	1.29E-08	22	4.074074074	NA	NA
DMRcontig18836:30663101	contig18836	30663101	30663300	200	1	4.91E-06	6	3	NA	NA
DMRcontig18848:30682501	contig18848	30682501	30683500	1000	3	1.67E-08	13	1.3	NA	NA
DMRcontig18872:30720634	contig18872	30720634	30721100	467	1	5.43E-07	28	5.995717345	NA	NA
DMRcontig18892:30751701	contig18892	30751701	30752595	895	2	9.22E-13	36	4.022346369	NA	NA
DMRcontig18897:30759444	contig18897	30759444	30759641	198	1	3.82E-07	0	0	NA	NA
DMRcontig19031:30964005	contig19031	30964005	30964200	196	1	3.94E-08	7	3.571428571	NA	NA
DMRcontig19048:30989914	contig19048	30989914	30990200	287	3	1.96E-07	9	3.135888502	NA	NA
DMRcontig19055:31000901	contig19055	31000901	31001068	168	1	9.56E-07	2	1.19047619	NA	NA
DMRcontig19089:31050838	contig19089	31050838	31051300	463	1	3.53E-07	7	1.51187905	NA	NA
DMRcontig19235:31267301	contig19235	31267301	31267500	200	1	5.36E-08	11	5.5	NA	NA
DMRcontig19240:31275101	contig19240	31275101	31275449	349	1	6.78E-07	8	2.29226361	NA	NA
DMRcontig19248:31287715	contig19248	31287715	31288200	486	2	1.60E-10	14	2.880658436	NA	NA
DMRcontig19262:31308253	contig19262	31308253	31308500	248	1	5.73E-08	5	2.016129032	NA	NA
DMRcontig19294:31358801	contig19294	31358801	31359000	200	1	2.57E-06	7	3.5	EMWEY_00012150;cno	Development;Unknown
DMRcontig19369:31470101	contig19369	31470101	31470600	500	2	5.79E-09	22	4.4	NA	NA
DMRcontig19370:31471801	contig19370	31471801	31472300	500	2	1.81E-06	14	2.8	NA	NA
DMRcontig19376:31480443	contig19376	31480443	31480600	158	1	7.18E-07	8	5.063291139	NA	NA
DMRcontig19388:31498901	contig19388	31498901	31499499	599	2	6.62E-07	39	6.510851419	NA	NA
DMRcontig19430:31559001	contig19430	31559001	31559200	200	1	2.11E-07	13	6.5	NA	NA
DMRcontig19453:31593301	contig19453	31593301	31594295	995	2	5.61E-07	67	6.733668342	NA	NA
DMRcontig19520:31692901	contig19520	31692901	31693100	200	1	1.62E-09	6	3	NA	NA
DMRcontig19556:31743801	contig19556	31743801	31744000	200	1	3.82E-06	13	6.5	NA	NA
DMRcontig19590:31792701	contig19590	31792701	31792973	273	1	9.23E-06	17	6.227106227	NA	NA
DMRcontig19631:31851601	contig19631	31851601	31851800	200	2	1.50E-10	5	2.5	NA	NA
DMRcontig19659:31892501	contig19659	31892501	31892770	270	2	4.06E-06	7	2.592592593	NA	NA
DMRcontig19681:31924301	contig19681	31924301	31924500	200	1	2.81E-07	16	8	NA	NA
DMRcontig19768:32050601	contig19768	32050601	32050686	86	1	7.40E-06	7	8.139534884	Trnac-gca	Translation
DMRcontig19783:32072001	contig19783	32072001	32072400	400	1	8.46E-06	18	4.5	NA	NA
DMRcontig19795:32088901	contig19795	32088901	32089200	300	2	8.36E-10	20	6.666666667	NA	NA
DMRcontig19821:32128483	contig19821	32128483	32128700	218	1	7.06E-08	7	3.211009174	NA	NA
DMRcontig19935:32295565	contig19935	32295565	32295852	288	3	2.08E-07	8	2.777777778	NA	NA
DMRcontig20060:32484301	contig20060	32484301	32484800	500	1	9.69E-08	15	3	NA	NA
DMRcontig20061:32485894	contig20061	32485894	32486300	407	1	5.52E-06	0	0	NA	NA
DMRcontig20232:32757501	contig20232	32757501	32757700	200	1	4.10E-06	3	1.5	NA	NA
DMRcontig20248:32781652	contig20248	32781652	32782428	777	5	8.40E-09	43	5.534105534	NA	NA
DMRcontig20335:32911901	contig20335	32911901	32912300	400	1	5.98E-08	29	7.25	NA	NA
DMRcontig20358:32944756	contig20358	32944756	32945200	445	4	1.00E-08	22	4.943820225	CNPV234	Signaling
DMRcontig20557:33219601	contig20557	33219601	33219891	291	1	4.06E-06	3	1.030927835	NA	NA
DMRcontig20610:33289320	contig20610	33289320	33289500	181	2	9.18E-65	0	0	NA	NA
DMRcontig20735:33448801	contig20735	33448801	33449230	430	1	1.14E-06	22	5.11627907	NA	NA
DMRcontig20887:33643901	contig20887	33643901	33644100	200	1	2.61E-10	0	0	NA	NA
DMRcontig20904:33664753	contig20904	33664753	33665100	348	2	9.61E-09	14	4.022988506	NA	NA
DMRcontig20987:33771624	contig20987	33771624	33772376	753	1	1.98E-11	18	2.390438247	NA	NA
DMRcontig21167:33998901	contig21167	33998901	33999356	456	2	7.74E-08	27	5.921052632	NA	NA
DMRcontig21206:34056179	contig21206	34056179	34056300	122	1	7.22E-06	7	5.737704918	NA	NA
DMRcontig21247:34110474	contig21247	34110474	34110858	385	1	9.70E-06	9	2.337662338	NA	NA
DMRcontig21790:34707965	contig21790	34707965	34708400	436	1	1.77E-06	7	1.605504587	NA	NA
DMRcontig22188:35163038	contig22188	35163038	35163400	363	1	3.14E-06	2	0.550964187	NA	NA
DMRcontig22912:36010301	contig22912	36010301	36010700	400	1	9.02E-06	3	0.75	NA	NA
DMRcontig23099:36230501	contig23099	36230501	36230656	156	2	2.26E-07	12	7.692307692	NA	NA
DMRcontig23683:36906892	contig23683	36906892	36907100	209	1	5.57E-10	2	0.956937799	NA	NA
DMRcontig24120:37456550	contig24120	37456550	37457135	586	1	5.62E-06	15	2.559726962	NA	NA
DMRcontig24132:37475401	contig24132	37475401	37475700	300	2	7.05E-08	7	2.333333333	NA	NA
DMRcontig24157:37513301	contig24157	37513301	37513700	400	2	3.89E-10	23	5.75	NA	NA

DMRcontig24178:37544301	contig24178	37544301	37544618	318	2	5.21E-11	11	3.459119497	NA	NA
DMRcontig24211:37592801	contig24211	37592801	37593200	400	2	4.56E-12	35	8.75	NA	NA
DMRcontig24216:37600056	contig24216	37600056	37600400	345	2	1.96E-10	15	4.347826087	NA	NA
DMRcontig24370:37820915	contig24370	37820915	37821100	186	1	2.42E-06	7	3.76344086	NA	NA
DMRcontig24473:37961205	contig24473	37961205	37961640	436	3	8.92E-11	11	2.52293578	NA	NA
DMRcontig24523:38026801	contig24523	38026801	38027100	300	1	2.90E-06	15	5	NA	NA
DMRcontig24604:38127222	contig24604	38127222	38127700	479	1	5.07E-06	18	3.75782881	NA	NA
DMRcontig24851:38443701	contig24851	38443701	38444028	328	1	1.63E-06	2	0.609756098	NA	NA
DMRcontig24989:38620501	contig24989	38620501	38620984	484	1	3.63E-06	30	6.198347107	NA	NA
DMRcontig24993:38626601	contig24993	38626601	38626900	300	1	8.15E-09	18	6	LOC102794729;rpl23a	Translation
DMRcontig25919:39674931	contig25919	39674931	39675372	442	2	2.24E-13	40	9.049773756	NA	NA
DMRcontig26010:39781719	contig26010	39781719	39782198	480	1	9.07E-06	17	3.541666667	NA	NA
DMRcontig26094:39882101	contig26094	39882101	39882554	454	2	1.39E-06	9	1.982378855	NA	NA
DMRcontig26188:39996052	contig26188	39996052	39996382	331	1	1.25E-06	9	2.719033233	NA	NA
DMRcontig26323:40155350	contig26323	40155350	40155730	381	2	3.27E-08	10	2.624671916	NA	NA
DMRcontig26379:40222001	contig26379	40222001	40222238	238	1	2.47E-10	2	0.840336134	NA	NA
DMRcontig26614:40496401	contig26614	40496401	40496587	187	1	1.24E-07	22	11.76470588	NA	NA
DMRcontig26773:40681801	contig26773	40681801	40681910	110	2	2.22E-11	7	6.363636364	NA	NA
DMRcontig27026:40981501	contig27026	40981501	40981640	140	2	5.86E-09	0	0	NA	NA
DMRcontig27397:41449201	contig27397	41449201	41449400	200	1	9.31E-06	2	1	NA	NA
DMRcontig27575:41727001	contig27575	41727001	41727441	441	2	1.27E-06	13	2.947845805	NA	NA
DMRcontig27649:41836006	contig27649	41836006	41836400	395	1	1.13E-06	33	8.35443038	NA	NA
DMRcontig27654:41843201	contig27654	41843201	41843600	400	1	2.69E-07	10	2.5	NA	NA
DMRcontig27701:41913901	contig27701	41913901	41914355	455	2	6.14E-08	24	5.274725275	NA	NA
DMRcontig27707:41922801	contig27707	41922801	41923092	292	1	1.51E-06	1	0.342465753	NA	NA
DMRcontig27758:41995401	contig27758	41995401	41995600	200	1	7.45E-06	11	5.5	NA	NA
DMRcontig27759:41996802	contig27759	41996802	41997200	399	1	3.24E-07	7	1.754385965	NA	NA
DMRcontig27777:42022101	contig27777	42022101	42022400	300	1	4.41E-06	26	8.666666667	NA	NA
DMRcontig27823:42087694	contig27823	42087694	42088100	407	1	8.84E-06	8	1.965601966	NA	NA
DMRcontig27828:42095135	contig27828	42095135	42095700	566	1	3.34E-07	9	1.590106007	NA	NA
DMRcontig27883:42172378	contig27883	42172378	42172800	423	3	1.45E-14	21	4.964539007	NA	NA
DMRcontig27900:42196811	contig27900	42196811	42197186	376	1	1.04E-06	13	3.457446809	NA	NA
DMRcontig27964:42284101	contig27964	42284101	42284400	300	1	1.26E-06	21	7	NA	NA
DMRcontig28063:42414144	contig28063	42414144	42414400	257	1	9.41E-07	3	1.167315175	NA	NA
DMRcontig28086:42442715	contig28086	42442715	42442775	61	1	2.88E-25	1	1.639344262	NA	NA
DMRcontig28204:42599701	contig28204	42599701	42600089	389	2	2.85E-12	5	1.285347044	NA	NA
DMRcontig28261:42676801	contig28261	42676801	42677300	500	3	8.33E-10	7	1.4	NA	NA
DMRcontig28285:42710101	contig28285	42710101	42710300	200	2	2.53E-10	1	0.5	NA	NA
DMRcontig28659:43216601	contig28659	43216601	43217100	500	3	3.34E-10	7	1.4	NA	NA
DMRcontig28672:43236001	contig28672	43236001	43236400	400	1	4.12E-09	6	1.5	NA	NA
DMRcontig29161:43784552	contig29161	43784552	43785000	449	1	1.30E-06	24	5.345211581	NA	NA
DMRcontig29286:43928527	contig29286	43928527	43928727	201	2	1.14E-09	12	5.970149254	NA	NA
DMRcontig29322:43968981	contig29322	43968981	43969300	320	1	1.66E-07	8	2.5	NA	NA
DMRcontig29779:44504338	contig29779	44504338	44504545	208	1	2.54E-06	6	2.884615385	NA	NA
DMRcontig29820:44553411	contig29820	44553411	44553859	449	5	2.16E-11	24	5.345211581	NA	NA
DMRcontig30021:44789962	contig30021	44789962	44790100	139	2	3.16E-10	5	3.597122302	NA	NA
DMRcontig30070:44847477	contig30070	44847477	44847631	155	1	8.20E-09	3	1.935483871	NA	NA
DMRcontig30129:44919201	contig30129	44919201	44919600	400	1	1.41E-07	23	5.75	NA	NA
DMRcontig30241:45053507	contig30241	45053507	45053618	112	1	2.01E-07	4	3.571428571	NA	NA
DMRcontig30440:45292001	contig30440	45292001	45292200	200	1	3.89E-07	2	1	NA	NA
DMRcontig30540:45412001	contig30540	45412001	45412105	105	1	8.28E-07	0	0	NA	NA
DMRcontig30582:45461244	contig30582	45461244	45461500	257	2	1.27E-06	4	1.556420233	NA	NA
DMRcontig30725:45633001	contig30725	45633001	45633352	352	2	1.47E-06	21	5.965909091	NA	NA
DMRcontig30748:45661185	contig30748	45661185	45661277	93	1	1.18E-08	3	3.225806452	NA	NA
DMRcontig30771:45689401	contig30771	45689401	45689500	100	1	3.82E-06	4	4	NA	NA
DMRcontig30779:45698908	contig30779	45698908	45699240	333	2	1.54E-08	10	3.003003003	NA	NA
DMRcontig30826:45755201	contig30826	45755201	45755585	385	2	4.75E-07	6	1.558441558	NA	NA
DMRcontig30866:45803039	contig30866	45803039	45803257	219	1	8.73E-11	6	2.739726027	NA	NA
DMRcontig31048:46015206	contig31048	46015206	46015500	295	2	5.38E-16	36	12.20338983	NA	NA
DMRcontig31132:46112801	contig31132	46112801	46113089	289	1	8.14E-06	4	1.384083045	NA	NA
DMRcontig31168:46172301	contig31168	46172301	46172800	500	4	8.90E-08	23	4.6	NA	NA
DMRcontig31170:46175401	contig31170	46175401	46175600	200	2	8.49E-09	6	3	NA	NA
DMRcontig31174:46182401	contig31174	46182401	46182700	300	1	1.43E-06	24	8	NA	NA
DMRcontig31193:46212101	contig31193	46212101	46212400	300	1	7.48E-08	5	1.666666667	NA	NA
DMRcontig31255:46307201	contig31255	46307201	46307400	200	1	1.11E-06	13	6.5	NA	NA
DMRcontig31465:46608801	contig31465	46608801	46609100	300	1	2.91E-09	4	1.333333333	NA	NA

DMRcontig31471:46617135	contig31471	46617135	46617671	537	1	7.08E-06	7	1.303538175	NA	NA
DMRcontig31516:46679801	contig31516	46679801	46680146	346	1	5.02E-07	6	1.734104046	NA	NA
DMRcontig31525:46691727	contig31525	46691727	46692000	274	2	4.68E-09	6	2.189781022	NA	NA
DMRcontig31594:46784533	contig31594	46784533	46784700	168	2	1.13E-07	9	5.357142857	NA	NA
DMRcontig31612:46807101	contig31612	46807101	46807500	400	1	7.14E-06	14	3.5	NA	NA
DMRcontig32047:47352701	contig32047	47352701	47352900	200	1	4.19E-07	15	7.5	NA	NA
DMRcontig32074:47385113	contig32074	47385113	47385199	87	1	3.38E-06	0	0	NA	NA
DMRcontig32083:47395601	contig32083	47395601	47395700	100	1	5.33E-06	13	13	NA	NA
DMRcontig32090:47404201	contig32090	47404201	47404500	300	1	9.36E-10	11	3.666666667	HPODL_04312	Unknown
DMRcontig32100:47417651	contig32100	47417651	47417869	219	1	1.25E-06	2	0.913242009	NA	NA
DMRcontig32635:48023201	contig32635	48023201	48023593	393	1	4.95E-07	17	4.325699746	NA	NA
DMRcontig32670:48063601	contig32670	48063601	48063829	229	2	7.02E-12	11	4.80349345	NA	NA
DMRcontig32917:48343382	contig32917	48343382	48343495	114	1	3.87E-07	2	1.754385965	NA	NA
DMRcontig33026:48467215	contig33026	48467215	48467499	285	1	1.33E-07	16	5.614035088	NA	NA
DMRcontig33157:48617741	contig33157	48617741	48618000	260	1	1.54E-08	6	2.307692308	NA	NA
DMRcontig33273:48750274	contig33273	48750274	48750651	378	3	1.72E-12	11	2.91005291	NA	NA
DMRcontig33365:48856701	contig33365	48856701	48857100	400	2	1.90E-06	17	4.25	NA	NA
DMRcontig33515:49030401	contig33515	49030401	49030881	481	2	9.63E-11	26	5.405405405	NA	NA
DMRcontig33620:49149101	contig33620	49149101	49149299	199	1	5.74E-06	8	4.020100503	NA	NA
DMRcontig34014:49605342	contig34014	49605342	49605667	326	2	2.31E-11	5	1.533742331	NA	NA
DMRcontig34074:49674170	contig34074	49674170	49674400	231	1	2.05E-06	3	1.298701299	NA	NA
DMRcontig34132:49744201	contig34132	49744201	49744425	225	1	4.78E-07	10	4.444444444	NA	NA
DMRcontig34178:49797801	contig34178	49797801	49798120	320	2	1.46E-06	13	4.0625	NA	NA
DMRcontig34205:49830101	contig34205	49830101	49830380	280	1	2.32E-07	7	2.5	NA	NA
DMRcontig34217:49844601	contig34217	49844601	49844871	271	2	1.63E-07	16	5.904059041	NA	NA
DMRcontig34516:50187443	contig34516	50187443	50187700	258	1	1.46E-08	13	5.03875969	NA	NA
DMRcontig34798:50514121	contig34798	50514121	50514349	229	1	1.93E-10	9	3.930131004	NA	NA
DMRcontig35171:51024801	contig35171	51024801	51025200	400	2	6.24E-07	5	1.25	NA	NA
DMRcontig35321:51263501	contig35321	51263501	51264000	500	4	2.30E-16	20	4	NA	NA
DMRcontig35334:51282701	contig35334	51282701	51283100	400	2	5.74E-09	19	4.75	NA	NA
DMRcontig35335:51284801	contig35335	51284801	51285100	300	1	5.97E-06	12	4	NA	NA
DMRcontig35410:51403039	contig35410	51403039	51403300	262	2	1.98E-06	3	1.145038168	NA	NA
DMRcontig35514:51561733	contig35514	51561733	51562100	368	1	2.94E-06	8	2.173913043	NA	NA
DMRcontig35537:51596501	contig35537	51596501	51597100	600	1	1.92E-08	5	0.833333333	NA	NA
DMRcontig35580:51662801	contig35580	51662801	51663262	462	2	5.84E-10	8	1.731601732	NA	NA
DMRcontig35659:51784701	contig35659	51784701	51785000	300	1	8.27E-06	8	2.666666667	NA	NA
DMRcontig35712:51865465	contig35712	51865465	51865700	236	1	1.50E-07	13	5.508474576	NA	NA
DMRcontig35719:51877321	contig35719	51877321	51877500	180	1	8.03E-07	1	0.555555556	NA	NA
DMRcontig35732:51896501	contig35732	51896501	51896831	331	1	3.16E-06	6	1.812688822	NA	NA
DMRcontig35746:51917901	contig35746	51917901	51918492	592	3	8.46E-08	16	2.702702703	NA	NA
DMRcontig35795:51991851	contig35795	51991851	51992418	568	5	2.71E-12	25	4.401408451	NA	NA
DMRcontig35834:52050401	contig35834	52050401	52050610	210	1	2.06E-07	16	7.619047619	NA	NA
DMRcontig35857:52084658	contig35857	52084658	52085100	443	3	9.35E-14	28	6.320541761	NA	NA
DMRcontig35887:52128901	contig35887	52128901	52129398	498	1	1.46E-07	18	3.614457831	NA	NA
DMRcontig35941:52210041	contig35941	52210041	52210400	360	1	3.55E-15	18	5	NA	NA
DMRcontig35995:52290285	contig35995	52290285	52290700	416	1	4.57E-08	6	1.442307692	NA	NA
DMRcontig36074:52409901	contig36074	52409901	52410200	300	2	2.73E-07	16	5.333333333	NA	NA
DMRcontig36117:52473301	contig36117	52473301	52473600	300	1	3.58E-06	11	3.666666667	NA	NA
DMRcontig36144:52513601	contig36144	52513601	52513900	300	2	1.35E-08	11	3.666666667	NA	NA
DMRcontig36183:52572397	contig36183	52572397	52572500	104	1	9.80E-06	5	4.807692308	NA	NA
DMRcontig36194:52588234	contig36194	52588234	52588700	467	1	8.67E-07	31	6.638115632	NA	NA
DMRcontig36195:52589717	contig36195	52589717	52590100	384	1	4.90E-06	13	3.385416667	NA	NA
DMRcontig36246:52665101	contig36246	52665101	52665500	400	2	5.08E-07	22	5.5	NA	NA
DMRcontig36284:52722901	contig36284	52722901	52723100	200	2	5.80E-08	0	0	NA	NA
DMRcontig36358:52829914	contig36358	52829914	52830200	287	2	3.80E-08	5	1.742160279	NA	NA
DMRcontig36371:52849001	contig36371	52849001	52849448	448	3	8.45E-13	32	7.142857143	NA	NA
DMRcontig36539:53099201	contig36539	53099201	53099334	134	1	5.52E-06	9	6.71641791	NA	NA
DMRcontig36581:53161172	contig36581	53161172	53161400	229	2	1.44E-10	4	1.746724891	NA	NA
DMRcontig36807:53500101	contig36807	53500101	53500500	400	1	1.12E-07	24	6	NA	NA
DMRcontig36904:53641901	contig36904	53641901	53642396	496	2	1.17E-06	12	2.419354839	NA	NA
DMRcontig36967:53734301	contig36967	53734301	53734558	258	2	6.65E-14	8	3.100775194	NA	NA
DMRcontig37023:53817301	contig37023	53817301	53817781	481	1	1.27E-06	19	3.95010395	NA	NA
DMRcontig37054:53862153	contig37054	53862153	53862600	448	3	2.86E-11	20	4.464285714	NA	NA
DMRcontig37105:53936701	contig37105	53936701	53936900	200	1	3.22E-07	4	2	NA	NA
DMRcontig37194:54069801	contig37194	54069801	54070100	300	1	7.57E-08	7	2.333333333	NA	NA
DMRcontig37196:54072701	contig37196	54072701	54073100	400	1	6.72E-06	12	3	NA	NA

DMRcontig37266:54176801	contig37266	54176801	54177100	300	2	7.16E-11	8	2.666666667	NA	NA
DMRcontig37275:54189766	contig37275	54189766	54190200	435	3	2.05E-13	26	5.977011494	NA	NA
DMRcontig37293:54217210	contig37293	54217210	54217400	191	2	2.14E-08	1	0.523560209	NA	NA
DMRcontig37303:54231447	contig37303	54231447	54232200	754	2	2.67E-06	36	4.774535809	NA	NA
DMRcontig37306:54236174	contig37306	54236174	54236700	527	1	1.75E-07	16	3.036053131	NA	NA
DMRcontig37308:54239501	contig37308	54239501	54239955	455	1	3.27E-06	16	3.516483516	NA	NA
DMRcontig37315:54249814	contig37315	54249814	54250200	387	1	5.07E-08	18	4.651162791	NA	NA
DMRcontig37325:54264167	contig37325	54264167	54264500	334	1	9.31E-06	8	2.395209581	NA	NA
DMRcontig37417:54397501	contig37417	54397501	54397780	280	2	4.70E-06	7	2.5	NA	NA
DMRcontig37481:54490801	contig37481	54490801	54491193	393	2	1.70E-08	12	3.053435115	NA	NA
DMRcontig37513:54539801	contig37513	54539801	54540100	300	1	2.81E-07	9	3	NA	NA
DMRcontig37596:54661301	contig37596	54661301	54661835	535	1	1.80E-06	30	5.607476636	LOC103188862	Metabolism
DMRcontig37626:54704401	contig37626	54704401	54705100	700	2	1.11E-10	19	2.714285714	NA	NA
DMRcontig37655:54746801	contig37655	54746801	54746900	100	1	3.84E-06	4	4	NA	NA
DMRcontig37702:54815301	contig37702	54815301	54815600	300	1	6.28E-06	12	4	NA	NA
DMRcontig37710:54827201	contig37710	54827201	54827483	283	1	1.50E-08	11	3.886925795	NA	NA
DMRcontig37763:54904966	contig37763	54904966	54905300	335	2	2.83E-08	18	5.373134328	NA	NA
DMRcontig37765:54908101	contig37765	54908101	54908300	200	1	8.40E-06	9	4.5	NA	NA
DMRcontig37846:55029105	contig37846	55029105	55029400	296	1	4.98E-07	10	3.378378378	NA	NA
DMRcontig37954:55187878	contig37954	55187878	55188200	323	2	8.37E-07	5	1.547987616	NA	NA
DMRcontig37979:55225301	contig37979	55225301	55225599	299	1	5.86E-08	3	1.003344482	NA	NA
DMRcontig38028:55296701	contig38028	55296701	55296900	200	1	9.86E-06	3	1.5	NA	NA
DMRcontig38043:55318701	contig38043	55318701	55318900	200	1	1.28E-06	5	2.5	NA	NA
DMRcontig38081:55374739	contig38081	55374739	55375200	462	1	6.51E-07	9	1.948051948	NA	NA
DMRcontig38094:55393537	contig38094	55393537	55393800	264	1	5.12E-06	7	2.651515152	NA	NA
DMRcontig38162:55494177	contig38162	55494177	55494636	460	1	6.26E-06	27	5.869565217	NA	NA
DMRcontig38202:55551528	contig38202	55551528	55551800	273	1	7.74E-06	6	2.197802198	NA	NA
DMRcontig38222:55581571	contig38222	55581571	55581900	330	1	2.14E-06	8	2.424242424	NA	NA
DMRcontig38282:55669473	contig38282	55669473	55669700	228	1	3.31E-06	7	3.070175439	NA	NA
DMRcontig38325:55731841	contig38325	55731841	55732100	260	1	5.23E-06	11	4.230769231	NA	NA
DMRcontig38513:56005101	contig38513	56005101	56005780	680	2	4.78E-07	29	4.264705882	NA	NA
DMRcontig38559:56072134	contig38559	56072134	56072500	367	1	3.93E-06	6	1.634877384	NA	NA
DMRcontig38563:56077912	contig38563	56077912	56078200	289	1	2.47E-06	7	2.422145329	NA	NA
DMRcontig38564:56079271	contig38564	56079271	56079600	330	2	9.90E-08	14	4.242424242	NA	NA
DMRcontig38674:56237101	contig38674	56237101	56237583	483	4	5.94E-11	31	6.418219462	NA	NA
DMRcontig38705:56282328	contig38705	56282328	56282600	273	2	7.90E-10	12	4.395604396	NA	NA
DMRcontig38751:56349024	contig38751	56349024	56349500	477	1	3.48E-07	23	4.821802935	NA	NA
DMRcontig38790:56405801	contig38790	56405801	56405988	188	1	5.49E-06	6	3.191489362	NA	NA
DMRcontig38818:56446201	contig38818	56446201	56446649	449	1	2.46E-06	8	1.781737194	NA	NA
DMRcontig38856:56501501	contig38856	56501501	56501700	200	2	9.45E-12	7	3.5	NA	NA
DMRcontig38871:56523201	contig38871	56523201	56523600	400	2	8.21E-07	10	2.5	NA	NA
DMRcontig38896:56559601	contig38896	56559601	56559900	300	1	1.48E-07	7	2.333333333	NA	NA
DMRcontig38997:56706101	contig38997	56706101	56706400	300	1	1.39E-06	7	2.333333333	NA	NA
DMRcontig39026:56748201	contig39026	56748201	56748400	200	1	5.87E-06	9	4.5	NA	NA
DMRcontig39048:56780063	contig39048	56780063	56780400	338	1	2.80E-07	3	0.887573964	NA	NA
DMRcontig39058:56794901	contig39058	56794901	56795293	393	1	7.29E-06	10	2.544529262	NA	NA
DMRcontig39079:56824545	contig39079	56824545	56825000	456	2	1.27E-09	23	5.043859649	NA	NA
DMRcontig39100:56855204	contig39100	56855204	56855894	691	1	3.47E-06	16	2.315484805	NA	NA
DMRcontig39121:56885615	contig39121	56885615	56886000	386	2	1.23E-08	21	5.440414508	NA	NA
DMRcontig39313:57160301	contig39313	57160301	57160592	292	1	3.41E-07	17	5.821917808	NA	NA
DMRcontig39319:57168601	contig39319	57168601	57168800	200	2	1.29E-08	10	5	NA	NA
DMRcontig39402:57287519	contig39402	57287519	57287887	369	2	6.99E-08	25	6.775067751	NA	NA
DMRcontig39457:57368150	contig39457	57368150	57368400	251	1	6.57E-07	12	4.780876494	NA	NA
DMRcontig39458:57369801	contig39458	57369801	57370000	200	1	2.10E-06	3	1.5	NA	NA
DMRcontig39573:57535101	contig39573	57535101	57535400	300	1	6.99E-07	7	2.333333333	NA	NA
DMRcontig39594:57565200	contig39594	57565200	57565600	401	1	1.54E-06	7	1.74563591	NA	NA
DMRcontig39733:57764501	contig39733	57764501	57764774	274	1	2.35E-06	5	1.824817518	APOB-ICR;APOB3'MAR;LOC106560211;APOB	Binding Protein;Unknown
DMRcontig39819:57886926	contig39819	57886926	57887396	471	1	3.50E-06	17	3.609341826	NA	NA
DMRcontig39871:57961039	contig39871	57961039	57961200	162	1	4.77E-09	4	2.469135802	NA	NA
DMRcontig39965:58096501	contig39965	58096501	58096800	300	1	2.09E-10	11	3.666666667	NA	NA
DMRcontig40039:58203401	contig40039	58203401	58204045	645	1	2.58E-07	9	1.395348837	NA	NA
DMRcontig40252:58506401	contig40252	58506401	58506519	119	1	1.35E-07	1	0.840336134	si:dkeyp-61b2.1	Unknown
DMRcontig40257:58513270	contig40257	58513270	58513600	331	2	2.39E-10	9	2.719033233	NA	NA
DMRcontig40301:58576701	contig40301	58576701	58576982	282	1	1.28E-06	12	4.255319149	LOC101849745;ATG3	Protease

DMRcontig40317:58599501	contig40317	58599501	58599700	200	1	7.46E-06	9	4.5	NA	NA
DMRcontig40390:58702963	contig40390	58702963	58703200	238	2	2.13E-07	2	0.840336134	NA	NA
DMRcontig40436:58768601	contig40436	58768601	58769084	484	3	3.99E-20	14	2.892561983	NA	NA
DMRcontig40534:58906401	contig40534	58906401	58906600	200	1	8.42E-06	10	5	NA	NA
DMRcontig40568:58954118	contig40568	58954118	58954300	183	1	2.29E-06	8	4.371584699	NA	NA
DMRcontig40588:58982859	contig40588	58982859	58983100	242	2	1.81E-16	13	5.371900826	NA	NA
DMRcontig40592:58988330	contig40592	58988330	58988600	271	2	7.14E-11	20	7.380073801	NA	NA
DMRcontig40641:59057001	contig40641	59057001	59057200	200	1	4.61E-07	3	1.5	NA	NA
DMRcontig40783:59256301	contig40783	59256301	59256600	300	2	8.20E-11	12	4	NA	NA
DMRcontig40827:59317816	contig40827	59317816	59318200	385	3	5.09E-09	13	3.376623377	NA	NA
DMRcontig40853:59354581	contig40853	59354581	59354900	320	1	3.99E-07	8	2.5	NA	NA
DMRcontig40864:59370317	contig40864	59370317	59370700	384	1	4.82E-07	28	7.291666667	NA	NA
DMRcontig40868:59376201	contig40868	59376201	59376565	365	1	4.32E-06	17	4.657534247	NA	NA
DMRcontig41002:59566101	contig41002	59566101	59566400	300	2	3.42E-07	28	9.333333333	NA	NA
DMRcontig41055:59639601	contig41055	59639601	59639800	200	1	2.01E-06	5	2.5	NA	NA
DMRcontig41145:59766238	contig41145	59766238	59766460	223	1	2.33E-06	4	1.793721973	LOC103030616;sfpq	Receptor;Translation
DMRcontig41211:59859562	contig41211	59859562	59859800	239	1	9.13E-06	6	2.510460251	NA	NA
DMRcontig41212:59861101	contig41212	59861101	59861246	146	1	2.56E-06	11	7.534246575	NA	NA
DMRcontig41364:60074457	contig41364	60074457	60074700	244	2	6.76E-09	12	4.918032787	NA	NA
DMRcontig41472:60226601	contig41472	60226601	60226800	200	1	7.37E-06	8	4	NA	NA
DMRcontig41526:60301001	contig41526	60301001	60301300	300	2	8.26E-09	4	1.333333333	NA	NA
DMRcontig41646:60466701	contig41646	60466701	60466979	279	1	8.79E-07	2	0.716845878	NA	NA
DMRcontig41731:60584201	contig41731	60584201	60584300	100	1	4.96E-06	4	4	NA	NA
DMRcontig41778:60649227	contig41778	60649227	60649589	363	1	1.46E-06	18	4.958677686	NA	NA
DMRcontig41857:60760201	contig41857	60760201	60760600	400	2	3.36E-08	11	2.75	NA	NA
DMRcontig41902:60822387	contig41902	60822387	60822700	314	2	7.83E-13	22	7.006369427	NA	NA
DMRcontig41983:60935501	contig41983	60935501	60935700	200	1	3.82E-06	5	2.5	NA	NA
DMRcontig42079:61070301	contig42079	61070301	61070496	196	1	2.24E-06	4	2.040816327	NA	NA
DMRcontig42131:61143474	contig42131	61143474	61143900	427	1	6.68E-07	25	5.854800937	NA	NA
DMRcontig42135:61149102	contig42135	61149102	61149456	355	1	5.54E-06	15	4.225352113	NA	NA
DMRcontig42158:61180822	contig42158	61180822	61181167	346	1	6.99E-08	14	4.046242775	NA	NA
DMRcontig42160:61183301	contig42160	61183301	61183600	300	1	6.67E-06	8	2.666666667	NA	NA
DMRcontig42163:61187147	contig42163	61187147	61187446	300	1	5.10E-06	5	1.666666667	NA	NA
DMRcontig42295:61371286	contig42295	61371286	61371500	215	1	8.66E-06	15	6.976744186	NA	NA
DMRcontig42322:61408050	contig42322	61408050	61408500	451	1	1.43E-06	10	2.2172949	NA	NA
DMRcontig42490:61642904	contig42490	61642904	61643146	243	1	7.06E-07	17	6.995884774	NA	NA
DMRcontig42522:61686701	contig42522	61686701	61687185	485	5	8.49E-20	28	5.773195876	IscW_ISCW023383;sox2	Development
DMRcontig42752:62003201	contig42752	62003201	62003800	600	1	1.71E-06	20	3.333333333	NA	NA
DMRcontig42783:62046301	contig42783	62046301	62046552	252	2	1.78E-07	11	4.365079365	NA	NA
DMRcontig42799:62068945	contig42799	62068945	62069100	156	1	4.55E-06	1	0.641025641	NA	NA
DMRcontig42866:62159767	contig42866	62159767	62160000	234	1	1.41E-06	3	1.282051282	NA	NA
DMRcontig42872:62168075	contig42872	62168075	62168200	126	1	3.32E-06	2	1.587301587	LOC106473850	Signaling
DMRcontig43037:62396434	contig43037	62396434	62396800	367	2	1.67E-13	5	1.36239782	NA	NA
DMRcontig43062:62429801	contig43062	62429801	62430169	369	1	4.02E-06	3	0.81300813	NA	NA
DMRcontig43100:62482335	contig43100	62482335	62482755	421	3	2.59E-06	19	4.513064133	NA	NA
DMRcontig43151:62551701	contig43151	62551701	62552171	471	2	3.14E-09	21	4.458598726	NA	NA
DMRcontig43182:62594101	contig43182	62594101	62594988	888	3	6.69E-11	51	5.743243243	NA	NA
DMRcontig43224:62652407	contig43224	62652407	62652916	510	1	3.68E-09	18	3.529411765	NA	NA
DMRcontig43275:62722845	contig43275	62722845	62723100	256	1	1.79E-07	5	1.953125	NA	NA
DMRcontig43310:62770184	contig43310	62770184	62770500	317	2	8.66E-09	5	1.577287066	NA	NA
DMRcontig43329:62795758	contig43329	62795758	62796149	392	3	8.25E-09	9	2.295918367	NA	NA
DMRcontig43373:62855544	contig43373	62855544	62856100	557	1	1.40E-07	36	6.463195691	NA	NA
DMRcontig43442:62951501	contig43442	62951501	62951699	199	1	3.32E-06	3	1.507537688	NA	NA
DMRcontig43454:62968260	contig43454	62968260	62968500	241	1	2.20E-06	8	3.319502075	NA	NA
DMRcontig43500:63031037	contig43500	63031037	63031300	264	1	2.15E-06	5	1.893939394	NA	NA
DMRcontig43522:63061107	contig43522	63061107	63061385	279	1	9.59E-07	17	6.093189964	NA	NA
DMRcontig43545:63091601	contig43545	63091601	63092095	495	1	9.35E-07	21	4.242424242	NA	NA
DMRcontig43546:63093101	contig43546	63093101	63093300	200	2	1.93E-08	5	2.5	NA	NA
DMRcontig43558:63109486	contig43558	63109486	63109800	315	1	3.70E-06	6	1.904761905	NA	NA
DMRcontig43648:63231915	contig43648	63231915	63232162	248	3	3.97E-10	6	2.419354839	NA	NA
DMRcontig43685:63283201	contig43685	63283201	63283560	360	1	3.68E-06	10	2.777777778	NA	NA
DMRcontig43699:63302901	contig43699	63302901	63303300	400	1	3.04E-06	18	4.5	NA	NA
DMRcontig43727:63342406	contig43727	63342406	63342600	195	1	4.73E-06	11	5.641025641	NA	NA
DMRcontig43881:63552401	contig43881	63552401	63552586	186	2	8.61E-07	6	3.225806452	NA	NA
DMRcontig43884:63556222	contig43884	63556222	63556400	179	1	2.39E-06	7	3.910614525	NA	NA
DMRcontig43890:63564638	contig43890	63564638	63564900	263	1	8.10E-06	6	2.281368821	NA	NA

DMRcontig43906:63586034	contig43906	63586034	63586200	167	1	1.53E-07	1	0.598802395	NA	NA
DMRcontig43917:63600715	contig43917	63600715	63600900	186	1	5.56E-11	11	5.913978495	NA	NA
DMRcontig44064:63800291	contig44064	63800291	63800519	229	1	4.37E-06	11	4.80349345	NA	NA
DMRcontig44112:63866644	contig44112	63866644	63866939	296	2	8.38E-08	10	3.378378378	NA	NA
DMRcontig44132:63893207	contig44132	63893207	63893400	194	1	2.13E-12	1	0.515463918	NA	NA
DMRcontig44174:63949601	contig44174	63949601	63950025	425	1	9.74E-06	30	7.058823529	LOC101860977	Unknown
DMRcontig44266:64079201	contig44266	64079201	64079570	370	2	4.63E-07	11	2.972972973	NA	NA
DMRcontig44563:64480609	contig44563	64480609	64480900	292	1	6.36E-09	7	2.397260274	NA	NA
DMRcontig44586:64510601	contig44586	64510601	64510984	384	2	1.00E-13	13	3.385416667	NA	NA
DMRcontig44596:64524211	contig44596	64524211	64524400	190	1	2.57E-06	16	8.421052632	NA	NA
DMRcontig44793:64791710	contig44793	64791710	64791900	191	1	1.35E-06	5	2.617801047	NA	NA
DMRcontig44794:64793201	contig44794	64793201	64793300	100	1	9.17E-06	1	1	NA	NA
DMRcontig44819:64827701	contig44819	64827701	64827900	200	1	5.57E-10	12	6	NA	NA
DMRcontig44869:64894090	contig44869	64894090	64894500	411	2	1.61E-07	15	3.649635036	NA	NA
DMRcontig45105:65210301	contig45105	65210301	65210531	231	1	5.73E-07	5	2.164502165	LOC101848867	Metabolism
DMRcontig45169:65298718	contig45169	65298718	65299100	383	2	6.75E-07	10	2.610966057	NA	NA
DMRcontig45302:65478904	contig45302	65478904	65479053	150	1	1.01E-06	0	0	NA	NA
DMRcontig45482:65721165	contig45482	65721165	65721356	192	1	2.26E-06	9	4.6875	NA	NA
DMRcontig45540:65798701	contig45540	65798701	65798947	247	1	1.87E-06	2	0.809716599	NA	NA
DMRcontig45638:65929101	contig45638	65929101	65929200	100	1	7.64E-06	1	1	NA	NA
DMRcontig45856:66218581	contig45856	66218581	66218818	238	1	5.30E-07	9	3.781512605	NA	NA
DMRcontig45867:66233701	contig45867	66233701	66234013	313	1	6.17E-07	4	1.277955272	NA	NA
DMRcontig45871:66239077	contig45871	66239077	66239473	397	1	2.58E-09	6	1.511335013	NA	NA
DMRcontig46118:66572350	contig46118	66572350	66573085	736	1	8.85E-06	16	2.173913043	NA	NA
DMRcontig46341:66871701	contig46341	66871701	66872100	400	2	2.66E-13	36	9	NA	NA
DMRcontig46399:66948224	contig46399	66948224	66948400	177	1	6.31E-07	4	2.259887006	NA	NA
DMRcontig46432:66990539	contig46432	66990539	66990700	162	2	7.48E-09	2	1.234567901	NA	NA
DMRcontig46443:67005301	contig46443	67005301	67005600	300	1	3.91E-06	13	4.333333333	NA	NA
DMRcontig46473:67046301	contig46473	67046301	67046600	300	2	2.21E-09	10	3.333333333	NA	NA
DMRcontig46542:67136022	contig46542	67136022	67136761	740	3	7.86E-09	49	6.621621622	NA	NA
DMRcontig46688:67328114	contig46688	67328114	67328247	134	1	8.14E-06	3	2.23880597	NA	NA
DMRcontig46699:67342301	contig46699	67342301	67342462	162	1	8.60E-07	3	1.851851852	NA	NA
DMRcontig46836:67524734	contig46836	67524734	67524895	162	1	8.46E-06	9	5.555555556	NA	NA
DMRcontig47017:67762257	contig47017	67762257	67762400	144	1	4.59E-06	1	0.694444444	NA	NA
DMRcontig47018:67763543	contig47018	67763543	67763800	258	1	7.30E-07	8	3.100775194	NA	NA
DMRcontig47019:67765001	contig47019	67765001	67765387	387	1	3.34E-06	11	2.842377261	NA	NA
DMRcontig47034:67784301	contig47034	67784301	67784600	300	1	2.44E-06	11	3.666666667	NA	NA
DMRcontig47036:67787201	contig47036	67787201	67787300	100	1	2.10E-06	7	7	NA	NA
DMRcontig47428:68303040	contig47428	68303040	68303292	253	1	6.84E-08	8	3.162055336	NA	NA
DMRcontig47548:68460301	contig47548	68460301	68461077	777	1	2.20E-07	35	4.504504505	NA	NA
DMRcontig47578:68501701	contig47578	68501701	68502200	500	1	7.17E-07	11	2.2	NA	NA
DMRcontig47719:68687501	contig47719	68687501	68687694	194	1	6.44E-06	6	3.092783505	NA	NA
DMRcontig47820:68817411	contig47820	68817411	68817680	270	2	7.69E-07	7	2.592592593	NA	NA
DMRcontig47846:68851845	contig47846	68851845	68852000	156	1	7.46E-06	5	3.205128205	NA	NA
DMRcontig47892:68913609	contig47892	68913609	68913900	292	1	3.39E-06	6	2.054794521	NA	NA
DMRcontig47900:68923681	contig47900	68923681	68923800	120	1	1.12E-06	2	1.666666667	NA	NA
DMRcontig47999:69052267	contig47999	69052267	69052575	309	1	4.28E-07	8	2.588996764	NA	NA
DMRcontig48081:69159201	contig48081	69159201	69159541	341	2	1.61E-07	13	3.812316716	NA	NA
DMRcontig48094:69175552	contig48094	69175552	69175700	149	2	5.54E-09	6	4.026845638	NA	NA
DMRcontig48114:69202401	contig48114	69202401	69202585	185	1	1.82E-11	7	3.783783784	NA	NA
DMRcontig48131:69224201	contig48131	69224201	69224328	128	1	2.68E-13	0	0	NA	NA
DMRcontig48147:69244723	contig48147	69244723	69244900	178	1	7.46E-06	2	1.123595506	NA	NA
DMRcontig48272:69406855	contig48272	69406855	69407100	246	1	6.64E-08	4	1.62601626	NA	NA
DMRcontig48318:69467101	contig48318	69467101	69467500	400	2	3.02E-06	10	2.5	NA	NA
DMRcontig48340:69495001	contig48340	69495001	69495179	179	1	3.72E-07	8	4.469273743	NA	NA
DMRcontig48397:69569601	contig48397	69569601	69569796	196	1	2.11E-07	7	3.571428571	NA	NA
DMRcontig48430:69613453	contig48430	69613453	69613700	248	1	7.11E-07	9	3.629032258	NA	NA
DMRcontig48473:69668401	contig48473	69668401	69668500	100	1	8.79E-06	5	5	NA	NA
DMRcontig48480:69677880	contig48480	69677880	69678174	295	3	1.65E-12	5	1.694915254	NA	NA
DMRcontig48715:69983515	contig48715	69983515	69983813	299	1	5.38E-06	11	3.678929766	NA	NA
DMRcontig48905:70227601	contig48905	70227601	70227900	300	1	5.74E-06	7	2.333333333	NA	NA
DMRcontig48930:70258401	contig48930	70258401	70258600	200	1	8.91E-06	5	2.5	NA	NA
DMRcontig49020:70371462	contig49020	70371462	70371693	232	3	9.07E-20	11	4.74137931	LDAH	Unknown
DMRcontig49124:70505098	contig49124	70505098	70505453	356	1	7.89E-06	13	3.651685393	NA	NA
DMRcontig49233:70641926	contig49233	70641926	70642300	375	2	6.70E-07	26	6.933333333	NA	NA
DMRcontig49399:70853101	contig49399	70853101	70853400	300	1	7.84E-06	17	5.666666667	NA	NA

DMRcontig49614:71126304	contig49614	71126304	71127126	823	1	6.53E-06	28	3.40218712	NA	NA
DMRcontig50071:71694401	contig50071	71694401	71694700	300	1	9.84E-07	22	7.333333333	NA	NA
DMRcontig50132:71771001	contig50132	71771001	71771390	390	1	7.56E-06	9	2.307692308	NA	NA
DMRcontig50270:71942101	contig50270	71942101	71942345	245	1	2.44E-06	11	4.489795918	NA	NA
DMRcontig50569:72325794	contig50569	72325794	72326062	269	2	1.38E-06	6	2.230483271	NA	NA
DMRcontig50684:72473201	contig50684	72473201	72473538	338	1	7.55E-06	6	1.775147929	NA	NA
DMRcontig50708:72503401	contig50708	72503401	72503587	187	1	1.56E-06	6	3.20855615	NA	NA
DMRcontig50737:72541557	contig50737	72541557	72542000	444	1	7.33E-08	14	3.153153153	NA	NA
DMRcontig50765:72579302	contig50765	72579302	72579900	599	2	3.32E-06	29	4.841402337	NA	NA
DMRcontig50863:72707577	contig50863	72707577	72708000	424	1	3.91E-06	17	4.009433962	NA	NA
DMRcontig50936:72801631	contig50936	72801631	72801885	255	3	4.31E-20	12	4.705882353	NA	NA
DMRcontig51062:72959501	contig51062	72959501	72960000	500	1	4.40E-06	11	2.2	NA	NA
DMRcontig51228:73169201	contig51228	73169201	73169436	236	1	4.51E-06	12	5.084745763	NA	NA
DMRcontig51282:73236101	contig51282	73236101	73236311	211	3	6.14E-83	6	2.843601896	NA	NA
DMRcontig51292:73249201	contig51292	73249201	73249700	500	3	6.14E-09	28	5.6	NA	NA
DMRcontig51469:73476301	contig51469	73476301	73476600	300	3	8.48E-15	9	3	NA	NA
DMRcontig51522:73544801	contig51522	73544801	73545320	520	2	1.43E-08	27	5.192307692	NA	NA
DMRcontig51622:73674501	contig51622	73674501	73674800	300	2	8.01E-09	5	1.666666667	NA	NA
DMRcontig51715:73793101	contig51715	73793101	73793398	298	1	7.57E-07	11	3.691275168	NA	NA
DMRcontig51729:73810701	contig51729	73810701	73810864	164	1	2.57E-06	2	1.219512195	NA	NA
DMRcontig51734:73817101	contig51734	73817101	73817300	200	2	1.31E-06	7	3.5	NA	NA
DMRcontig51739:73823201	contig51739	73823201	73823583	383	1	8.01E-06	9	2.349869452	NA	NA

Supplemental Table S6
R2-F1 vs R1-F0 DMR Table 1e-05

DMR Name	Chr	Start	Stop	Length	# Sig Win	minP	CpG #	CpG Density	Gene Annotation	Gene Category
DMRcontig00012:29574	contig00012	29574	31100	1527	1	7.76E-06	30	1.964636542	COX1	Metabolism
DMRcontig00016:39129	contig00016	39129	39900	772	1	9.33E-06	34	4.404145078	NA	NA
DMRcontig00140:328001	contig00140	328001	328252	252	1	1.03E-06	7	2.777777778	NA	NA
DMRcontig00177:410001	contig00177	410001	410855	855	1	7.89E-06	34	3.976608187	NA	NA
DMRcontig00256:585501	contig00256	585501	586100	600	1	4.12E-08	43	7.166666667	NA	NA
DMRcontig00272:619701	contig00272	619701	620300	600	1	9.95E-06	38	6.333333333	LOC106602804;LOC106609995;rps6	Metabolism
DMRcontig00311:705801	contig00311	705801	706500	700	1	8.74E-06	31	4.428571429	LOC101851960	Unknown; Metabolism
DMRcontig00365:824701	contig00365	824701	825600	900	1	7.27E-09	16	1.777777778	NA	NA
DMRcontig00373:841601	contig00373	841601	842200	600	1	4.57E-06	38	6.333333333	LOC101061694;TUBB4B	Metabolism
DMRcontig00386:868919	contig00386	868919	869200	282	3	1.24E-19	4	1.418439716	NA	NA
DMRcontig00417:936401	contig00417	936401	937600	1200	1	2.48E-07	63	5.25	NA	NA
DMRcontig00576:1273601	contig00576	1273601	1274800	1200	1	2.40E-07	80	6.666666667	NA	NA
DMRcontig00711:1552501	contig00711	1552501	1553972	1472	1	2.29E-06	48	3.260869565	LOC101857978	Development
DMRcontig00731:1594925	contig00731	1594925	1596300	1376	1	1.83E-06	40	2.906976744	NA	NA
DMRcontig00863:1860830	contig00863	1860830	1861100	271	2	7.27E-06	11	4.05904059	NA	NA
DMRcontig00923:1984901	contig00923	1984901	1985600	700	1	9.28E-07	31	4.428571429	bcas2	Cytoskeleton
DMRcontig00955:2048301	contig00955	2048301	2049500	1200	1	8.26E-06	36	3	NA	NA
DMRcontig00982:2103401	contig00982	2103401	2103800	400	4	7.88E-19	8	2	zbtb8os	Metabolism
DMRcontig01427:3004601	contig01427	3004601	3005000	400	1	3.65E-06	16	4	NA	NA
DMRcontig01749:3631057	contig01749	3631057	3631600	544	3	2.53E-18	9	1.654411765	NA	NA
DMRcontig01754:3641427	contig01754	3641427	3642300	874	1	7.37E-06	28	3.203661327	NA	NA
DMRcontig01988:4098901	contig01988	4098901	4099100	200	1	9.27E-06	8	4	NA	NA
DMRcontig02055:4225706	contig02055	4225706	4226200	495	1	7.90E-07	8	1.616161616	ATP8;COX1	Metabolism
DMRcontig02240:4575601	contig02240	4575601	4576300	700	1	4.21E-07	28	4	NA	NA
DMRcontig02247:4587801	contig02247	4587801	4588300	500	1	4.10E-07	40	8	NA	NA
DMRcontig02444:4954601	contig02444	4954601	4954800	200	1	4.48E-07	10	5	NA	NA
DMRcontig02800:5607801	contig02800	5607801	5608100	300	1	3.65E-07	18	6	NA	NA
DMRcontig02909:5808856	contig02909	5808856	5809300	445	1	7.50E-07	22	4.943820225	NA	NA
DMRcontig02944:5872220	contig02944	5872220	5872700	481	1	3.19E-06	33	6.860706861	NA	NA
DMRcontig03089:6131134	contig03089	6131134	6131300	167	1	4.49E-06	2	1.19760479	NA	NA
DMRcontig03390:6673785	contig03390	6673785	6674100	316	1	7.87E-06	15	4.746835443	NA	NA
DMRcontig03415:6717501	contig03415	6717501	6717800	300	1	1.24E-06	18	6	NA	NA
DMRcontig03533:6925116	contig03533	6925116	6925400	285	2	3.68E-06	19	6.666666667	NA	NA
DMRcontig03588:7022661	contig03588	7022661	7023200	540	1	9.39E-06	21	3.888888889	NA	NA
DMRcontig03641:7113301	contig03641	7113301	7114400	1100	1	9.04E-06	75	6.818181818	NA	NA
DMRcontig03805:7405701	contig03805	7405701	7405875	175	2	1.16E-06	11	6.285714286	NA	NA
DMRcontig03956:7671201	contig03956	7671201	7671800	600	1	2.71E-06	27	4.5	NA	NA
DMRcontig04098:7923101	contig04098	7923101	7924200	1100	1	7.48E-06	35	3.181818182	NA	NA
DMRcontig05024:9524101	contig05024	9524101	9524600	500	1	8.46E-06	13	2.6	LOC105777419	Translation
DMRcontig05209:9845001	contig05209	9845001	9846298	1298	1	1.62E-06	60	4.622496148	NA	NA
DMRcontig05357:10096701	contig05357	10096701	10097100	400	1	2.70E-09	19	4.75	NA	NA
DMRcontig05429:10217901	contig05429	10217901	10218300	400	1	1.91E-06	13	3.25	NA	NA
DMRcontig05564:10445943	contig05564	10445943	10447087	1145	1	7.43E-07	28	2.445414847	NA	NA
DMRcontig06167:11461501	contig06167	11461501	11461851	351	2	1.49E-10	23	6.552706553	NA	NA
DMRcontig06228:11562301	contig06228	11562301	11562900	600	1	2.52E-07	42	7	NA	NA
DMRcontig06304:11690228	contig06304	11690228	11690800	573	1	5.64E-06	39	6.806282723	Dmoj\GI14614;Dpse\GA14719	Cytoskeleton
DMRcontig06362:11786823	contig06362	11786823	11787500	678	1	7.08E-07	39	5.752212389	NA	NA
DMRcontig06475:11973601	contig06475	11973601	11973900	300	1	8.51E-09	24	8	NA	NA
DMRcontig06626:12221701	contig06626	12221701	12222600	900	1	9.20E-06	54	6	LOC105398638;hspa5	Metabolism
DMRcontig06640:12245401	contig06640	12245401	12245700	300	1	1.82E-06	9	3	Dere\GG15112;Hsp83	Unknown
DMRcontig06665:12285801	contig06665	12285801	12285862	62	1	8.24E-07	0	0	NA	NA
DMRcontig06712:12362401	contig06712	12362401	12363100	700	1	9.99E-06	39	5.571428571	CG46317;dy	Signaling
DMRcontig07022:12871101	contig07022	12871101	12871182	82	1	9.04E-07	0	0	NA	NA
DMRcontig07149:13078101	contig07149	13078101	13078300	200	1	6.14E-07	3	1.5	NA	NA
DMRcontig07172:13114901	contig07172	13114901	13115261	361	2	2.12E-11	6	1.662049861	NA	NA
DMRcontig07612:13821111	contig07612	13821111	13821500	390	1	6.79E-06	6	1.538461538	NA	NA
DMRcontig07851:14210943	contig07851	14210943	14211700	758	1	3.84E-06	18	2.374670185	NA	NA
DMRcontig07962:14389101	contig07962	14389101	14390400	1300	1	8.97E-07	32	2.461538462	NA	NA

DMRcontig08038:14512724	contig08038	14512724	14513500	777	1	5.62E-09	51	6.563706564	NA	NA
DMRcontig08247:14847101	contig08247	14847101	14847600	500	1	2.19E-06	23	4.6	NA	NA
DMRcontig08310:14949782	contig08310	14949782	14950377	596	1	1.43E-07	3	0.503355705	NA	NA
DMRcontig08357:15026701	contig08357	15026701	15026900	200	1	2.40E-07	9	4.5	NA	NA
DMRcontig08377:15058601	contig08377	15058601	15059700	1100	1	9.73E-06	26	2.363636364	NA	NA
DMRcontig08412:15114788	contig08412	15114788	15115240	453	2	3.39E-06	11	2.428256071	NA	NA
DMRcontig08446:15171250	contig08446	15171250	15171800	551	1	4.54E-06	6	1.08892922	NA	NA
DMRcontig08534:15309401	contig08534	15309401	15309771	371	1	5.06E-10	18	4.851752022	NA	NA
DMRcontig08655:15503901	contig08655	15503901	15504000	100	1	1.31E-06	1	1	NA	NA
DMRcontig08867:15837901	contig08867	15837901	15838500	600	1	8.51E-07	15	2.5	NA	NA
DMRcontig09129:16252212	contig09129	16252212	16253000	789	4	1.89E-11	42	5.323193916	CpipJ_CPIJ017745;FAXDC2	Binding Protein
DMRcontig09150:16287101	contig09150	16287101	16287600	500	1	3.56E-06	39	7.8	NA	NA
DMRcontig09454:16764801	contig09454	16764801	16765100	300	1	3.34E-06	7	2.333333333	NA	NA
DMRcontig09634:17048001	contig09634	17048001	17048500	500	1	2.72E-06	26	5.2	NA	NA
DMRcontig09781:17279101	contig09781	17279101	17279500	400	1	3.72E-06	4	1	NA	NA
DMRcontig10007:17627406	contig10007	17627406	17628300	895	1	2.07E-06	12	1.340782123	NA	NA
DMRcontig10295:18074101	contig10295	18074101	18074200	100	1	9.35E-06	3	3	NA	NA
DMRcontig10834:18900101	contig10834	18900101	18900799	699	1	2.29E-06	34	4.864091559	NA	NA
DMRcontig11187:19434301	contig11187	19434301	19434400	100	1	7.00E-08	1	1	NA	NA
DMRcontig11262:19549401	contig11262	19549401	19549806	406	2	3.29E-07	14	3.448275862	NA	NA
DMRcontig11370:19711276	contig11370	19711276	19711697	422	1	2.26E-08	8	1.895734597	NA	NA
DMRcontig11491:19894901	contig11491	19894901	19895074	174	1	2.61E-07	6	3.448275862	NA	NA
DMRcontig11534:19960001	contig11534	19960001	19960400	400	3	1.63E-11	22	5.5	NA	NA
DMRcontig11593:20046749	contig11593	20046749	20047184	436	4	3.40E-15	13	2.981651376	NA	NA
DMRcontig11731:20254201	contig11731	20254201	20254700	500	1	4.32E-06	34	6.8	NA	NA
DMRcontig11781:20327801	contig11781	20327801	20328000	200	1	7.06E-06	5	2.5	CGB_A0010C;CGB_A6680C;GUITHDRAFT_104274	Translation
DMRcontig11987:20639101	contig11987	20639101	20639400	300	1	4.72E-06	10	3.333333333	NA	NA
DMRcontig12215:20982931	contig12215	20982931	20983645	715	3	1.17E-07	11	1.538461538	NA	NA
DMRcontig12225:20999001	contig12225	20999001	20999400	400	1	4.82E-06	19	4.75	NA	NA
DMRcontig12766:21811131	contig12766	21811131	21811700	570	1	1.02E-07	21	3.684210526	NA	NA
DMRcontig12905:22016401	contig12905	22016401	22016800	400	1	7.49E-06	40	10	NA	NA
DMRcontig12997:22151301	contig12997	22151301	22151751	451	1	1.19E-06	6	1.33037694	NA	NA
DMRcontig13033:22204443	contig13033	22204443	22204800	358	2	5.31E-07	3	0.837988827	NA	NA
DMRcontig13121:22334801	contig13121	22334801	22335300	500	3	7.83E-15	34	6.8	NA	NA
DMRcontig13141:22365643	contig13141	22365643	22366200	558	2	1.59E-08	20	3.584229391	NA	NA
DMRcontig13326:22638801	contig13326	22638801	22639100	300	1	1.35E-06	16	5.333333333	NA	NA
DMRcontig13443:22812001	contig13443	22812001	22812300	300	1	8.02E-06	19	6.333333333	NA	NA
DMRcontig13636:23103469	contig13636	23103469	23104200	732	1	2.90E-07	33	4.508196721	NA	NA
DMRcontig13810:23361901	contig13810	23361901	23362345	445	1	7.54E-07	14	3.146067416	NA	NA
DMRcontig13936:23548401	contig13936	23548401	23548600	200	1	4.09E-07	4	2	LOC107987209;LOC107984688;LOC108168064;LOC107973554;HOXD10;Spon1;PDE1B	Transcription; Metabolism
DMRcontig14615:24551401	contig14615	24551401	24551600	200	1	5.35E-07	2	1	NA	NA
DMRcontig15044:25183608	contig15044	25183608	25183900	293	1	7.65E-06	9	3.071672355	NA	NA
DMRcontig15065:25214609	contig15065	25214609	25214800	192	1	5.17E-09	2	1.041666667	NA	NA
DMRcontig15403:25712001	contig15403	25712001	25712190	190	1	1.54E-07	1	0.526315789	NA	NA
DMRcontig15465:25801701	contig15465	25801701	25802000	300	1	8.01E-06	6	2	NA	NA
DMRcontig15491:25840916	contig15491	25840916	25841170	255	2	2.29E-07	4	1.568627451	NA	NA
DMRcontig15839:26345437	contig15839	26345437	26345600	164	1	3.39E-06	7	4.268292683	LOC106588560	Transcription
DMRcontig16218:26896001	contig16218	26896001	26896300	300	1	4.31E-06	15	5	NA	NA
DMRcontig16331:27061038	contig16331	27061038	27061554	517	1	5.37E-06	48	9.284332689	NA	NA
DMRcontig16488:27287413	contig16488	27287413	27287678	266	1	4.29E-06	10	3.759398496	NA	NA
DMRcontig17090:28158201	contig17090	28158201	28158300	100	1	3.43E-06	2	2	NA	NA
DMRcontig17134:28220111	contig17134	28220111	28220300	190	1	6.40E-06	2	1.052631579	NA	NA
DMRcontig17306:28464336	contig17306	28464336	28464600	265	1	1.60E-07	2	0.754716981	NA	NA
DMRcontig17739:29077444	contig17739	29077444	29078000	557	1	3.11E-06	11	1.97486535	NA	NA
DMRcontig18070:29543801	contig18070	29543801	29544208	408	2	1.09E-07	16	3.921568627	NA	NA
DMRcontig18129:29628501	contig18129	29628501	29628800	300	2	1.90E-06	7	2.333333333	NA	NA
DMRcontig18196:29723221	contig18196	29723221	29723925	705	1	7.63E-06	9	1.276595745	NA	NA
DMRcontig18443:30074301	contig18443	30074301	30074552	252	1	8.21E-09	13	5.158730159	NA	NA
DMRcontig18453:30088401	contig18453	30088401	30089170	770	1	3.56E-06	20	2.597402597	NA	NA
DMRcontig19055:31000901	contig19055	31000901	31001068	168	1	2.07E-06	2	1.19047619	NA	NA
DMRcontig19089:31050838	contig19089	31050838	31051300	463	2	6.88E-09	7	1.51187905	NA	NA

DMRcontig19530:31707201	contig19530	31707201	31707420	220	1	1.65E-06	2	0.909090909	NA	NA
DMRcontig19622:31839182	contig19622	31839182	31839300	119	1	9.46E-09	1	0.840336134	NA	NA
DMRcontig19631:31851601	contig19631	31851601	31851800	200	2	1.19E-06	5	2.5	NA	NA
DMRcontig19643:31870301	contig19643	31870301	31870600	300	1	5.58E-06	18	6	NA	NA
DMRcontig19954:32324401	contig19954	32324401	32324700	300	1	7.33E-08	5	1.666666667	NA	NA
DMRcontig20130:32598701	contig20130	32598701	32599000	300	1	8.10E-06	14	4.666666667	NA	NA
DMRcontig20335:32911901	contig20335	32911901	32912300	400	1	2.99E-06	29	7.25	NA	NA
DMRcontig20610:33289320	contig20610	33289320	33289500	181	2	4.39E-19	0	0	NA	NA
DMRcontig20887:33643901	contig20887	33643901	33644100	200	1	7.49E-06	0	0	NA	NA
DMRcontig20927:33695358	contig20927	33695358	33695462	105	1	8.03E-07	0	0	NA	NA
DMRcontig21235:34097060	contig21235	34097060	34097126	67	2	3.85E-06	0	0	NA	NA
DMRcontig21685:34590746	contig21685	34590746	34590797	52	1	8.60E-07	0	0	NA	NA
DMRcontig22580:35618601	contig22580	35618601	35618759	159	1	1.69E-06	1	0.628930818	NA	NA
DMRcontig24157:37512801	contig24157	37512801	37513700	900	1	3.67E-08	29	3.222222222	NA	NA
DMRcontig24993:38626601	contig24993	38626601	38627000	400	1	4.95E-06	25	6.25	LOC102794729;rpl23a	Metabolism
DMRcontig26379:40222001	contig26379	40222001	40222238	238	1	4.31E-10	2	0.840336134	NA	NA
DMRcontig26520:40386660	contig26520	40386660	40386732	73	1	2.16E-06	0	0	NA	NA
DMRcontig26614:40496401	contig26614	40496401	40496587	187	1	7.72E-07	22	11.76470588	NA	NA
DMRcontig27989:42317432	contig27989	42317432	42317800	369	1	2.07E-06	17	4.60704607	NA	NA
DMRcontig28086:42442715	contig28086	42442715	42442775	61	1	3.39E-06	1	1.639344262	NA	NA
DMRcontig28476:42951401	contig28476	42951401	42951700	300	1	1.08E-06	14	4.666666667	NA	NA
DMRcontig29210:43841901	contig29210	43841901	43842300	400	1	8.31E-08	14	3.5	NA	NA
DMRcontig30139:44931197	contig30139	44931197	44931249	53	1	7.48E-06	1	1.886792453	NA	NA
DMRcontig30771:45689401	contig30771	45689401	45689500	100	1	7.65E-06	4	4	NA	NA
DMRcontig31516:46680001	contig31516	46680001	46680146	146	1	5.83E-08	3	2.054794521	NA	NA
DMRcontig31581:46767901	contig31581	46767901	46768085	185	1	3.28E-06	2	1.081081081	NA	NA
DMRcontig32074:47385113	contig32074	47385113	47385199	87	1	8.11E-11	0	0	NA	NA
DMRcontig32445:47814278	contig32445	47814278	47814338	61	2	5.61E-07	0	0	NA	NA
DMRcontig33851:49418714	contig33851	49418714	49418775	62	1	1.87E-07	0	0	NA	NA
DMRcontig33899:49474225	contig33899	49474225	49474273	49	1	2.62E-07	0	0	NA	NA
DMRcontig34510:50180435	contig34510	50180435	50180481	47	1	2.69E-06	0	0	NA	NA
DMRcontig35321:51263501	contig35321	51263501	51264000	500	2	1.54E-10	20	4	NA	NA
DMRcontig35857:52084658	contig35857	52084658	52085100	443	3	1.10E-08	28	6.320541761	NA	NA
DMRcontig35949:52221701	contig35949	52221701	52221900	200	1	1.05E-07	3	1.5	NA	NA
DMRcontig36371:52848995	contig36371	52848995	52849448	454	2	8.34E-09	32	7.04845815	NA	NA
DMRcontig36581:53161201	contig36581	53161201	53161400	200	2	6.16E-08	3	1.5	NA	NA
DMRcontig36684:53316311	contig36684	53316311	53316500	190	1	1.45E-06	4	2.105263158	NA	NA
DMRcontig36714:53362201	contig36714	53362201	53362308	108	1	5.94E-06	0	0	NA	NA
DMRcontig37054:53862153	contig37054	53862153	53862600	448	2	1.46E-11	20	4.464285714	NA	NA
DMRcontig37275:54189766	contig37275	54189766	54190000	235	1	7.67E-07	12	5.106382979	NA	NA
DMRcontig37325:54264201	contig37325	54264201	54264579	379	1	2.95E-06	10	2.638522427	NA	NA
DMRcontig38674:56237101	contig38674	56237101	56237583	483	3	4.17E-10	31	6.418219462	NA	NA
DMRcontig38822:56452101	contig38822	56452101	56452578	478	1	3.62E-06	18	3.765690377	NA	NA
DMRcontig38856:56501501	contig38856	56501501	56501800	300	1	1.36E-08	9	3	NA	NA
DMRcontig38993:56700401	contig38993	56700401	56700500	100	1	8.29E-07	7	7	NA	NA
DMRcontig39411:57300501	contig39411	57300501	57300800	300	1	4.95E-06	6	2	NA	NA
DMRcontig39430:57328114	contig39430	57328114	57328400	287	1	6.21E-06	0	0	NA	NA
DMRcontig40122:58322101	contig40122	58322101	58322400	300	1	1.29E-08	17	5.666666667	NA	NA
DMRcontig40436:58768601	contig40436	58768601	58769084	484	4	2.48E-12	14	2.892561983	NA	NA
DMRcontig40718:59164801	contig40718	59164801	59165000	200	1	1.65E-06	14	7	NA	NA
DMRcontig40884:59398701	contig40884	59398701	59398952	252	1	4.57E-07	17	6.746031746	NA	NA
DMRcontig41820:60709201	contig41820	60709201	60709600	400	1	6.75E-08	10	2.5	NA	NA
DMRcontig42799:62068945	contig42799	62068945	62069100	156	1	7.63E-07	1	0.641025641	NA	NA
DMRcontig43037:62396434	contig43037	62396434	62396800	367	1	2.29E-11	5	1.36239782	NA	NA
DMRcontig43187:62602001	contig43187	62602001	62602190	190	1	1.79E-06	5	2.631578947	NA	NA
DMRcontig43373:62855544	contig43373	62855544	62856100	557	2	3.19E-07	36	6.463195691	NA	NA
DMRcontig44132:63893207	contig44132	63893207	63893400	194	1	1.43E-07	1	0.515463918	NA	NA
DMRcontig44209:63998316	contig44209	63998316	63998951	636	1	9.23E-06	20	3.144654088	NA	NA
DMRcontig44749:64732049	contig44749	64732049	64732298	250	1	8.09E-07	5	2	NA	NA
DMRcontig44819:64827701	contig44819	64827701	64828000	300	2	3.71E-08	16	5.333333333	NA	NA
DMRcontig45520:65772434	contig45520	65772434	65772777	344	2	2.20E-09	19	5.523255814	NA	NA
DMRcontig46432:66990539	contig46432	66990539	66990700	162	1	3.68E-06	2	1.234567901	NA	NA
DMRcontig46542:67136022	contig46542	67136022	67136761	740	3	1.28E-08	49	6.621621622	NA	NA
DMRcontig47034:67784401	contig47034	67784401	67784600	200	1	3.49E-06	8	4	NA	NA

DMRcontig47036:67787101	contig47036	67787101	67787600	500	1	2.18E-06	32	6.4	NA	NA
DMRcontig47326:68170101	contig47326	68170101	68170400	300	1	8.59E-06	16	5.333333333	NA	NA
DMRcontig47797:68788503	contig47797	68788503	68788900	398	1	5.92E-06	8	2.010050251	LOC106959388	Epigenetic
DMRcontig48094:69175552	contig48094	69175552	69175700	149	2	2.03E-08	6	4.026845638	NA	NA
DMRcontig48114:69202401	contig48114	69202401	69202585	185	1	3.70E-09	7	3.783783784	NA	NA
DMRcontig48131:69224201	contig48131	69224201	69224328	128	1	7.22E-11	0	0	NA	NA
DMRcontig48220:69338415	contig48220	69338415	69338600	186	2	3.16E-07	1	0.537634409	NA	NA
DMRcontig48905:70227601	contig48905	70227601	70227800	200	1	1.91E-06	5	2.5	NA	NA
DMRcontig48927:70254701	contig48927	70254701	70254900	200	1	6.02E-06	6	3	NA	NA
DMRcontig49020:70371462	contig49020	70371462	70371693	232	3	9.14E-13	11	4.74137931	LDAH	Signaling
DMRcontig49309:70740101	contig49309	70740101	70740500	400	1	2.15E-06	6	1.5	NA	NA
DMRcontig50208:71863609	contig50208	71863609	71864000	392	1	5.50E-06	10	2.551020408	NA	NA
DMRcontig50666:72449122	contig50666	72449122	72449700	579	2	3.77E-06	12	2.07253886	NA	NA
DMRcontig50936:72801631	contig50936	72801631	72801885	255	1	1.08E-06	12	4.705882353	NA	NA
DMRcontig51282:73236101	contig51282	73236101	73236311	211	2	9.88E-28	6	2.843601896	NA	NA
DMRcontig51469:73476301	contig51469	73476301	73476600	300	1	3.62E-06	9	3	NA	NA
DMRcontig51622:73674501	contig51622	73674501	73674800	300	1	7.92E-06	5	1.666666667	NA	NA

Supplemental Table S7
R2-F1 vs. Lake Washington L2 DMR Table 1e-05

DMR Name	Chr	Start	Stop	Length	# Sig Win	minP	CpG #	CpG Density	Gene Annotation	Gene Category
DMRcontig00012:29574	contig00012	29574	30400	827	5	7.4951E-12	16	1.93470375	COX1	Metabolism
DMRcontig00017:41301	contig00017	41301	42100	800	1	9.01E-06	38	4.75	NA	NA
DMRcontig00023:54401	contig00023	54401	54900	500	1	5.1652E-06	14	2.8	NA	NA
DMRcontig00034:79101	contig00034	79101	79800	700	2	2.0607E-07	24	3.42857143	LOC109538865;ndufv2	Unknown;Metabolism
DMRcontig00039:91401	contig00039	91401	92400	1000	1	6.4319E-06	36	3.6	eif2s3	Translation
DMRcontig00053:125401	contig00053	125401	126100	700	1	1.951E-06	7	1	NA	NA
DMRcontig00059:142801	contig00059	142801	143530	730	2	1.0348E-11	47	6.43835616	NA	NA
DMRcontig00087:207101	contig00087	207101	208000	900	1	1.2318E-07	35	3.88888889	LOC107219277	Cytoskeleton
DMRcontig00096:227101	contig00096	227101	227800	700	1	1.9568E-07	12	1.71428571	NA	NA
DMRcontig00105:246701	contig00105	246701	247300	600	3	2.0264E-11	22	3.66666667	NA	NA
DMRcontig00119:278901	contig00119	278901	279600	700	2	1.5877E-08	37	5.28571429	LOC102731623;HPN	Proteolysis;Unknown
DMRcontig00155:360401	contig00155	360401	360800	400	1	6.8264E-06	4	1	NA	NA
DMRcontig00178:412001	contig00178	412001	413200	1200	2	4.1987E-07	25	2.08333333	NA	NA
DMRcontig00246:562801	contig00246	562801	563200	400	1	2.8447E-06	6	1.5	NA	NA
DMRcontig00279:634537	contig00279	634537	635500	964	1	3.7722E-06	10	1.0373444	ATP8	Metabolism
DMRcontig00285:648301	contig00285	648301	649131	831	5	5.0483E-09	15	1.80505415	COX1	Metabolism
DMRcontig00311:706301	contig00311	706301	706500	200	1	3.3646E-06	8	4	LOC101851960	Mitochondria
DMRcontig00313:710901	contig00313	710901	712100	1200	1	7.6377E-07	49	4.08333333	NA	NA
DMRcontig00328:746116	contig00328	746116	747200	1085	1	1.1167E-06	24	2.21198157	NA	NA
DMRcontig00386:868919	contig00386	868919	869200	282	2	1.5538E-31	4	1.41843972	NA	NA
DMRcontig00388:873401	contig00388	873401	873900	500	1	1.7658E-11	7	1.4	NA	NA
DMRcontig00396:891101	contig00396	891101	891600	500	1	8.0185E-06	13	2.6	NA	NA
DMRcontig00410:921780	contig00410	921780	922896	1117	1	4.8879E-06	46	4.11817368	CAOG_01866;Pepck	Metabolism
DMRcontig00416:934401	contig00416	934401	935384	984	1	9.8695E-06	31	3.1504065	NA	NA
DMRcontig00417:936401	contig00417	936401	937400	1000	2	2.0482E-06	60	6	NA	NA
DMRcontig00428:961001	contig00428	961001	961600	600	1	1.6193E-08	15	2.5	SPRG_12594	Cytoskeleton
DMRcontig00443:992101	contig00443	992101	992600	500	1	6.5121E-06	30	6	NA	NA
DMRcontig00449:1005301	contig00449	1005301	1005700	400	3	8.5187E-26	39	9.75	NA	NA
DMRcontig00472:1056001	contig00472	1056001	1056300	300	1	3.4116E-06	3	1	NA	NA
DMRcontig00497:1109352	contig00497	1109352	1110000	649	1	8.075E-07	34	5.23882897	NA	NA
DMRcontig00498:1111401	contig00498	1111401	1111800	400	1	7.9323E-07	22	5.5	NA	NA
DMRcontig00522:1161401	contig00522	1161401	1161900	500	1	1.5645E-06	9	1.8	NA	NA
DMRcontig00524:1165101	contig00524	1165101	1166100	1000	4	2.2986E-10	67	6.7	NA	NA
DMRcontig00538:1193101	contig00538	1193101	1193800	700	1	1.8921E-06	30	4.28571429	NA	NA
DMRcontig00546:1210201	contig00546	1210201	1210500	300	1	9.0127E-07	6	2	LOC106461013	Binding Protein
DMRcontig00548:1213265	contig00548	1213265	1214000	736	1	5.6423E-07	19	2.58152174	NA	NA
DMRcontig00637:1402001	contig00637	1402001	1402700	700	1	1.8166E-07	23	3.28571429	NA	NA
DMRcontig00653:1433801	contig00653	1433801	1434298	498	1	2.9333E-08	10	2.00803213	NA	NA
DMRcontig00665:1457401	contig00665	1457401	1458000	600	1	5.3307E-06	18	3	NA	NA
DMRcontig00711:1552501	contig00711	1552501	1552800	300	1	2.3798E-06	13	4.33333333	LOC101857978	Binding Protein
DMRcontig00739:1612801	contig00739	1612801	1613400	600	1	8.6466E-07	5	0.83333333	NA	NA
DMRcontig00763:1662301	contig00763	1662301	1662600	300	2	2.8549E-14	18	6	NA	NA
DMRcontig00767:1670201	contig00767	1670201	1671779	1579	3	4.0129E-08	12	0.75997467	Gm7993;Cnot6l	Transcription;Metabolism
DMRcontig00818:1771101	contig00818	1771101	1771300	200	1	1.2556E-06	10	5	NA	NA
DMRcontig00856:1845867	contig00856	1845867	1846900	1034	2	2.7805E-08	13	1.25725338	NA	NA
DMRcontig00941:2020117	contig00941	2020117	2020400	284	1	2.6886E-06	15	5.28169014	NA	NA
DMRcontig00945:2029201	contig00945	2029201	2029400	200	1	1.6912E-07	5	2.5	NA	NA
DMRcontig00965:2068436	contig00965	2068436	2069300	865	4	1.3421E-17	37	4.27745665	NA	NA
DMRcontig00967:2073001	contig00967	2073001	2073300	300	1	6.058E-08	18	6	NA	NA
DMRcontig00982:2103401	contig00982	2103401	2104400	1000	4	3.6425E-31	23	2.3	zbtb8os	Transcription
DMRcontig01021:2184401	contig01021	2184401	2184699	299	1	5.5611E-06	5	1.6722408	NA	NA
DMRcontig01028:2197413	contig01028	2197413	2198600	1188	2	3.1319E-08	77	6.48148148	NA	NA
DMRcontig01042:2228201	contig01042	2228201	2228900	700	1	1.9951E-06	21	3	NA	NA
DMRcontig01061:2269701	contig01061	2269701	2269900	200	1	3.9274E-06	4	2	NA	NA
DMRcontig01065:2276951	contig01065	2276951	2277900	950	1	6.4114E-06	44	4.63157895	NA	NA
DMRcontig01069:2284864	contig01069	2284864	2285920	1057	6	2.1802E-11	16	1.51371807	COX1;ATP8	Metabolism
DMRcontig01133:2410601	contig01133	2410601	2411000	400	1	6.0888E-07	14	3.5	NA	NA
DMRcontig01150:2445601	contig01150	2445601	2446200	600	1	5.6501E-06	25	4.16666667	LOC105383268;mdh1	Metabolism
DMRcontig01200:2547201	contig01200	2547201	2548700	1500	1	7.9938E-06	41	2.73333333	NA	NA
DMRcontig01212:2573201	contig01212	2573201	2574100	900	1	5.6572E-06	15	1.66666667	LOC106608223	Signaling
DMRcontig01222:2591484	contig01222	2591484	2592300	817	2	6.0827E-11	16	1.95838433	LOC101861190	Signaling
DMRcontig01254:2658101	contig01254	2658101	2658700	600	1	7.5224E-06	17	2.83333333	NA	NA

DMRcontig01287:2722501	contig01287	2722501	2723100	600	2	6.9198E-07	30	5	NA	NA
DMRcontig01308:2763401	contig01308	2763401	2764324	924	4	1.6692E-10	29	3.13852814	NA	NA
DMRcontig01312:2770484	contig01312	2770484	2771500	1017	2	4.532E-08	39	3.83480826	mlnl;NOP56	Unknown;Translation
DMRcontig01314:2775501	contig01314	2775501	2775900	400	2	2.5306E-09	23	5.75	NA	NA
DMRcontig01316:2778901	contig01316	2778901	2779300	400	1	1.8212E-06	10	2.5	Ubl5	Protease
DMRcontig01419:2988901	contig01419	2988901	2989100	200	1	3.2918E-06	17	8.5	NA	NA
DMRcontig01423:2997301	contig01423	2997301	2998300	1000	1	3.9266E-06	55	5.5	NA	NA
DMRcontig01450:3054301	contig01450	3054301	3054500	200	1	4.6494E-07	13	6.5	NA	NA
DMRcontig01453:3059201	contig01453	3059201	3059700	500	3	2.8358E-08	12	2.4	NA	NA
DMRcontig01465:3081615	contig01465	3081615	3082100	486	1	3.7885E-06	9	1.85185185	NA	NA
DMRcontig01469:3088201	contig01469	3088201	3088600	400	1	4.3282E-06	22	5.5	NA	NA
DMRcontig01509:3170901	contig01509	3170901	3171300	400	2	2.1791E-08	10	2.5	NA	NA
DMRcontig01588:3321101	contig01588	3321101	3321400	300	1	8.3071E-06	11	3.66666667	NA	NA
DMRcontig01603:3349012	contig01603	3349012	3349600	589	3	7.9127E-09	18	3.05602716	NA	NA
DMRcontig01607:3356220	contig01607	3356220	3357100	881	1	4.4806E-11	19	2.15664018	NA	NA
DMRcontig01622:3386301	contig01622	3386301	3386500	200	1	5.3293E-06	7	3.5	NA	NA
DMRcontig01649:3437001	contig01649	3437001	3437394	394	3	1.529E-09	20	5.07614213	NA	NA
DMRcontig01674:3486201	contig01674	3486201	3487100	900	1	5.2281E-06	42	4.66666667	NA	NA
DMRcontig01691:3519401	contig01691	3519401	3520200	800	3	2.1732E-08	8	1	NA	NA
DMRcontig01707:3551459	contig01707	3551459	3551700	242	1	2.9955E-08	21	8.67768595	NA	NA
DMRcontig01714:3565501	contig01714	3565501	3566800	1300	2	1.7852E-07	75	5.76923077	NA	NA
DMRcontig01730:3595701	contig01730	3595701	3596000	300	1	4.8401E-06	5	1.66666667	NA	NA
DMRcontig01741:3615569	contig01741	3615569	3616059	491	1	1.0972E-06	12	2.44399185	COX1	Metabolism
DMRcontig01749:3631057	contig01749	3631057	3632000	944	4	2.9643E-31	25	2.64830508	NA	NA
DMRcontig01762:3656901	contig01762	3656901	3657400	500	2	4.8455E-10	10	2	NA	NA
DMRcontig01780:3692502	contig01780	3692502	3692800	299	2	1.8234E-10	4	1.33779264	NA	NA
DMRcontig01790:3712001	contig01790	3712001	3712300	300	2	5.5824E-08	11	3.66666667	NA	NA
DMRcontig01808:3748501	contig01808	3748501	3748967	467	1	8.7014E-06	1	0.21413276	NA	NA
DMRcontig01810:3752801	contig01810	3752801	3753400	600	2	2.8009E-06	12	2	NA	NA
DMRcontig01811:3754701	contig01811	3754701	3756100	1400	1	2.9565E-06	46	3.28571429	NA	NA
DMRcontig01829:3789501	contig01829	3789501	3790600	1100	2	9.9093E-07	31	2.81818182	NA	NA
DMRcontig01848:3828401	contig01848	3828401	3828700	300	1	5.1546E-06	13	4.33333333	NA	NA
DMRcontig01852:3835650	contig01852	3835650	3836100	451	1	1.3628E-06	16	3.54767184	NA	NA
DMRcontig01884:3896601	contig01884	3896601	3897500	900	1	7.3112E-06	17	1.88888889	NA	NA
DMRcontig01918:3961801	contig01918	3961801	3962600	800	2	4.3177E-07	31	3.875	NA	NA
DMRcontig01945:4014201	contig01945	4014201	4014400	200	2	2.941E-06	12	6	Dvir\GJ16291	Unknown
DMRcontig01951:4025601	contig01951	4025601	4025800	200	1	2.4E-07	13	6.5	NA	NA
DMRcontig01976:4075808	contig01976	4075808	4076600	793	2	7.0653E-07	22	2.77427491	NA	NA
DMRcontig01987:4096801	contig01987	4096801	4097454	654	2	1.15E-06	26	3.97553517	NA	NA
DMRcontig01988:4098601	contig01988	4098601	4099200	600	1	4.8043E-12	23	3.83333333	NA	NA
DMRcontig02043:4203953	contig02043	4203953	4204900	948	1	2.3226E-06	39	4.11392405	NA	NA
DMRcontig02044:4206201	contig02044	4206201	4206500	300	1	5.8665E-09	26	8.66666667	NA	NA
DMRcontig02051:4218615	contig02051	4218615	4219100	486	1	5.7075E-10	16	3.29218107	NA	NA
DMRcontig02055:4225706	contig02055	4225706	4226207	502	3	2.2741E-08	8	1.5936255	ATP8;COX1	Metabolism
DMRcontig02101:4315901	contig02101	4315901	4316300	400	1	1.0782E-06	23	5.75	LOC101846824	Growth Factors & Cytokines
DMRcontig02103:4319901	contig02103	4319901	4320800	900	1	2.4426E-06	51	5.66666667	NA	NA
DMRcontig02110:4333849	contig02110	4333849	4335600	1752	9	1.1214E-10	61	3.48173516	NA	NA
DMRcontig02132:4373601	contig02132	4373601	4373900	300	1	2.5908E-06	14	4.66666667	NA	NA
DMRcontig02181:4466301	contig02181	4466301	4466800	500	1	5.7829E-06	17	3.4	NA	NA
DMRcontig02228:4553101	contig02228	4553101	4554000	900	2	1.3828E-08	41	4.55555556	NA	NA
DMRcontig02231:4559201	contig02231	4559201	4559600	400	1	1.8643E-14	18	4.5	NA	NA
DMRcontig02240:4575601	contig02240	4575601	4576353	753	2	1.9535E-08	29	3.85126162	NA	NA
DMRcontig02325:4731758	contig02325	4731758	4732200	443	2	4.2024E-08	11	2.48306998	NA	NA
DMRcontig02340:4760301	contig02340	4760301	4760961	661	4	1.1787E-16	8	1.21028744	COX1	Metabolism
DMRcontig02388:4848001	contig02388	4848001	4849400	1400	1	6.2095E-06	61	4.35714286	NA	NA
DMRcontig02400:4872619	contig02400	4872619	4873643	1025	2	1.4326E-06	29	2.82926829	NA	NA
DMRcontig02412:4894801	contig02412	4894801	4895300	500	2	1.7518E-08	19	3.8	NA	NA
DMRcontig02444:4954601	contig02444	4954601	4954875	275	1	1.4153E-07	13	4.72727273	NA	NA
DMRcontig02446:4957716	contig02446	4957716	4958500	785	2	5.7004E-06	16	2.03821656	NA	NA
DMRcontig02495:5046401	contig02495	5046401	5046700	300	2	9.474E-08	12	4	NA	NA
DMRcontig02513:5075901	contig02513	5075901	5076175	275	2	1.2076E-08	12	4.36363636	VOLCADRAFT_115993	Unknown
DMRcontig02518:5083801	contig02518	5083801	5084000	200	1	3.2096E-06	3	1.5	NA	NA
DMRcontig02545:5133401	contig02545	5133401	5134100	700	1	1.6305E-06	20	2.85714286	NA	NA
DMRcontig02556:5154601	contig02556	5154601	5154900	300	2	1.4198E-06	19	6.33333333	NA	NA
DMRcontig02577:5196901	contig02577	5196901	5197023	123	1	9.01E-06	2	1.62601626	NA	NA
DMRcontig02591:5221901	contig02591	5221901	5222627	727	1	1.2257E-06	48	6.60247593	NA	NA

DMRcontig02661:5353012	contig02661	5353012	5353200	189	1	8.4054E-06	5	2.64550265	NA	NA
DMRcontig02713:5447901	contig02713	5447901	5448800	900	2	9.3308E-09	42	4.66666667	NA	NA
DMRcontig02717:5455017	contig02717	5455017	5455400	384	3	5.1573E-07	12	3.125	NA	NA
DMRcontig02744:5505401	contig02744	5505401	5505500	100	1	2.9926E-08	6	6	NA	NA
DMRcontig02757:5527801	contig02757	5527801	5528500	700	1	4.4025E-07	42	6	LOC105896849	Metabolism
DMRcontig02776:5562423	contig02776	5562423	5562800	378	1	5.7989E-06	9	2.38095238	NA	NA
DMRcontig02779:5569301	contig02779	5569301	5569600	300	1	5.2946E-06	6	2	NA	NA
DMRcontig02799:5605066	contig02799	5605066	5605600	535	2	1.2254E-06	14	2.61682243	NA	NA
DMRcontig02800:5607701	contig02800	5607701	5608000	300	1	4.0469E-07	18	6	NA	NA
DMRcontig02833:5669001	contig02833	5669001	5669400	400	1	4.7375E-06	12	3	NA	NA
DMRcontig02864:5721601	contig02864	5721601	5722100	500	1	1.2863E-06	7	1.4	NA	NA
DMRcontig02880:5751101	contig02880	5751101	5751600	500	1	2.435E-07	15	3	NA	NA
DMRcontig02972:5920656	contig02972	5920656	5921500	845	1	3.4829E-06	21	2.4852071	NA	NA
DMRcontig02979:5933301	contig02979	5933301	5934000	700	2	4.9143E-09	18	2.57142857	NA	NA
DMRcontig02985:5943134	contig02985	5943134	5944200	1067	1	4.2388E-06	59	5.52952202	PTSG_10500	Unknown
DMRcontig02995:5960601	contig02995	5960601	5962000	1400	1	4.4568E-06	28	2	CpipJ_CPIJ013834	Unknown
DMRcontig02999:5968939	contig02999	5968939	5969712	774	1	2.0951E-06	20	2.58397933	NA	NA
DMRcontig03024:6013652	contig03024	6013652	6014100	449	1	4.3873E-06	17	3.78619154	NA	NA
DMRcontig03091:6134601	contig03091	6134601	6135200	600	1	8.0986E-06	9	1.5	NA	NA
DMRcontig03109:6166401	contig03109	6166401	6166800	400	2	5.7308E-06	7	1.75	NA	NA
DMRcontig03158:6257559	contig03158	6257559	6258100	542	1	2.0535E-06	18	3.32103321	NA	NA
DMRcontig03169:6276050	contig03169	6276050	6277000	951	1	3.14E-06	19	1.99789695	NA	NA
DMRcontig03173:6282963	contig03173	6282963	6283300	338	2	1.355E-09	26	7.69230769	NA	NA
DMRcontig03195:6321801	contig03195	6321801	6322300	500	2	1.8639E-06	10	2	NA	NA
DMRcontig03247:6415401	contig03247	6415401	6415900	500	1	1.0514E-09	12	2.4	BRAFLDRAFT_74748	Epigenetic
DMRcontig03283:6481201	contig03283	6481201	6481600	400	1	9.2302E-08	11	2.75	NA	NA
DMRcontig03285:6484701	contig03285	6484701	6485800	1100	3	9.2043E-08	59	5.36363636	LOC100160485	Unknown
DMRcontig03321:6549201	contig03321	6549201	6549489	289	1	5.8781E-07	13	4.4982699	NA	NA
DMRcontig03323:6552101	contig03323	6552101	6552500	400	1	3.4103E-06	3	0.75	NA	NA
DMRcontig03415:6717501	contig03415	6717501	6717800	300	1	1.3727E-08	18	6	NA	NA
DMRcontig03480:6830833	contig03480	6830833	6831435	603	3	1.6428E-07	31	5.14096186	NA	NA
DMRcontig03521:6903601	contig03521	6903601	6903900	300	1	2.0668E-06	8	2.66666667	NA	NA
DMRcontig03524:6909201	contig03524	6909201	6909900	700	1	9.2976E-06	22	3.14285714	NA	NA
DMRcontig03533:6925116	contig03533	6925116	6925400	285	2	1.4618E-06	19	6.66666667	NA	NA
DMRcontig03534:6926901	contig03534	6926901	6927581	681	1	7.6887E-06	18	2.64317181	NA	NA
DMRcontig03555:6965375	contig03555	6965375	6966000	626	1	8.3439E-12	23	3.67412141	NA	NA
DMRcontig03558:6970801	contig03558	6970801	6971200	400	1	6.9209E-06	11	2.75	NA	NA
DMRcontig03574:6999401	contig03574	6999401	7000100	700	1	4.5323E-06	23	3.28571429	Ssc5d	Receptor
DMRcontig03586:7019501	contig03586	7019501	7020000	500	1	7.4663E-06	10	2	NA	NA
DMRcontig03624:7084001	contig03624	7084001	7084388	388	2	8.0458E-20	33	8.50515464	NA	NA
DMRcontig03663:7151701	contig03663	7151701	7152834	1134	2	1.7947E-07	26	2.29276896	NA	NA
DMRcontig03758:7321261	contig03758	7321261	7322000	740	2	6.2855E-07	9	1.21621622	NA	NA
DMRcontig03798:7392701	contig03798	7392701	7393100	400	1	1.885E-06	10	2.5	ETH_00034195	Signaling
DMRcontig03824:7439701	contig03824	7439701	7440000	300	2	1.6707E-13	26	8.66666667	NA	NA
DMRcontig03830:7449901	contig03830	7449901	7450300	400	1	3.0363E-07	7	1.75	NA	NA
DMRcontig03844:7476618	contig03844	7476618	7477200	583	3	8.3517E-12	14	2.40137221	NA	NA
DMRcontig03861:7507401	contig03861	7507401	7507914	514	1	5.9031E-06	21	4.08560311	NA	NA
DMRcontig03871:7523601	contig03871	7523601	7524200	600	1	1.5557E-07	27	4.5	TXNP5	Metabolism
DMRcontig03887:7551659	contig03887	7551659	7552200	542	3	4.2441E-09	31	5.7195572	NA	NA
DMRcontig03911:7593555	contig03911	7593555	7594100	546	1	3.4103E-06	10	1.83150183	NA	NA
DMRcontig03961:7678974	contig03961	7678974	7679200	227	1	4.6671E-06	8	3.52422907	NA	NA
DMRcontig03976:7705535	contig03976	7705535	7705900	366	2	2.4824E-07	11	3.00546448	NA	NA
DMRcontig03981:7713516	contig03981	7713516	7713900	385	1	5.4753E-07	6	1.55844156	NA	NA
DMRcontig04023:7791401	contig04023	7791401	7792500	1100	2	1.4251E-06	50	4.54545455	NA	NA
DMRcontig04025:7795701	contig04025	7795701	7796033	333	1	3.1043E-07	7	2.1021021	NA	NA
DMRcontig04035:7812538	contig04035	7812538	7812900	363	2	4.5153E-12	18	4.95867769	NA	NA
DMRcontig04057:7850468	contig04057	7850468	7850900	433	2	1.5333E-07	9	2.07852194	NA	NA
DMRcontig04064:7862101	contig04064	7862101	7862353	253	1	4.1987E-07	10	3.95256917	NA	NA
DMRcontig04084:7897301	contig04084	7897301	7897663	363	1	2.1217E-07	11	3.03030303	NA	NA
DMRcontig04169:8044660	contig04169	8044660	8044800	141	1	2.2182E-06	9	6.38297872	NA	NA
DMRcontig04172:8049801	contig04172	8049801	8050000	200	1	1.7133E-07	5	2.5	NA	NA
DMRcontig04219:8130901	contig04219	8130901	8131094	194	1	1.6305E-06	9	4.63917526	NA	NA
DMRcontig04261:8202801	contig04261	8202801	8203435	635	2	4.9625E-13	29	4.56692913	NA	NA
DMRcontig04344:8347766	contig04344	8347766	8348092	327	1	1.6374E-06	10	3.05810398	NA	NA
DMRcontig04384:8420167	contig04384	8420167	8420400	234	1	1.1625E-11	5	2.13675214	Gm11675;Gm46346;Myt1l	Metabolism;Unknown;Transcription
DMRcontig04415:8475201	contig04415	8475201	8475816	616	3	9.6021E-08	21	3.40909091	NA	NA

DMRcontig04438:8514101	contig04438	8514101	8514500	400	1	7.223E-06	6	1.5	LOC100777821	Unknown
DMRcontig04498:8619901	contig04498	8619901	8620300	400	1	7.2273E-06	20	5	NA	NA
DMRcontig04523:8664541	contig04523	8664541	8665200	660	1	1.1729E-08	13	1.96969697	NA	NA
DMRcontig04524:8666701	contig04524	8666701	8666900	200	1	4.1883E-07	7	3.5	NA	NA
DMRcontig04530:8675901	contig04530	8675901	8676200	300	1	7.2009E-06	5	1.66666667	NA	NA
DMRcontig04541:8695501	contig04541	8695501	8696430	930	1	3.348E-06	45	4.83870968	NA	NA
DMRcontig04542:8697601	contig04542	8697601	8698089	489	3	4.6094E-09	12	2.45398773	NA	NA
DMRcontig04554:8717113	contig04554	8717113	8717300	188	1	1.1298E-06	12	6.38297872	NA	NA
DMRcontig04621:8831001	contig04621	8831001	8831700	700	5	1.0455E-12	46	6.57142857	NA	NA
DMRcontig04648:8876001	contig04648	8876001	8876400	400	2	1.1311E-09	5	1.25	NA	NA
DMRcontig04658:8893301	contig04658	8893301	8893800	500	2	1.2727E-08	13	2.6	NA	NA
DMRcontig04710:8986501	contig04710	8986501	8986900	400	1	5.4546E-07	29	7.25	NA	NA
DMRcontig04714:8993250	contig04714	8993250	8993700	451	2	1.2644E-08	13	2.88248337	NA	NA
DMRcontig04741:9038773	contig04741	9038773	9039000	228	1	5.6658E-06	1	0.43859649	NA	NA
DMRcontig04752:9058601	contig04752	9058601	9059600	1000	2	8.9331E-08	39	3.9	NA	NA
DMRcontig04769:9090901	contig04769	9090901	9091500	600	2	1.281E-06	19	3.16666667	NA	NA
DMRcontig04783:9115901	contig04783	9115901	9116169	269	1	1.0177E-08	12	4.46096654	NA	NA
DMRcontig04813:9165301	contig04813	9165301	9165500	200	1	3.042E-06	6	3	LOC101757426	Unknown
DMRcontig04862:9247701	contig04862	9247701	9248100	400	1	2.5419E-06	4	1	NA	NA
DMRcontig04934:9369601	contig04934	9369601	9369800	200	1	3.321E-07	7	3.5	NA	NA
DMRcontig04974:9439001	contig04974	9439001	9439576	576	2	3.7133E-11	23	3.99305556	NA	NA
DMRcontig05024:9524101	contig05024	9524101	9524400	300	1	4.3458E-07	6	2	LOC105777419	Unknown
DMRcontig05029:9532001	contig05029	9532001	9532480	480	2	7.7487E-08	13	2.70833333	NA	NA
DMRcontig05036:9542927	contig05036	9542927	9543100	174	1	6.9281E-06	8	4.59770115	NA	NA
DMRcontig05130:9710201	contig05130	9710201	9710400	200	1	3.5743E-06	11	5.5	HPODL_04312	Unknown
DMRcontig05157:9756701	contig05157	9756701	9756900	200	1	1.05E-06	1	0.5	NA	NA
DMRcontig05178:9792108	contig05178	9792108	9792300	193	1	1.0514E-07	13	6.7357513	NA	NA
DMRcontig05181:9797201	contig05181	9797201	9797541	341	1	3.9274E-06	6	1.75953079	NA	NA
DMRcontig05196:9823151	contig05196	9823151	9823600	450	3	1.6459E-06	21	4.66666667	NA	NA
DMRcontig05207:9841701	contig05207	9841701	9842175	475	1	2.084E-07	12	2.52631579	NA	NA
DMRcontig05235:9887262	contig05235	9887262	9888000	739	4	2.8996E-12	12	1.62381597	NA	NA
DMRcontig05242:9900301	contig05242	9900301	9901900	1600	1	3.1955E-06	86	5.375	NA	NA
DMRcontig05252:9917701	contig05252	9917701	9918100	400	1	3.2049E-06	10	2.5	NA	NA
DMRcontig05289:9979801	contig05289	9979801	9980272	472	2	1.6538E-06	7	1.48305085	NA	NA
DMRcontig05329:10049201	contig05329	10049201	10049500	300	1	7.0206E-06	8	2.66666667	NA	NA
DMRcontig05357:10096701	contig05357	10096701	10097100	400	1	7.8137E-14	19	4.75	NA	NA
DMRcontig05360:10101201	contig05360	10101201	10101571	371	1	1.1983E-08	8	2.15633423	NA	NA
DMRcontig05494:10326424	contig05494	10326424	10326700	277	1	4.7471E-06	6	2.16606498	NA	NA
DMRcontig05519:10370203	contig05519	10370203	10370800	598	2	5.1542E-07	29	4.84949833	NA	NA
DMRcontig05532:10391201	contig05532	10391201	10391500	300	2	1.2927E-06	12	4	NA	NA
DMRcontig05534:10394905	contig05534	10394905	10395846	942	1	2.0716E-06	41	4.35244161	LOC106609801;LOC106609812;LOC106595659;LOC106596913;yeats4	Unknown;Transcription
DMRcontig05567:10452319	contig05567	10452319	10453155	837	1	5.802E-08	21	2.50896057	NA	NA
DMRcontig05596:10501001	contig05596	10501001	10501329	329	2	1.9227E-09	6	1.82370821	NA	NA
DMRcontig05614:10530701	contig05614	10530701	10531500	800	1	9.3053E-07	23	2.875	mib1.L	Protease
DMRcontig05622:10543901	contig05622	10543901	10544300	400	1	2.3249E-06	5	1.25	NA	NA
DMRcontig05623:10545701	contig05623	10545701	10546115	415	1	9.0723E-07	7	1.68674699	NA	NA
DMRcontig05703:10679901	contig05703	10679901	10680100	200	1	3.2096E-06	6	3	NA	NA
DMRcontig05789:10824488	contig05789	10824488	10824700	213	2	7.7936E-10	11	5.16431925	NA	NA
DMRcontig05822:10880201	contig05822	10880201	10880500	300	1	3.6573E-08	6	2	NA	NA
DMRcontig05826:10887201	contig05826	10887201	10887520	320	2	6.0041E-10	9	2.8125	NA	NA
DMRcontig05862:10949401	contig05862	10949401	10949900	500	1	5.4546E-07	17	3.4	NA	NA
DMRcontig06076:11308001	contig06076	11308001	11308400	400	1	7.3759E-07	12	3	NA	NA
DMRcontig06095:11338417	contig06095	11338417	11339000	584	3	1.179E-09	15	2.56849315	NA	NA
DMRcontig06133:11402379	contig06133	11402379	11403013	635	1	5.613E-07	11	1.73228346	NA	NA
DMRcontig06147:11427101	contig06147	11427101	11427300	200	1	6.3308E-06	9	4.5	LOC101850127;psmc2	Protease;Metabolism
DMRcontig06163:11453698	contig06163	11453698	11454928	1231	2	9.1974E-09	21	1.70593014	NA	NA
DMRcontig06167:11461501	contig06167	11461501	11461700	200	1	3.6333E-08	11	5.5	NA	NA
DMRcontig06187:11495401	contig06187	11495401	11495900	500	1	3.2266E-06	14	2.8	NA	NA
DMRcontig06250:11598437	contig06250	11598437	11598700	264	2	4.4832E-07	19	7.1969697	NA	NA
DMRcontig06277:11644101	contig06277	11644101	11644464	364	1	3.04E-06	4	1.0989011	NA	NA
DMRcontig06377:11812825	contig06377	11812825	11813600	776	1	7.3868E-06	19	2.44845361	NA	NA
DMRcontig06412:11867764	contig06412	11867764	11868100	337	1	1.4266E-08	1	0.29673591	NA	NA
DMRcontig06441:11916701	contig06441	11916701	11917097	397	1	3.3278E-06	8	2.01511335	NA	NA
DMRcontig06442:11918301	contig06442	11918301	11918600	300	2	4.8728E-12	10	3.33333333	NA	NA
DMRcontig06475:11973601	contig06475	11973601	11973800	200	2	1.5802E-09	21	10.5	NA	NA

DMRcontig06481:11983201	contig06481	11983201	11983970	770	1	1.4664E-06	12	1.55844156	NA	NA
DMRcontig06497:12010504	contig06497	12010504	12011300	797	2	7.6561E-07	16	2.00752823	NA	NA
DMRcontig06504:12021865	contig06504	12021865	12022300	436	2	5.2638E-08	31	7.11009174	NA	NA
DMRcontig06538:12079176	contig06538	12079176	12079658	483	1	7.3203E-06	28	5.79710145	NA	NA
DMRcontig06553:12103501	contig06553	12103501	12103800	300	2	9.8417E-07	15	5	NA	NA
DMRcontig06572:12134501	contig06572	12134501	12134999	499	1	5.1778E-07	16	3.20641283	NA	NA
DMRcontig06573:12136401	contig06573	12136401	12136800	400	1	7.1273E-06	8	2	NA	NA
DMRcontig06591:12164401	contig06591	12164401	12164900	500	2	3.6834E-15	23	4.6	NA	NA
DMRcontig06599:12178601	contig06599	12178601	12178900	300	1	1.4997E-07	11	3.66666667	NA	NA
DMRcontig06611:12198101	contig06611	12198101	12198300	200	1	2.989E-06	10	5	NA	NA
DMRcontig06626:12221701	contig06626	12221701	12222100	400	1	4.3191E-08	12	3	LOC105398638;hspa5	Protein Binding;Unknown
DMRcontig06666:12286901	contig06666	12286901	12287471	571	5	1.2E-09	26	4.55341506	NA	NA
DMRcontig06713:12364401	contig06713	12364401	12364900	500	1	9.1849E-09	19	3.8	NA	NA
DMRcontig06740:12408491	contig06740	12408491	12408900	410	2	1.2978E-06	15	3.65853659	NA	NA
DMRcontig06769:12454901	contig06769	12454901	12455300	400	1	2.6438E-07	13	3.25	NA	NA
DMRcontig06775:12464375	contig06775	12464375	12465261	887	1	1.4733E-09	50	5.63697858	NA	NA
DMRcontig06804:12512432	contig06804	12512432	12512700	269	2	8.3019E-10	8	2.9739777	NA	NA
DMRcontig06845:12579601	contig06845	12579601	12579900	300	2	5.5738E-14	22	7.33333333	NA	NA
DMRcontig06853:12592901	contig06853	12592901	12593200	300	1	5.9043E-07	3	1	SNOG_05957	Unknown
DMRcontig06859:12602301	contig06859	12602301	12602736	436	2	1.8978E-07	11	2.52293578	Kpol_1060p35	Unknown
DMRcontig06945:12742926	contig06945	12742926	12743413	488	1	4.0301E-06	3	0.6147541	NA	NA
DMRcontig07016:12860001	contig07016	12860001	12860400	400	1	8.716E-07	20	5	NA	NA
DMRcontig07064:12938301	contig07064	12938301	12938500	200	1	4.5139E-06	8	4	NA	NA
DMRcontig07066:12941401	contig07066	12941401	12941700	300	1	2.7385E-06	2	0.66666667	LOC106163552	Metabolism
DMRcontig07101:12999901	contig07101	12999901	13000300	400	1	4.0853E-06	16	4	NA	NA
DMRcontig07108:13010801	contig07108	13010801	13011693	893	1	1.5958E-06	20	2.23964166	NA	NA
DMRcontig07131:13048801	contig07131	13048801	13049200	400	1	5.4052E-06	29	7.25	NA	NA
DMRcontig07149:13078001	contig07149	13078001	13078300	300	2	4.0275E-16	4	1.33333333	NA	NA
DMRcontig07172:13114801	contig07172	13114801	13115261	461	2	3.2337E-09	7	1.51843818	NA	NA
DMRcontig07182:13130471	contig07182	13130471	13131107	637	1	7.4253E-06	21	3.2967033	NA	NA
DMRcontig07187:13138601	contig07187	13138601	13139400	800	8	1.6977E-17	31	3.875	NA	NA
DMRcontig07221:13193423	contig07221	13193423	13193800	378	1	8.6059E-07	8	2.11640212	NA	NA
DMRcontig07318:13348701	contig07318	13348701	13349558	858	2	5.5677E-07	16	1.86480186	NA	NA
DMRcontig07348:13395401	contig07348	13395401	13395700	300	1	1.6044E-06	12	4	NA	NA
DMRcontig07350:13398901	contig07350	13398901	13399121	221	1	2.9239E-06	9	4.07239819	NA	NA
DMRcontig07351:13400501	contig07351	13400501	13401100	600	1	4.4933E-07	18	3	NA	NA
DMRcontig07397:13474326	contig07397	13474326	13474747	422	3	3.3467E-14	29	6.87203791	NA	NA
DMRcontig07413:13500201	contig07413	13500201	13500700	500	1	7.6578E-06	16	3.2	NA	NA
DMRcontig07459:13573201	contig07459	13573201	13574097	897	1	3.7162E-08	20	2.2296544	NA	NA
DMRcontig07495:13629401	contig07495	13629401	13629700	300	1	6.2855E-07	4	1.33333333	NA	NA
DMRcontig07544:13709301	contig07544	13709301	13709600	300	1	2.0162E-07	13	4.33333333	NA	NA
DMRcontig07547:13714101	contig07547	13714101	13714500	400	1	7.8811E-07	11	2.75	NA	NA
DMRcontig07566:13744301	contig07566	13744301	13744700	400	1	5.9747E-06	22	5.5	ACTG1	Cytoskeleton
DMRcontig07587:13778901	contig07587	13778901	13779100	200	1	5.9832E-08	3	1.5	NA	NA
DMRcontig07612:13821111	contig07612	13821111	13821500	390	1	8.8428E-07	6	1.53846154	NA	NA
DMRcontig07626:13843301	contig07626	13843301	13843673	373	1	3.7894E-06	4	1.07238606	NA	NA
DMRcontig07643:13869501	contig07643	13869501	13869878	378	1	1.8049E-06	13	3.43915344	NA	NA
DMRcontig07680:13931801	contig07680	13931801	13932700	900	2	1.1177E-06	12	1.33333333	NA	NA
DMRcontig07684:13938301	contig07684	13938301	13939100	800	2	2.4506E-06	27	3.375	NA	NA
DMRcontig07711:13982501	contig07711	13982501	13982988	488	1	2.0994E-08	4	0.81967213	NA	NA
DMRcontig07714:13987701	contig07714	13987701	13988900	1200	4	5.4704E-13	52	4.33333333	NA	NA
DMRcontig07797:14123101	contig07797	14123101	14123700	600	2	3.412E-06	21	3.5	NA	NA
DMRcontig07825:14169297	contig07825	14169297	14170000	704	3	5.2199E-11	15	2.13068182	COX1;ND3	Metabolism
DMRcontig07833:14182201	contig07833	14182201	14182800	600	2	4.7785E-14	18	3	NA	NA
DMRcontig07851:14211201	contig07851	14211201	14211700	500	1	1.8958E-06	15	3	NA	NA
DMRcontig07854:14215801	contig07854	14215801	14216000	200	1	8.4191E-09	13	6.5	NA	NA
DMRcontig07922:14324901	contig07922	14324901	14325300	400	2	1.0878E-07	23	5.75	NA	NA
DMRcontig07953:14374501	contig07953	14374501	14374900	400	1	7.6225E-06	11	2.75	NA	NA
DMRcontig07970:14403201	contig07970	14403201	14403575	375	1	5.3777E-08	6	1.6	NA	NA
DMRcontig08027:14495201	contig08027	14495201	14495600	400	2	1.138E-13	37	9.25	NA	NA
DMRcontig08050:14532485	contig08050	14532485	14532900	416	1	1.0093E-07	12	2.88461538	NA	NA
DMRcontig08053:14537701	contig08053	14537701	14538000	300	2	9.4502E-07	4	1.33333333	NA	NA
DMRcontig08098:14609101	contig08098	14609101	14609800	700	1	5.5888E-09	29	4.14285714	NA	NA
DMRcontig08191:14757501	contig08191	14757501	14758000	500	1	1.5419E-06	21	4.2	NA	NA
DMRcontig08206:14781330	contig08206	14781330	14781600	271	2	9.1326E-14	12	4.42804428	NA	NA
DMRcontig08217:14799801	contig08217	14799801	14800037	237	1	1.704E-06	7	2.9535865	NA	NA
DMRcontig08238:14833601	contig08238	14833601	14834200	600	1	7.6972E-07	6	1	NA	NA

DMRcontig08350:15015801	contig08350	15015801	15016091	291	2	6.0823E-12	5	1.71821306	NA	NA
DMRcontig08357:15026601	contig08357	15026601	15026900	300	1	8.075E-07	12	4	NA	NA
DMRcontig08377:15058595	contig08377	15058595	15059700	1106	5	1.029E-11	26	2.35081374	NA	NA
DMRcontig08412:15114788	contig08412	15114788	15115240	453	3	2.3582E-11	11	2.42825607	NA	NA
DMRcontig08446:15171250	contig08446	15171250	15171600	351	1	1.1209E-06	3	0.85470085	NA	NA
DMRcontig08460:15193601	contig08460	15193601	15193800	200	1	1.172E-06	6	3	NA	NA
DMRcontig08471:15210630	contig08471	15210630	15211100	471	1	1.2547E-06	13	2.76008493	NA	NA
DMRcontig08515:15278503	contig08515	15278503	15278800	298	1	1.0706E-06	22	7.38255034	NA	NA
DMRcontig08559:15348353	contig08559	15348353	15348600	248	2	1.8405E-11	8	3.22580645	NA	NA
DMRcontig08588:15396201	contig08588	15396201	15397031	831	3	1.2413E-09	27	3.24909747	NA	NA
DMRcontig08656:15505333	contig08656	15505333	15505831	499	2	2.6218E-07	5	1.00200401	NA	NA
DMRcontig08669:15525816	contig08669	15525816	15526900	1085	1	3.5518E-06	11	1.01382488	NA	NA
DMRcontig08729:15621501	contig08729	15621501	15622000	500	1	2.0992E-09	21	4.2	NA	NA
DMRcontig08783:15707169	contig08783	15707169	15708587	1419	1	2.8303E-06	38	2.67794221	NA	NA
DMRcontig08824:15771001	contig08824	15771001	15771398	398	2	1.9331E-07	10	2.51256281	NA	NA
DMRcontig08841:15797047	contig08841	15797047	15797700	654	1	4.3605E-06	19	2.90519878	NA	NA
DMRcontig08867:15837742	contig08867	15837742	15838553	812	2	5.6114E-07	24	2.95566502	NA	NA
DMRcontig08913:15910601	contig08913	15910601	15911279	679	4	1.8667E-08	15	2.20913108	NA	NA
DMRcontig08953:15975201	contig08953	15975201	15975500	300	2	1.8192E-06	9	3	NA	NA
DMRcontig08979:16015824	contig08979	16015824	16016338	515	1	6.9228E-06	5	0.97087379	NA	NA
DMRcontig09001:16049801	contig09001	16049801	16050200	400	1	7.3477E-07	11	2.75	NA	NA
DMRcontig09036:16105801	contig09036	16105801	16106700	900	1	6.142E-07	9	1	NA	NA
DMRcontig09055:16135341	contig09055	16135341	16135800	460	3	1.5716E-07	5	1.08695652	NA	NA
DMRcontig09105:16212901	contig09105	16212901	16213000	100	1	2.5667E-06	1	1	NA	NA
DMRcontig09129:16252401	contig09129	16252401	16252900	500	4	5.0239E-28	33	6.6	CpipJ_CPIJ017745;FAXDC2	Metabolism
DMRcontig09205:16373301	contig09205	16373301	16373882	582	1	5.3356E-10	29	4.98281787	NA	NA
DMRcontig09219:16395701	contig09219	16395701	16396000	300	2	1.0177E-12	15	5	NA	NA
DMRcontig09266:16467201	contig09266	16467201	16467586	386	2	8.075E-07	2	0.51813472	NA	NA
DMRcontig09328:16564901	contig09328	16564901	16565000	100	1	4.7824E-07	4	4	NA	NA
DMRcontig09342:16587114	contig09342	16587114	16587600	487	1	6.8044E-06	7	1.43737166	NA	NA
DMRcontig09416:16705601	contig09416	16705601	16705900	300	1	2.5488E-06	11	3.66666667	NA	NA
DMRcontig09450:16759001	contig09450	16759001	16759425	425	1	5.8168E-06	12	2.82352941	NA	NA
DMRcontig09451:16760426	contig09451	16760426	16760600	175	1	5.5885E-06	8	4.57142857	NA	NA
DMRcontig09547:16909669	contig09547	16909669	16910245	577	1	5.1919E-06	28	4.85268631	NA	NA
DMRcontig09568:16942701	contig09568	16942701	16943000	300	1	8.6463E-06	6	2	NA	NA
DMRcontig09600:16994001	contig09600	16994001	16994400	400	1	5.5729E-06	16	4	NA	NA
DMRcontig09622:17028801	contig09622	17028801	17029000	200	1	2.8447E-06	2	1	NA	NA
DMRcontig09628:17038001	contig09628	17038001	17038400	400	1	2.435E-06	12	3	NA	NA
DMRcontig09634:17048001	contig09634	17048001	17048400	400	4	6.1881E-08	22	5.5	NA	NA
DMRcontig09685:17126987	contig09685	17126987	17127500	514	3	7.5804E-13	18	3.50194553	NA	NA
DMRcontig09711:17168691	contig09711	17168691	17168938	248	1	1.5796E-06	9	3.62903226	NA	NA
DMRcontig09742:17217701	contig09742	17217701	17217993	293	1	9.0615E-06	6	2.04778157	NA	NA
DMRcontig09835:17363701	contig09835	17363701	17364100	400	1	4.8569E-09	9	2.25	NA	NA
DMRcontig09837:17366676	contig09837	17366676	17367000	325	1	6.6473E-06	2	0.61538462	NA	NA
DMRcontig09843:17376301	contig09843	17376301	17376900	600	1	3.908E-07	16	2.66666667	NA	NA
DMRcontig09860:17402859	contig09860	17402859	17403288	430	3	3.3754E-07	18	4.18604651	NA	NA
DMRcontig09884:17437701	contig09884	17437701	17437800	100	1	1.7939E-06	6	6	NA	NA
DMRcontig09899:17460248	contig09899	17460248	17460730	483	3	2.1152E-10	9	1.86335404	NA	NA
DMRcontig09949:17535917	contig09949	17535917	17536564	648	1	9.9662E-06	14	2.16049383	NA	NA
DMRcontig09971:17570501	contig09971	17570501	17571000	500	1	1.1114E-08	8	1.6	NA	NA
DMRcontig09993:17606224	contig09993	17606224	17606764	541	2	1.7918E-07	45	8.31792976	NA	NA
DMRcontig10021:17649301	contig10021	17649301	17650400	1100	1	4.8611E-06	22	2	NA	NA
DMRcontig10024:17654354	contig10024	17654354	17654500	147	1	2.5318E-07	6	4.08163265	NA	NA
DMRcontig10060:17711701	contig10060	17711701	17712185	485	2	1.3267E-06	18	3.71134021	NA	NA
DMRcontig10093:17761801	contig10093	17761801	17762290	490	3	4.6919E-08	6	1.2244898	NA	NA
DMRcontig10191:17914201	contig10191	17914201	17914900	700	1	8.811E-06	36	5.14285714	NA	NA
DMRcontig10208:17940478	contig10208	17940478	17940874	397	3	2.4001E-07	17	4.28211587	NA	NA
DMRcontig10237:17985401	contig10237	17985401	17985586	186	1	3.8021E-07	0	0	NA	NA
DMRcontig10250:18004901	contig10250	18004901	18005300	400	2	2.0063E-06	17	4.25	NA	NA
DMRcontig10293:18070601	contig10293	18070601	18071727	1127	7	1.1673E-17	40	3.54924579	NA	NA
DMRcontig10295:18074101	contig10295	18074101	18074200	100	1	1.3804E-08	3	3	NA	NA
DMRcontig10362:18175801	contig10362	18175801	18176300	500	1	9.5169E-08	14	2.8	NA	NA
DMRcontig10366:18181701	contig10366	18181701	18182000	300	1	4.8034E-06	16	5.33333333	NA	NA
DMRcontig10462:18328101	contig10462	18328101	18328400	300	1	8.9724E-08	7	2.33333333	NA	NA
DMRcontig10480:18355388	contig10480	18355388	18355700	313	1	9.3193E-06	28	8.9456869	NA	NA
DMRcontig10533:18435301	contig10533	18435301	18435600	300	1	3.7332E-06	26	8.66666667	NA	NA

DMRcontig10540:18446701	contig10540	18446701	18447100	400	2	1.049E-08	14	3.5	NA	NA
DMRcontig10580:18508446	contig10580	18508446	18508800	355	2	3.8739E-07	5	1.4084507	NA	NA
DMRcontig10725:18733201	contig10725	18733201	18734000	800	1	6.8717E-06	44	5.5	NA	NA
DMRcontig10768:18799688	contig10768	18799688	18800200	513	1	1.2968E-06	9	1.75438596	LOC109063407	Unknown
DMRcontig10807:18860301	contig10807	18860301	18860643	343	1	6.0289E-08	6	1.74927114	NA	NA
DMRcontig10811:18865901	contig10811	18865901	18866300	400	1	2.0482E-06	16	4	NA	NA
DMRcontig10822:18881321	contig10822	18881321	18881754	434	1	9.4505E-07	12	2.76497696	NA	NA
DMRcontig10834:18900001	contig10834	18900001	18900799	799	1	1.9793E-08	36	4.50563204	NA	NA
DMRcontig10848:18921316	contig10848	18921316	18922000	685	1	4.3935E-12	20	2.91970803	NA	NA
DMRcontig10874:18960901	contig10874	18960901	18961300	400	1	9.6127E-10	26	6.5	NA	NA
DMRcontig10884:18975932	contig10884	18975932	18976400	469	2	4.8851E-06	5	1.06609808	NA	NA
DMRcontig10893:18989501	contig10893	18989501	18989900	400	2	1.9221E-07	9	2.25	NA	NA
DMRcontig10931:19047401	contig10931	19047401	19047700	300	2	1.111E-08	2	0.66666667	NA	NA
DMRcontig10987:19132202	contig10987	19132202	19132900	699	1	2.8447E-06	7	1.00143062	NA	NA
DMRcontig10995:19144501	contig10995	19144501	19144700	200	1	6.7203E-08	5	2.5	NA	NA
DMRcontig11037:19209501	contig11037	19209501	19209700	200	1	1.5665E-06	4	2	NA	NA
DMRcontig11155:19386368	contig11155	19386368	19386700	333	1	1.0068E-10	4	1.2012012	NA	NA
DMRcontig11184:19429501	contig11184	19429501	19430000	500	2	3.4984E-06	15	3	NA	NA
DMRcontig11187:19434201	contig11187	19434201	19434600	400	1	2.4426E-06	11	2.75	NA	NA
DMRcontig11248:19527840	contig11248	19527840	19528100	261	1	2.1301E-06	3	1.14942529	NA	NA
DMRcontig11262:19549401	contig11262	19549401	19549800	400	1	9.423E-06	13	3.25	NA	NA
DMRcontig11303:19611701	contig11303	19611701	19612000	300	1	7.0961E-06	6	2	NA	NA
DMRcontig11306:19616001	contig11306	19616001	19616400	400	1	1.4461E-07	5	1.25	NA	NA
DMRcontig11350:19681308	contig11350	19681308	19681800	493	2	1.0416E-07	10	2.02839757	NA	NA
DMRcontig11431:19802501	contig11431	19802501	19802800	300	2	8.2677E-08	7	2.33333333	NA	NA
DMRcontig11459:19845817	contig11459	19845817	19846059	243	2	3.2111E-10	5	2.05761317	NA	NA
DMRcontig11518:19936701	contig11518	19936701	19936900	200	1	8.4072E-06	8	4	NA	NA
DMRcontig11521:19940901	contig11521	19940901	19941199	299	1	1.026E-07	16	5.35117057	NA	NA
DMRcontig11529:19952401	contig11529	19952401	19952800	400	1	4.8991E-06	25	6.25	NA	NA
DMRcontig11534:19960001	contig11534	19960001	19960500	500	3	1.299E-09	27	5.4	NA	NA
DMRcontig11547:19979156	contig11547	19979156	19979600	445	3	6.948E-07	16	3.59550562	NA	NA
DMRcontig11557:19994201	contig11557	19994201	19994500	300	1	1.0339E-06	16	5.33333333	NA	NA
DMRcontig11593:20046749	contig11593	20046749	20047184	436	5	1.8101E-21	13	2.98165138	NA	NA
DMRcontig11622:20088329	contig11622	20088329	20088831	503	4	3.6575E-13	17	3.37972167	NA	NA
DMRcontig11695:20198570	contig11695	20198570	20199196	627	5	2.2129E-20	17	2.71132376	NA	NA
DMRcontig11731:20254201	contig11731	20254201	20254700	500	1	1.9526E-06	34	6.8	NA	NA
DMRcontig11854:20440637	contig11854	20440637	20440800	164	1	2.7625E-06	6	3.65853659	NA	NA
DMRcontig11987:20639101	contig11987	20639101	20639400	300	1	4.6824E-09	10	3.33333333	NA	NA
DMRcontig12021:20689621	contig12021	20689621	20689900	280	1	8.3805E-06	10	3.57142857	NA	NA
DMRcontig12042:20723404	contig12042	20723404	20723700	297	2	5.7299E-12	5	1.68350168	NA	NA
DMRcontig12051:20736801	contig12051	20736801	20737100	300	1	7.7244E-06	12	4	NA	NA
DMRcontig12059:20749001	contig12059	20749001	20749400	400	1	8.6145E-07	5	1.25	NA	NA
DMRcontig12082:20783601	contig12082	20783601	20783800	200	1	2.6142E-10	11	5.5	NA	NA
DMRcontig12100:20810439	contig12100	20810439	20810700	262	1	1.6081E-07	6	2.29007634	NA	NA
DMRcontig12123:20845958	contig12123	20845958	20846400	443	2	2.4049E-08	6	1.35440181	NA	NA
DMRcontig12185:20937701	contig12185	20937701	20938132	432	1	2.5188E-07	10	2.31481481	NA	NA
DMRcontig12215:20982931	contig12215	20982931	20983600	670	3	1.8953E-09	11	1.64179104	NA	NA
DMRcontig12259:21050701	contig12259	21050701	21051086	386	1	2.4753E-07	18	4.66321244	NA	NA
DMRcontig12278:21078801	contig12278	21078801	21078900	100	1	5.5885E-06	11	11	NA	NA
DMRcontig12284:21087301	contig12284	21087301	21087700	400	1	6.573E-06	17	4.25	NA	NA
DMRcontig12397:21260301	contig12397	21260301	21260600	300	1	5.3175E-08	2	0.66666667	NA	NA
DMRcontig12404:21270601	contig12404	21270601	21270800	200	1	8.4999E-09	1	0.5	NA	NA
DMRcontig12426:21303145	contig12426	21303145	21303700	556	1	4.0767E-06	12	2.15827338	NA	NA
DMRcontig12511:21433601	contig12511	21433601	21433795	195	1	7.4611E-06	8	4.1025641	NA	NA
DMRcontig12514:21437501	contig12514	21437501	21437900	400	1	3.4359E-06	16	4	NA	NA
DMRcontig12522:21449466	contig12522	21449466	21449600	135	1	2.7617E-06	1	0.74074074	NA	NA
DMRcontig12524:21452301	contig12524	21452301	21452500	200	1	2.9773E-07	11	5.5	NA	NA
DMRcontig12541:21478196	contig12541	21478196	21478500	305	1	6.2573E-06	11	3.60655738	NA	NA
DMRcontig12599:21564625	contig12599	21564625	21564933	309	1	4.453E-06	6	1.94174757	NA	NA
DMRcontig12653:21643901	contig12653	21643901	21644184	284	1	9.2042E-10	6	2.11267606	NA	NA
DMRcontig12669:21667201	contig12669	21667201	21667500	300	1	1.6305E-06	9	3	NA	NA
DMRcontig12690:21698851	contig12690	21698851	21699100	250	1	3.7849E-07	3	1.2	NA	NA
DMRcontig12858:21946022	contig12858	21946022	21946300	279	1	3.5397E-06	5	1.7921147	NA	NA
DMRcontig12867:21959635	contig12867	21959635	21960141	507	1	9.7512E-06	17	3.3530572	NA	NA
DMRcontig12941:22069589	contig12941	22069589	22069900	312	1	9.226E-06	10	3.20512821	NA	NA
DMRcontig12960:22097167	contig12960	22097167	22097376	210	1	1.4397E-10	3	1.42857143	NA	NA
DMRcontig13089:22287101	contig13089	22287101	22287700	600	3	1.004E-07	36	6	NA	NA

DMRcontig13121:22334801	contig13121	22334801	22335300	500	3	1.4322E-16	34	6.8	NA	NA
DMRcontig13124:22339638	contig13124	22339638	22339883	246	1	1.6609E-09	8	3.25203252	NA	NA
DMRcontig13141:22365643	contig13141	22365643	22366200	558	3	1.1978E-11	20	3.58422939	NA	NA
DMRcontig13192:22440008	contig13192	22440008	22440235	228	1	8.2795E-06	7	3.07017544	NA	NA
DMRcontig13259:22536401	contig13259	22536401	22536800	400	1	5.6368E-07	11	2.75	LOC101858697	Unknown
DMRcontig13269:22552139	contig13269	22552139	22552400	262	1	7.0506E-06	7	2.67175573	NA	NA
DMRcontig13288:22581032	contig13288	22581032	22581517	486	5	1.5988E-10	25	5.14403292	NA	NA
DMRcontig13291:22585401	contig13291	22585401	22585900	500	3	6.3186E-10	16	3.2	NA	NA
DMRcontig13311:22615301	contig13311	22615301	22615600	300	1	6.1765E-06	11	3.66666667	NA	NA
DMRcontig13317:22624701	contig13317	22624701	22624800	100	1	4.2831E-07	3	3	NA	NA
DMRcontig13467:22847979	contig13467	22847979	22848200	222	2	1.1594E-06	8	3.6036036	NA	NA
DMRcontig13498:22893401	contig13498	22893401	22893800	400	1	8.6779E-06	27	6.75	NA	NA
DMRcontig13522:22929701	contig13522	22929701	22930100	400	2	1.3636E-08	5	1.25	NA	NA
DMRcontig13527:22937007	contig13527	22937007	22937200	194	1	9.4485E-08	0	0	NA	NA
DMRcontig13534:22948001	contig13534	22948001	22948342	342	1	5.7062E-06	11	3.21637427	NA	NA
DMRcontig13696:23192901	contig13696	23192901	23193122	222	1	6.8267E-06	4	1.8018018	NA	NA
DMRcontig13704:23205001	contig13704	23205001	23205200	200	1	8.075E-07	4	2	NA	NA
DMRcontig13737:23253031	contig13737	23253031	23253500	470	1	2.9747E-06	20	4.25531915	NA	NA
DMRcontig13756:23280401	contig13756	23280401	23280800	400	2	2.4772E-10	16	4	NA	NA
DMRcontig13830:23392301	contig13830	23392301	23392459	159	1	8.9827E-07	4	2.51572327	NA	NA
DMRcontig13915:23518040	contig13915	23518040	23518200	161	1	3.6129E-06	8	4.9689441	NA	NA
DMRcontig13936:23548143	contig13936	23548143	23548900	758	1	4.0413E-08	16	2.11081794	LOC107987209;LOC107984688;LOC108168064;LOC107973554;HOXD10;Spon1;PDE1B	Transcription;Cytoskeleton;Unknown
DMRcontig13944:23561101	contig13944	23561101	23561400	300	1	1.5732E-07	7	2.33333333	NA	NA
DMRcontig13997:23640501	contig13997	23640501	23640776	276	1	4.2801E-06	10	3.62318841	NA	NA
DMRcontig14064:23738204	contig14064	23738204	23738500	297	2	3.7696E-09	11	3.7037037	NA	NA
DMRcontig14218:23965716	contig14218	23965716	23966091	376	3	4.0835E-14	3	0.79787234	NA	NA
DMRcontig14248:24009901	contig14248	24009901	24010400	500	1	3.823E-06	21	4.2	NA	NA
DMRcontig14318:24111208	contig14318	24111208	24111800	593	2	1.0782E-06	39	6.5767285	THITE_2119671;THITE_2054382	Metabolism;Unknown
DMRcontig14337:24139701	contig14337	24139701	24139900	200	1	2.2824E-06	9	4.5	NA	NA
DMRcontig14382:24205923	contig14382	24205923	24206350	428	1	1.3312E-06	16	3.73831776	NA	NA
DMRcontig14400:24232760	contig14400	24232760	24233100	341	1	9.2121E-06	3	0.8797654	NA	NA
DMRcontig14529:24424540	contig14529	24424540	24424900	361	1	7.3993E-07	7	1.93905817	NA	NA
DMRcontig14531:24427601	contig14531	24427601	24427900	300	1	6.6633E-06	9	3	NA	NA
DMRcontig14543:24444801	contig14543	24444801	24445046	246	1	2.0766E-06	4	1.62601626	NA	NA
DMRcontig14547:24450363	contig14547	24450363	24450700	338	2	3.1602E-06	7	2.07100592	NA	NA
DMRcontig14597:24524501	contig14597	24524501	24524948	448	3	4.6669E-07	9	2.00892857	NA	NA
DMRcontig14601:24530255	contig14601	24530255	24530600	346	2	9.0789E-07	5	1.44508671	NA	NA
DMRcontig14615:24551401	contig14615	24551401	24551600	200	1	2.5153E-07	2	1	NA	NA
DMRcontig14618:24555801	contig14618	24555801	24556070	270	3	1.1774E-07	5	1.85185185	NA	NA
DMRcontig14619:24557301	contig14619	24557301	24557700	400	2	1.6818E-06	13	3.25	NA	NA
DMRcontig14671:24632667	contig14671	24632667	24633000	334	1	5.6812E-07	2	0.5988024	NA	NA
DMRcontig14684:24651945	contig14684	24651945	24652300	356	1	3.0833E-07	1	0.28089888	NA	NA
DMRcontig14742:24737201	contig14742	24737201	24737542	342	1	5.0415E-06	4	1.16959064	NA	NA
DMRcontig14763:24770644	contig14763	24770644	24770900	257	2	1.5415E-10	9	3.50194553	NA	NA
DMRcontig14799:24823401	contig14799	24823401	24823700	300	1	6.99E-06	5	1.66666667	NA	NA
DMRcontig14821:24856013	contig14821	24856013	24856356	344	2	1.2047E-06	5	1.45348837	NA	NA
DMRcontig14862:24917822	contig14862	24917822	24918519	698	1	3.498E-06	43	6.16045845	NA	NA
DMRcontig14953:25051602	contig14953	25051602	25051865	264	2	2.7119E-10	13	4.92424242	NA	NA
DMRcontig14959:25060189	contig14959	25060189	25060400	212	1	5.6662E-06	3	1.41509434	NA	NA
DMRcontig14960:25061601	contig14960	25061601	25061800	200	1	1.9458E-06	4	2	LOC101415577;EFR3B	Development;Unknown
DMRcontig14971:25077609	contig14971	25077609	25078000	392	2	5.7445E-10	5	1.2755102	NA	NA
DMRcontig14999:25118501	contig14999	25118501	25118700	200	1	5.3242E-06	10	5	NA	NA
DMRcontig15021:25150322	contig15021	25150322	25151000	679	2	1.1354E-06	29	4.27098675	NA	NA
DMRcontig15040:25178101	contig15040	25178101	25178395	295	2	2.2047E-07	4	1.3559322	NA	NA
DMRcontig15065:25214609	contig15065	25214609	25214800	192	2	1.1167E-11	2	1.04166667	NA	NA
DMRcontig15083:25241901	contig15083	25241901	25242300	400	2	1.7023E-08	7	1.75	NA	NA
DMRcontig15250:25486401	contig15250	25486401	25486700	300	2	2.471E-06	14	4.66666667	NA	NA
DMRcontig15274:25521701	contig15274	25521701	25522300	600	2	1.3929E-08	4	0.66666667	NA	NA
DMRcontig15370:25663101	contig15370	25663101	25663366	266	1	8.4504E-06	5	1.87969925	NA	NA
DMRcontig15378:25675501	contig15378	25675501	25675800	300	1	2.8909E-06	6	2	NA	NA
DMRcontig15400:25707261	contig15400	25707261	25707500	240	1	4.0721E-07	7	2.91666667	NA	NA
DMRcontig15406:25716201	contig15406	25716201	25716500	300	1	4.3933E-07	4	1.33333333	NA	NA
DMRcontig15419:25735590	contig15419	25735590	25735700	111	1	7.9833E-06	4	3.6036036	NA	NA

DMRcontig15465:25801701	contig15465	25801701	25802000	300	2	1.6633E-10	6	2	NA	NA
DMRcontig15481:25825701	contig15481	25825701	25826274	574	1	2.1727E-06	27	4.70383275	NA	NA
DMRcontig15501:25856201	contig15501	25856201	25856384	184	1	2.8478E-06	7	3.80434783	NA	NA
DMRcontig15538:25909201	contig15538	25909201	25909391	191	1	3.9966E-06	5	2.61780105	NA	NA
DMRcontig15561:25942423	contig15561	25942423	25942899	477	5	2.6705E-20	4	0.83857442	NA	NA
DMRcontig15567:25951601	contig15567	25951601	25951800	200	1	9.01E-06	1	0.5	NA	NA
DMRcontig15587:25979921	contig15587	25979921	25980351	431	1	7.0608E-09	14	3.24825986	NA	NA
DMRcontig15604:26004793	contig15604	26004793	26005200	408	2	1.052E-07	9	2.20588235	NA	NA
DMRcontig15617:26023401	contig15617	26023401	26023805	405	1	5.2638E-08	27	6.66666667	NA	NA
DMRcontig15627:26038318	contig15627	26038318	26038700	383	3	3.7476E-07	11	2.87206266	NA	NA
DMRcontig15631:26044129	contig15631	26044129	26044577	449	1	1.5742E-06	7	1.55902004	NA	NA
DMRcontig15635:26049624	contig15635	26049624	26050062	439	2	2.0868E-07	18	4.10022779	NA	NA
DMRcontig15640:26057401	contig15640	26057401	26057789	389	1	6.5152E-06	14	3.59897172	NA	NA
DMRcontig15692:26131076	contig15692	26131076	26131300	225	1	1.4996E-06	8	3.55555556	NA	NA
DMRcontig15696:26136401	contig15696	26136401	26136900	500	2	2.8931E-06	10	2	NA	NA
DMRcontig15734:26192201	contig15734	26192201	26192795	595	2	2.0162E-06	37	6.21848739	NA	NA
DMRcontig15783:26263503	contig15783	26263503	26263762	260	2	2.0438E-06	6	2.30769231	NA	NA
DMRcontig15793:26277442	contig15793	26277442	26277900	459	2	9.5413E-06	21	4.5751634	NA	NA
DMRcontig15840:26346935	contig15840	26346935	26347500	566	1	3.1314E-08	16	2.82685512	NA	NA
DMRcontig15871:26391751	contig15871	26391751	26392000	250	1	1.3899E-07	3	1.2	NA	NA
DMRcontig15875:26397585	contig15875	26397585	26397800	216	1	5.4174E-06	13	6.01851852	NA	NA
DMRcontig15922:26464801	contig15922	26464801	26465252	452	1	1.6767E-07	22	4.86725664	NA	NA
DMRcontig16002:26580101	contig16002	26580101	26580500	400	1	2.8096E-06	11	2.75	NA	NA
DMRcontig16026:26615366	contig16026	26615366	26615700	335	2	3.7894E-06	6	1.79104478	NA	NA
DMRcontig16038:26632901	contig16038	26632901	26633398	498	1	2.3882E-07	15	3.01204819	NA	NA
DMRcontig16049:26649201	contig16049	26649201	26649700	500	1	8.7139E-06	16	3.2	NA	NA
DMRcontig16067:26675770	contig16067	26675770	26676000	231	1	1.8463E-06	3	1.2987013	NA	NA
DMRcontig16101:26725001	contig16101	26725001	26725400	400	1	5.6658E-06	21	5.25	NA	NA
DMRcontig16114:26743481	contig16114	26743481	26744000	520	2	8.794E-09	16	3.07692308	NA	NA
DMRcontig16116:26746679	contig16116	26746679	26747047	369	1	8.3562E-06	2	0.54200542	NA	NA
DMRcontig16142:26783624	contig16142	26783624	26784188	565	1	3.7423E-07	39	6.90265487	NA	NA
DMRcontig16218:26896001	contig16218	26896001	26896300	300	1	5.1501E-08	15	5	NA	NA
DMRcontig16229:26911626	contig16229	26911626	26912000	375	1	2.9926E-08	10	2.66666667	NA	NA
DMRcontig16234:26918901	contig16234	26918901	26919300	400	1	1.6871E-07	3	0.75	NA	NA
DMRcontig16301:27017434	contig16301	27017434	27017600	167	1	6.9535E-06	6	3.59281437	NA	NA
DMRcontig16488:27287413	contig16488	27287413	27287678	266	2	1.1963E-07	10	3.7593985	NA	NA
DMRcontig16622:27480201	contig16622	27480201	27480400	200	1	9.4081E-06	5	2.5	NA	NA
DMRcontig16623:27481701	contig16623	27481701	27481971	271	1	1.6207E-06	4	1.47601476	NA	NA
DMRcontig16630:27491501	contig16630	27491501	27491900	400	1	1.5004E-07	12	3	NA	NA
DMRcontig16678:27561601	contig16678	27561601	27562000	400	1	2.8498E-06	7	1.75	NA	NA
DMRcontig16719:27621626	contig16719	27621626	27622000	375	1	1.0989E-06	10	2.66666667	NA	NA
DMRcontig16742:27655401	contig16742	27655401	27655800	400	1	4.5132E-07	9	2.25	NA	NA
DMRcontig16869:27839901	contig16869	27839901	27840400	500	1	1.092E-06	23	4.6	NA	NA
DMRcontig16902:27887401	contig16902	27887401	27887700	300	1	1.6305E-06	3	1	NA	NA
DMRcontig16907:27894501	contig16907	27894501	27895100	600	2	8.462E-09	12	2	NA	NA
DMRcontig17096:28166199	contig17096	28166199	28166500	302	1	1.1737E-07	10	3.31125828	NA	NA
DMRcontig17170:28270801	contig17170	28270801	28271755	955	1	7.3376E-07	12	1.2565445	NA	NA
DMRcontig17180:28285033	contig17180	28285033	28285400	368	1	3.3766E-08	22	5.97826087	NA	NA
DMRcontig17306:28464336	contig17306	28464336	28464500	165	1	9.0194E-07	1	0.60606061	NA	NA
DMRcontig17352:28529314	contig17352	28529314	28529700	387	2	4.7937E-08	2	0.51679587	NA	NA
DMRcontig17513:28756101	contig17513	28756101	28756500	400	1	6.692E-06	18	4.5	NA	NA
DMRcontig17515:28758832	contig17515	28758832	28759200	369	1	5.3195E-06	9	2.43902439	NA	NA
DMRcontig17530:28780601	contig17530	28780601	28781152	552	1	6.2823E-06	16	2.89855072	NA	NA
DMRcontig17532:28783347	contig17532	28783347	28783767	421	2	4.2388E-06	14	3.32541568	NA	NA
DMRcontig17543:28799001	contig17543	28799001	28799259	259	1	6.3664E-06	6	2.31660232	NA	NA
DMRcontig17545:28801743	contig17545	28801743	28801900	158	1	8.8705E-06	4	2.53164557	NA	NA
DMRcontig17573:28841201	contig17573	28841201	28841500	300	1	2.2901E-06	4	1.33333333	NA	NA
DMRcontig17604:28886260	contig17604	28886260	28886632	373	2	1.3561E-09	8	2.14477212	NA	NA
DMRcontig17650:28953201	contig17650	28953201	28953673	473	1	6.4844E-08	21	4.4397463	NA	NA
DMRcontig17690:29009615	contig17690	29009615	29009827	213	1	2.5542E-08	1	0.46948357	NA	NA
DMRcontig17739:29077444	contig17739	29077444	29077900	457	1	7.9438E-07	2	0.43763676	NA	NA
DMRcontig17753:29099201	contig17753	29099201	29099400	200	1	2.0731E-06	3	1.5	NA	NA
DMRcontig17893:29296901	contig17893	29296901	29297100	200	2	1.8432E-10	8	4	NA	NA
DMRcontig17974:29410301	contig17974	29410301	29410600	300	1	2.4611E-06	3	1	NA	NA
DMRcontig17988:29429801	contig17988	29429801	29430100	300	1	4.4853E-07	13	4.33333333	NA	NA
DMRcontig18047:29512546	contig18047	29512546	29512777	232	1	3.8814E-10	7	3.01724138	NA	NA
DMRcontig18079:29556101	contig18079	29556101	29556500	400	1	1.878E-06	16	4	NA	NA

DMRcontig18177:29695401	contig18177	29695401	29696034	634	3	1.347E-08	23	3.62776025	NA	NA
DMRcontig18196:29723401	contig18196	29723401	29723925	525	1	3.117E-07	7	1.33333333	NA	NA
DMRcontig18220:29755175	contig18220	29755175	29755385	211	1	7.9071E-08	6	2.8436019	NA	NA
DMRcontig18224:29760701	contig18224	29760701	29760900	200	1	2.5165E-07	2	1	NA	NA
DMRcontig18225:29762101	contig18225	29762101	29762377	277	1	1.3798E-07	11	3.97111913	NA	NA
DMRcontig18273:29832101	contig18273	29832101	29832697	597	3	1.9461E-08	9	1.50753769	NA	NA
DMRcontig18391:29998101	contig18391	29998101	29998592	492	2	5.6658E-06	6	1.2195122	NA	NA
DMRcontig18438:30067501	contig18438	30067501	30067800	300	1	4.3508E-07	35	11.6666667	NA	NA
DMRcontig18443:30074301	contig18443	30074301	30074552	252	1	1.6351E-13	13	5.15873016	NA	NA
DMRcontig18511:30175146	contig18511	30175146	30175300	155	1	3.9421E-07	2	1.29032258	NA	NA
DMRcontig18520:30187401	contig18520	30187401	30188100	700	2	1.6305E-06	9	1.28571429	NA	NA
DMRcontig18565:30252145	contig18565	30252145	30252600	456	3	2.4275E-09	13	2.85087719	NA	NA
DMRcontig18588:30286401	contig18588	30286401	30286818	418	2	1.3005E-09	12	2.8708134	NA	NA
DMRcontig18622:30333601	contig18622	30333601	30333900	300	1	9.882E-08	5	1.66666667	NA	NA
DMRcontig18671:30399901	contig18671	30399901	30400296	396	1	2.1469E-09	17	4.29292929	NA	NA
DMRcontig18713:30466501	contig18713	30466501	30467323	823	2	1.5347E-07	28	3.40218712	NA	NA
DMRcontig18719:30476008	contig18719	30476008	30476300	293	1	9.0545E-06	4	1.36518771	NA	NA
DMRcontig18737:30505414	contig18737	30505414	30506300	887	6	1.1898E-12	58	6.53889515	NA	NA
DMRcontig18751:30529301	contig18751	30529301	30529780	480	3	1.8686E-09	7	1.45833333	NA	NA
DMRcontig18781:30575701	contig18781	30575701	30576000	300	1	8.8293E-06	2	0.66666667	NA	NA
DMRcontig18782:30577401	contig18782	30577401	30577900	500	1	2.6062E-06	20	4	NA	NA
DMRcontig18824:30644361	contig18824	30644361	30644900	540	2	1.0584E-07	22	4.07407407	NA	NA
DMRcontig18836:30663101	contig18836	30663101	30663600	500	1	1.1159E-08	30	6	NA	NA
DMRcontig18848:30682501	contig18848	30682501	30683500	1000	5	3.0501E-12	13	1.3	NA	NA
DMRcontig18877:30728201	contig18877	30728201	30728400	200	1	2.6855E-07	3	1.5	NA	NA
DMRcontig18892:30751701	contig18892	30751701	30752595	895	3	5.9577E-18	36	4.02234637	NA	NA
DMRcontig18897:30759444	contig18897	30759444	30759641	198	1	1.5059E-07	0	0	NA	NA
DMRcontig19031:30964005	contig19031	30964005	30964200	196	1	1.5693E-06	7	3.57142857	NA	NA
DMRcontig19048:30989914	contig19048	30989914	30990300	387	3	4.7816E-13	15	3.87596899	NA	NA
DMRcontig19061:31009208	contig19061	31009208	31009800	593	1	8.6678E-06	9	1.51770658	NA	NA
DMRcontig19089:31050838	contig19089	31050838	31051100	263	2	1.1023E-09	2	0.76045627	NA	NA
DMRcontig19240:31275301	contig19240	31275301	31275449	149	1	7.3021E-09	5	3.3557047	NA	NA
DMRcontig19262:31308253	contig19262	31308253	31308500	248	1	6.5965E-06	5	2.01612903	NA	NA
DMRcontig19294:31358801	contig19294	31358801	31359600	800	1	1.4075E-06	25	3.125	EMWEY_00012150;cno	Unknown
DMRcontig19369:31470101	contig19369	31470101	31470600	500	2	9.459E-09	22	4.4	NA	NA
DMRcontig19370:31471801	contig19370	31471801	31472400	600	2	5.6251E-07	18	3	NA	NA
DMRcontig19376:31480501	contig19376	31480501	31480700	200	1	3.7754E-07	7	3.5	NA	NA
DMRcontig19388:31498901	contig19388	31498901	31499499	599	3	5.2406E-10	39	6.51085142	NA	NA
DMRcontig19446:31583001	contig19446	31583001	31583200	200	1	8.4066E-06	15	7.5	LOC105910096	Metabolism
DMRcontig19453:31593301	contig19453	31593301	31594295	995	1	1.1741E-06	67	6.73366834	NA	NA
DMRcontig19520:31692898	contig19520	31692898	31693100	203	1	3.931E-08	6	2.95566502	NA	NA
DMRcontig19543:31725001	contig19543	31725001	31725200	200	1	3.823E-06	0	0	NA	NA
DMRcontig19590:31792683	contig19590	31792683	31792973	291	3	4.5721E-11	19	6.52920962	NA	NA
DMRcontig19631:31851601	contig19631	31851601	31851800	200	2	7.4429E-13	5	2.5	NA	NA
DMRcontig19659:31892501	contig19659	31892501	31892770	270	2	5.8056E-07	7	2.59259259	NA	NA
DMRcontig19751:32025501	contig19751	32025501	32025700	200	1	1.5877E-08	7	3.5	NA	NA
DMRcontig19845:32163053	contig19845	32163053	32163300	248	1	1.0431E-06	16	6.4516129	NA	NA
DMRcontig19934:32294101	contig19934	32294101	32294300	200	1	3.6358E-09	1	0.5	NA	NA
DMRcontig19935:32295565	contig19935	32295565	32295852	288	2	8.0123E-09	8	2.77777778	NA	NA
DMRcontig20061:32485894	contig20061	32485894	32486100	207	1	2.2325E-06	0	0	NA	NA
DMRcontig20229:32752601	contig20229	32752601	32753000	400	2	1.6733E-06	5	1.25	NA	NA
DMRcontig20248:32781652	contig20248	32781652	32782200	549	5	1.1786E-10	36	6.55737705	NA	NA
DMRcontig20254:32791601	contig20254	32791601	32791900	300	1	6.7595E-07	17	5.66666667	Trnae-cuc	Unknown
DMRcontig20255:32793047	contig20255	32793047	32793400	354	1	6.4176E-06	6	1.69491525	NA	NA
DMRcontig20358:32944756	contig20358	32944756	32945200	445	4	5.982E-10	22	4.94382022	CNPV234	Cytoskeleton
DMRcontig20610:33289320	contig20610	33289320	33289500	181	2	1.7376E-59	0	0	NA	NA
DMRcontig20618:33299708	contig20618	33299708	33299900	193	1	1.7146E-12	3	1.55440415	NA	NA
DMRcontig20735:33448801	contig20735	33448801	33449230	430	3	8.0143E-08	22	5.11627907	NA	NA
DMRcontig20987:33772201	contig20987	33772201	33772376	176	1	1.6845E-09	4	2.27272727	NA	NA
DMRcontig21247:34110474	contig21247	34110474	34110858	385	2	1.8238E-08	9	2.33766234	NA	NA
DMRcontig21494:34379964	contig21494	34379964	34380200	237	1	2.618E-07	7	2.9535865	NA	NA
DMRcontig21892:34823701	contig21892	34823701	34823900	200	1	1.3395E-07	11	5.5	NA	NA
DMRcontig22177:35150101	contig22177	35150101	35150400	300	1	5.9747E-06	6	2	NA	NA
DMRcontig22775:35848961	contig22775	35848961	35849340	380	1	9.1317E-06	11	2.89473684	NA	NA
DMRcontig22832:35916028	contig22832	35916028	35916413	386	2	1.0782E-10	5	1.29533679	NA	NA
DMRcontig23099:36230501	contig23099	36230501	36230656	156	2	8.5414E-08	12	7.69230769	NA	NA
DMRcontig23331:36500325	contig23331	36500325	36500500	176	1	4.8282E-09	3	1.70454545	NA	NA

DMRcontig23460:36649722	contig23460	36649722	36650100	379	1	8.2675E-06	18	4.74934037	NA	NA
DMRcontig23683:36906892	contig23683	36906892	36907000	109	1	3.2335E-09	0	0	NA	NA
DMRcontig24120:37456550	contig24120	37456550	37457135	586	1	1.9848E-07	15	2.55972696	NA	NA
DMRcontig24132:37475401	contig24132	37475401	37475700	300	2	1.2593E-06	7	2.33333333	NA	NA
DMRcontig24157:37513101	contig24157	37513101	37513600	500	3	4.7863E-13	22	4.4	NA	NA
DMRcontig24177:37542917	contig24177	37542917	37543200	284	1	8.4054E-06	12	4.22535211	NA	NA
DMRcontig24178:37544301	contig24178	37544301	37544618	318	2	1.5934E-10	11	3.4591195	NA	NA
DMRcontig24211:37592901	contig24211	37592901	37593200	300	2	1.3514E-09	27	9	NA	NA
DMRcontig24216:37600056	contig24216	37600056	37600400	345	2	3.2056E-13	15	4.34782609	NA	NA
DMRcontig24247:37646101	contig24247	37646101	37646400	300	1	2.5521E-06	6	2	NA	NA
DMRcontig24323:37755301	contig24323	37755301	37755500	200	2	2.6971E-07	13	6.5	NA	NA
DMRcontig24370:37820915	contig24370	37820915	37821100	186	1	8.2145E-06	7	3.76344086	NA	NA
DMRcontig24473:37961205	contig24473	37961205	37961640	436	2	6.4507E-10	11	2.52293578	NA	NA
DMRcontig24552:38064011	contig24552	38064011	38064152	142	1	1.7256E-06	1	0.70422535	NA	NA
DMRcontig24604:38127222	contig24604	38127222	38127700	479	1	1.6761E-06	18	3.75782881	NA	NA
DMRcontig24664:38208464	contig24664	38208464	38208635	172	1	9.1739E-06	1	0.58139535	NA	NA
DMRcontig24789:38366427	contig24789	38366427	38366796	370	2	2.6714E-07	15	4.05405405	NA	NA
DMRcontig24989:38620485	contig24989	38620485	38620984	500	2	2.8479E-09	30	6	NA	NA
DMRcontig24993:38626701	contig24993	38626701	38626900	200	1	4.3784E-06	13	6.5	LOC102794729;rpl23a	Unknown;Translation
DMRcontig25231:38892416	contig25231	38892416	38892493	78	1	7.4352E-06	5	6.41025641	NA	NA
DMRcontig25919:39675101	contig25919	39675101	39675372	272	1	6.5574E-08	26	9.55882353	NA	NA
DMRcontig26015:39788057	contig26015	39788057	39788270	214	1	1.6985E-06	8	3.73831776	NA	NA
DMRcontig26188:39996101	contig26188	39996101	39996382	282	1	5.4174E-06	9	3.19148936	NA	NA
DMRcontig26323:40155350	contig26323	40155350	40155730	381	2	1.4808E-07	10	2.62467192	NA	NA
DMRcontig26379:40222001	contig26379	40222001	40222238	238	2	6.4757E-15	2	0.84033613	NA	NA
DMRcontig26480:40341101	contig26480	40341101	40341300	200	2	5.9542E-08	8	4	NA	NA
DMRcontig26614:40496401	contig26614	40496401	40496587	187	2	3.6947E-08	22	11.7647059	NA	NA
DMRcontig26908:40842134	contig26908	40842134	40842400	267	2	2.2281E-06	13	4.86891386	NA	NA
DMRcontig27224:41211701	contig27224	41211701	41211940	240	1	7.81E-09	4	1.66666667	NA	NA
DMRcontig27397:41449201	contig27397	41449201	41449400	200	2	3.823E-06	2	1	NA	NA
DMRcontig27428:41498801	contig27428	41498801	41499200	400	3	1.9605E-08	21	5.25	NA	NA
DMRcontig27457:41545201	contig27457	41545201	41545500	300	2	2.8447E-06	8	2.66666667	NA	NA
DMRcontig27649:41836006	contig27649	41836006	41836400	395	1	3.4016E-07	33	8.35443038	NA	NA
DMRcontig27701:41914001	contig27701	41914001	41914355	355	1	8.4579E-08	22	6.1971831	NA	NA
DMRcontig27707:41922801	contig27707	41922801	41923092	292	1	1.5824E-10	1	0.34246575	NA	NA
DMRcontig27714:41932601	contig27714	41932601	41932874	274	1	3.9946E-06	5	1.82481752	NA	NA
DMRcontig27759:41996802	contig27759	41996802	41997200	399	2	4.9215E-10	7	1.75438596	NA	NA
DMRcontig27828:42095135	contig27828	42095135	42095700	566	2	4.7339E-09	9	1.59010601	NA	NA
DMRcontig27870:42153801	contig27870	42153801	42154000	200	1	1.417E-06	22	11	NA	NA
DMRcontig27883:42172378	contig27883	42172378	42172800	423	4	4.1831E-11	21	4.96453901	NA	NA
DMRcontig27900:42196811	contig27900	42196811	42197186	376	1	3.0734E-09	13	3.45744681	NA	NA
DMRcontig27901:42198301	contig27901	42198301	42198700	400	2	8.1196E-07	11	2.75	NA	NA
DMRcontig27964:42284101	contig27964	42284101	42284500	400	1	9.6656E-06	24	6	NA	NA
DMRcontig28063:42414144	contig28063	42414144	42414400	257	1	4.1636E-08	3	1.16731518	NA	NA
DMRcontig28086:42442715	contig28086	42442715	42442775	61	1	1.9306E-23	1	1.63934426	NA	NA
DMRcontig28192:42582858	contig28192	42582858	42583000	143	1	4.9759E-06	2	1.3986014	NA	NA
DMRcontig28204:42599701	contig28204	42599701	42600089	389	2	4.9744E-07	5	1.28534704	NA	NA
DMRcontig28261:42676801	contig28261	42676801	42677300	500	3	8.5838E-09	7	1.4	NA	NA
DMRcontig28285:42710101	contig28285	42710101	42710300	200	1	2.7581E-09	1	0.5	NA	NA
DMRcontig28429:42896201	contig28429	42896201	42896300	100	1	6.5906E-06	4	4	NA	NA
DMRcontig28476:42951401	contig28476	42951401	42951700	300	2	1.172E-06	14	4.66666667	NA	NA
DMRcontig28568:43077201	contig28568	43077201	43077800	600	1	5.4589E-06	21	3.5	NA	NA
DMRcontig28659:43216601	contig28659	43216601	43217000	400	2	2.0928E-07	6	1.5	NA	NA
DMRcontig28672:43236001	contig28672	43236001	43236400	400	2	7.6403E-10	6	1.5	NA	NA
DMRcontig29161:43784552	contig29161	43784552	43785000	449	2	1.8464E-07	24	5.34521158	NA	NA
DMRcontig29220:43853501	contig29220	43853501	43853577	77	1	3.0477E-07	1	1.2987013	NA	NA
DMRcontig29286:43928527	contig29286	43928527	43928727	201	1	7.8434E-06	12	5.97014925	NA	NA
DMRcontig29322:43968981	contig29322	43968981	43969300	320	1	9.1689E-06	8	2.5	NA	NA
DMRcontig29468:44135629	contig29468	44135629	44135900	272	1	6.1906E-06	8	2.94117647	NA	NA
DMRcontig29765:44486801	contig29765	44486801	44487100	300	1	9.8695E-06	6	2	NA	NA
DMRcontig29779:44504338	contig29779	44504338	44504545	208	1	1.4303E-06	6	2.88461538	NA	NA
DMRcontig29820:44553411	contig29820	44553411	44553859	449	4	3.0985E-09	24	5.34521158	NA	NA
DMRcontig29990:44753201	contig29990	44753201	44753500	300	2	1.7279E-06	8	2.66666667	NA	NA
DMRcontig30070:44847477	contig30070	44847477	44847631	155	1	1.3829E-08	3	1.93548387	NA	NA
DMRcontig30129:44919201	contig30129	44919201	44919600	400	1	5.3828E-08	23	5.75	NA	NA
DMRcontig30440:45292001	contig30440	45292001	45292300	300	2	1.3974E-09	3	1	NA	NA
DMRcontig30540:45412001	contig30540	45412001	45412105	105	1	7.5957E-07	0	0	NA	NA

DMRcontig30605:45489522	contig30605	45489522	45489700	179	1	9.3955E-07	11	6.1452514	NA	NA
DMRcontig30748:45661185	contig30748	45661185	45661277	93	1	3.5789E-09	3	3.22580645	NA	NA
DMRcontig30771:45689401	contig30771	45689401	45689500	100	1	3.823E-06	4	4	NA	NA
DMRcontig30779:45698908	contig30779	45698908	45699240	333	2	9.0223E-10	10	3.003003	NA	NA
DMRcontig30826:45755201	contig30826	45755201	45755585	385	2	4.9145E-07	6	1.55844156	NA	NA
DMRcontig30866:45803039	contig30866	45803039	45803257	219	2	4.6412E-14	6	2.73972603	NA	NA
DMRcontig30932:45879969	contig30932	45879969	45880036	68	2	3.3732E-07	3	4.41176471	NA	NA
DMRcontig30938:45886901	contig30938	45886901	45887100	200	1	4.6671E-06	3	1.5	NA	NA
DMRcontig31048:46015206	contig31048	46015206	46015500	295	1	1.195E-09	36	12.2033898	NA	NA
DMRcontig31168:46172301	contig31168	46172301	46172800	500	4	1.0396E-12	23	4.6	NA	NA
DMRcontig31318:46402101	contig31318	46402101	46402317	217	1	5.0238E-06	7	3.22580645	NA	NA
DMRcontig31465:46608801	contig31465	46608801	46609100	300	1	1.8893E-06	4	1.33333333	NA	NA
DMRcontig31471:46617135	contig31471	46617135	46617671	537	1	1.4377E-06	7	1.30353818	NA	NA
DMRcontig31525:46691727	contig31525	46691727	46691900	174	2	2.8148E-08	2	1.14942529	NA	NA
DMRcontig31577:46761801	contig31577	46761801	46762198	398	1	3.3787E-06	14	3.51758794	NA	NA
DMRcontig31594:46784533	contig31594	46784533	46784700	168	2	9.6058E-11	9	5.35714286	NA	NA
DMRcontig31612:46807101	contig31612	46807101	46807500	400	1	7.1486E-06	14	3.5	NA	NA
DMRcontig31721:46945434	contig31721	46945434	46945696	263	2	1.3119E-09	2	0.76045627	NA	NA
DMRcontig31894:47164801	contig31894	47164801	47165000	200	1	1.18E-06	18	9	NA	NA
DMRcontig32074:47385113	contig32074	47385113	47385199	87	1	1.9602E-08	0	0	NA	NA
DMRcontig32100:47417651	contig32100	47417651	47417869	219	1	4.2252E-07	2	0.91324201	NA	NA
DMRcontig32635:48023201	contig32635	48023201	48023593	393	1	1.8541E-06	17	4.32569975	NA	NA
DMRcontig32670:48063601	contig32670	48063601	48063829	229	1	3.3953E-10	11	4.80349345	NA	NA
DMRcontig32826:48240901	contig32826	48240901	48241300	400	1	2.1322E-06	7	1.75	NA	NA
DMRcontig33026:48467215	contig33026	48467215	48467400	186	2	5.9909E-09	9	4.83870968	NA	NA
DMRcontig33157:48617801	contig33157	48617801	48618000	200	1	1.3153E-07	5	2.5	NA	NA
DMRcontig33273:48750274	contig33273	48750274	48750600	327	3	1.6527E-10	8	2.44648318	NA	NA
DMRcontig33515:49030386	contig33515	49030386	49030881	496	2	6.7602E-09	28	5.64516129	NA	NA
DMRcontig34014:49605401	contig34014	49605401	49605667	267	1	3.8651E-07	5	1.87265918	NA	NA
DMRcontig34132:49744201	contig34132	49744201	49744425	225	1	4.7824E-07	10	4.44444444	NA	NA
DMRcontig34178:49797801	contig34178	49797801	49798120	320	2	2.6278E-07	13	4.0625	NA	NA
DMRcontig34205:49830101	contig34205	49830101	49830380	280	1	8.3836E-06	7	2.5	NA	NA
DMRcontig34217:49844601	contig34217	49844601	49844871	271	2	4.6555E-07	16	5.90405904	NA	NA
DMRcontig34798:50514121	contig34798	50514121	50514349	229	1	1.9644E-06	9	3.930131	NA	NA
DMRcontig35266:51179601	contig35266	51179601	51179800	200	1	3.705E-06	5	2.5	NA	NA
DMRcontig35306:51240101	contig35306	51240101	51240400	300	1	4.7657E-06	21	7	NA	NA
DMRcontig35321:51263501	contig35321	51263501	51264000	500	2	7.5566E-14	20	4	NA	NA
DMRcontig35334:51282701	contig35334	51282701	51283100	400	1	7.0824E-09	19	4.75	NA	NA
DMRcontig35398:51384401	contig35398	51384401	51384766	366	1	1.6537E-09	6	1.63934426	NA	NA
DMRcontig35410:51403039	contig35410	51403039	51403300	262	1	1.2523E-06	3	1.14503817	NA	NA
DMRcontig35446:51458648	contig35446	51458648	51459000	353	1	9.2221E-06	11	3.11614731	NA	NA
DMRcontig35514:51561733	contig35514	51561733	51562000	268	1	4.4052E-09	4	1.49253731	NA	NA
DMRcontig35638:51752101	contig35638	51752101	51752500	400	1	6.5789E-07	10	2.5	NA	NA
DMRcontig35719:51877321	contig35719	51877321	51877600	280	1	4.9463E-08	1	0.35714286	NA	NA
DMRcontig35725:51886132	contig35725	51886132	51886300	169	1	1.0637E-08	8	4.73372781	NA	NA
DMRcontig35732:51896501	contig35732	51896501	51896831	331	1	1.8757E-06	6	1.81268882	NA	NA
DMRcontig35746:51917901	contig35746	51917901	51918492	592	2	3.7511E-07	16	2.7027027	NA	NA
DMRcontig35795:51991851	contig35795	51991851	51992418	568	3	7.8782E-16	25	4.40140845	NA	NA
DMRcontig35857:52084658	contig35857	52084658	52085100	443	3	3.7413E-11	28	6.32054176	NA	NA
DMRcontig35887:52128880	contig35887	52128880	52129398	519	3	2.1039E-08	19	3.66088632	NA	NA
DMRcontig35995:52290285	contig35995	52290285	52290500	216	2	7.2123E-07	5	2.31481481	NA	NA
DMRcontig36074:52409901	contig36074	52409901	52410200	300	2	2.693E-10	16	5.33333333	NA	NA
DMRcontig36117:52473201	contig36117	52473201	52473600	400	1	7.375E-08	12	3	NA	NA
DMRcontig36144:52513574	contig36144	52513574	52513900	327	3	1.891E-14	13	3.97553517	NA	NA
DMRcontig36183:52572397	contig36183	52572397	52572900	504	1	2.4566E-07	21	4.16666667	NA	NA
DMRcontig36194:52588234	contig36194	52588234	52588700	467	1	1.3761E-06	31	6.63811563	NA	NA
DMRcontig36216:52620757	contig36216	52620757	52621000	244	1	7.0716E-07	7	2.86885246	NA	NA
DMRcontig36246:52665025	contig36246	52665025	52665588	564	5	1.0744E-15	30	5.31914894	NA	NA
DMRcontig36371:52849001	contig36371	52849001	52849448	448	3	3.3891E-13	32	7.14285714	NA	NA
DMRcontig36381:52864281	contig36381	52864281	52864780	500	1	4.7169E-06	10	2	NA	NA
DMRcontig36382:52866101	contig36382	52866101	52866398	298	1	9.0142E-07	4	1.34228188	NA	NA
DMRcontig36557:53125201	contig36557	53125201	53125545	345	1	9.9233E-07	22	6.37681159	NA	NA
DMRcontig36581:53161172	contig36581	53161172	53161400	229	2	1.4963E-11	4	1.74672489	NA	NA
DMRcontig36865:53585701	contig36865	53585701	53586000	300	1	6.6129E-07	4	1.33333333	NA	NA
DMRcontig36904:53641901	contig36904	53641901	53642300	400	1	7.0589E-07	9	2.25	NA	NA
DMRcontig36967:53734201	contig36967	53734201	53734558	358	2	1.7897E-13	15	4.18994413	NA	NA
DMRcontig36996:53777301	contig36996	53777301	53777500	200	1	2.0032E-06	4	2	NA	NA

DMRcontig37023:53817301	contig37023	53817301	53817781	481	2	2.5797E-08	19	3.95010395	NA	NA
DMRcontig37028:53824528	contig37028	53824528	53825041	514	3	4.2349E-09	16	3.11284047	NA	NA
DMRcontig37054:53862153	contig37054	53862153	53862600	448	3	8.3003E-11	20	4.46428571	NA	NA
DMRcontig37105:53936701	contig37105	53936701	53936900	200	1	8.7667E-06	4	2	NA	NA
DMRcontig37189:54062201	contig37189	54062201	54062400	200	1	1.9012E-06	16	8	NA	NA
DMRcontig37197:54074122	contig37197	54074122	54074600	479	1	6.0722E-07	29	6.05427975	NA	NA
DMRcontig37230:54123337	contig37230	54123337	54123800	464	1	8.4054E-06	35	7.54310345	NA	NA
DMRcontig37266:54176701	contig37266	54176701	54177100	400	2	1.1947E-13	9	2.25	NA	NA
DMRcontig37275:54189766	contig37275	54189766	54190100	335	3	4.0173E-13	24	7.1641791	NA	NA
DMRcontig37293:54217210	contig37293	54217210	54217400	191	1	1.1287E-08	1	0.52356021	NA	NA
DMRcontig37303:54231447	contig37303	54231447	54232200	754	2	9.1026E-08	36	4.77453581	NA	NA
DMRcontig37306:54236201	contig37306	54236201	54236700	500	1	2.6436E-06	15	3	NA	NA
DMRcontig37315:54249814	contig37315	54249814	54250200	387	2	8.233E-09	18	4.65116279	NA	NA
DMRcontig37325:54264201	contig37325	54264201	54264579	379	1	5.793E-09	10	2.63852243	NA	NA
DMRcontig37344:54292039	contig37344	54292039	54292200	162	1	2.1451E-06	9	5.55555556	NA	NA
DMRcontig37353:54305001	contig37353	54305001	54305200	200	1	3.5357E-07	10	5	NA	NA
DMRcontig37385:54351001	contig37385	54351001	54351400	400	1	2.3148E-08	6	1.5	NA	NA
DMRcontig37467:54469701	contig37467	54469701	54470200	500	1	2.2847E-07	10	2	NA	NA
DMRcontig37481:54490744	contig37481	54490744	54491193	450	2	8.7352E-10	14	3.11111111	NA	NA
DMRcontig37530:54564231	contig37530	54564231	54564400	170	1	3.7665E-06	18	10.5882353	NA	NA
DMRcontig37552:54596501	contig37552	54596501	54596700	200	1	3.412E-06	5	2.5	NA	NA
DMRcontig37596:54661301	contig37596	54661301	54661835	535	1	8.4036E-07	30	5.60747664	LOC103188862	Metabolism
DMRcontig37626:54704401	contig37626	54704401	54705200	800	2	7.6687E-12	23	2.875	NA	NA
DMRcontig37665:54761101	contig37665	54761101	54761597	497	1	1.4573E-06	29	5.83501006	NA	NA
DMRcontig37683:54787806	contig37683	54787806	54788200	395	1	9.951E-06	3	0.75949367	NA	NA
DMRcontig37702:54815201	contig37702	54815201	54815600	400	2	1.7954E-09	19	4.75	NA	NA
DMRcontig37710:54827201	contig37710	54827201	54827483	283	1	4.1883E-07	11	3.8869258	NA	NA
DMRcontig37762:54903601	contig37762	54903601	54903900	300	1	2.123E-06	5	1.66666667	NA	NA
DMRcontig37763:54905001	contig37763	54905001	54905400	400	2	8.1007E-14	25	6.25	NA	NA
DMRcontig37846:55029105	contig37846	55029105	55029300	196	1	8.6792E-06	9	4.59183673	NA	NA
DMRcontig37906:55117095	contig37906	55117095	55117500	406	3	2.4093E-08	13	3.20197044	NA	NA
DMRcontig38028:55296701	contig38028	55296701	55296900	200	1	7.314E-06	3	1.5	NA	NA
DMRcontig38043:55318701	contig38043	55318701	55318900	200	1	8.1728E-09	5	2.5	NA	NA
DMRcontig38081:55374739	contig38081	55374739	55375236	498	1	3.4571E-07	9	1.80722892	NA	NA
DMRcontig38208:55560428	contig38208	55560428	55560900	473	1	1.2556E-06	25	5.28541226	NA	NA
DMRcontig38222:55581571	contig38222	55581571	55581900	330	2	1.2166E-10	8	2.42424242	NA	NA
DMRcontig38267:55647901	contig38267	55647901	55648070	170	1	9.7669E-06	0	0	NA	NA
DMRcontig38282:55669473	contig38282	55669473	55669800	328	1	6.4291E-06	9	2.74390244	NA	NA
DMRcontig38283:55670894	contig38283	55670894	55671300	407	1	7.758E-06	10	2.45700246	NA	NA
DMRcontig38325:55731841	contig38325	55731841	55732000	160	1	7.314E-06	7	4.375	NA	NA
DMRcontig38513:56005201	contig38513	56005201	56005700	500	1	5.7374E-06	20	4	NA	NA
DMRcontig38563:56077912	contig38563	56077912	56078200	289	1	7.2279E-06	7	2.42214533	NA	NA
DMRcontig38564:56079271	contig38564	56079271	56079600	330	3	3.4266E-07	14	4.24242424	NA	NA
DMRcontig38638:56185201	contig38638	56185201	56185681	481	1	7.22E-07	9	1.87110187	NA	NA
DMRcontig38674:56237101	contig38674	56237101	56237583	483	4	5.5717E-10	31	6.41821946	NA	NA
DMRcontig38705:56282328	contig38705	56282328	56282600	273	2	7.3412E-12	12	4.3956044	NA	NA
DMRcontig38751:56349024	contig38751	56349024	56349540	517	2	9.9925E-10	25	4.83558994	NA	NA
DMRcontig38818:56446201	contig38818	56446201	56446649	449	1	4.1032E-06	8	1.78173719	NA	NA
DMRcontig38856:56501501	contig38856	56501501	56501700	200	2	2.3991E-11	7	3.5	NA	NA
DMRcontig38896:56559501	contig38896	56559501	56560000	500	1	1.4847E-06	11	2.2	NA	NA
DMRcontig39026:56748201	contig39026	56748201	56748400	200	2	1.0892E-08	9	4.5	NA	NA
DMRcontig39079:56824545	contig39079	56824545	56824900	356	2	1.6876E-07	15	4.21348315	NA	NA
DMRcontig39100:56855301	contig39100	56855301	56855894	594	2	3.0603E-06	14	2.35690236	NA	NA
DMRcontig39121:56885615	contig39121	56885615	56886000	386	1	2.8113E-07	21	5.44041451	NA	NA
DMRcontig39186:56979116	contig39186	56979116	56979300	185	1	2.4883E-06	4	2.16216216	NA	NA
DMRcontig39193:56988707	contig39193	56988707	56988800	94	1	4.4959E-06	5	5.31914894	NA	NA
DMRcontig39295:57134292	contig39295	57134292	57134734	443	2	1.1035E-06	8	1.80586907	NA	NA
DMRcontig39319:57168601	contig39319	57168601	57168800	200	1	5.3134E-07	10	5	NA	NA
DMRcontig39402:57287601	contig39402	57287601	57287887	287	2	2.3403E-13	19	6.62020906	NA	NA
DMRcontig39594:57565200	contig39594	57565200	57565676	477	1	1.0562E-08	8	1.67714885	NA	NA
DMRcontig39599:57572601	contig39599	57572601	57572800	200	1	1.2211E-07	2	1	NA	NA
DMRcontig39608:57585501	contig39608	57585501	57585763	263	1	4.3168E-07	9	3.42205323	NA	NA
DMRcontig39715:57739001	contig39715	57739001	57739100	100	1	7.3477E-07	5	5	NA	NA
DMRcontig39819:57886926	contig39819	57886926	57887100	175	1	1.0989E-06	9	5.14285714	NA	NA
DMRcontig39871:57961039	contig39871	57961039	57961300	262	1	1.1441E-09	8	3.05343511	NA	NA
DMRcontig39984:58124101	contig39984	58124101	58124300	200	1	2.1035E-06	10	5	NA	NA
DMRcontig40007:58157301	contig40007	58157301	58157500	200	1	9.3668E-06	11	5.5	NA	NA

DMRcontig40157:58371642	contig40157	58371642	58372052	411	1	1.1729E-07	12	2.91970803	NA	NA
DMRcontig40195:58425201	contig40195	58425201	58426000	800	1	2.8036E-07	29	3.625	NA	NA
DMRcontig40207:58442734	contig40207	58442734	58443100	367	2	1.1386E-08	5	1.36239782	NA	NA
DMRcontig40217:58456801	contig40217	58456801	58457324	524	1	3.1478E-06	36	6.87022901	NA	NA
DMRcontig40252:58506401	contig40252	58506401	58506519	119	1	9.0847E-08	1	0.84033613	si:dkeyp-61b2.1	Unknown
DMRcontig40257:58513270	contig40257	58513270	58513600	331	2	3.5343E-13	9	2.71903323	NA	NA
DMRcontig40301:58576701	contig40301	58576701	58576982	282	1	4.2458E-06	12	4.25531915	LOC101849745;ATG3	Metabolism;Unknown
DMRcontig40315:58596474	contig40315	58596474	58596999	526	1	1.7939E-06	20	3.80228137	NA	NA
DMRcontig40390:58702963	contig40390	58702963	58703684	722	2	5.9571E-08	13	1.80055402	NA	NA
DMRcontig40436:58768601	contig40436	58768601	58769084	484	4	1.0243E-17	14	2.89256198	NA	NA
DMRcontig40501:58860148	contig40501	58860148	58860500	353	1	9.1867E-09	18	5.09915014	NA	NA
DMRcontig40549:58927701	contig40549	58927701	58927900	200	1	4.8991E-06	5	2.5	NA	NA
DMRcontig40568:58954118	contig40568	58954118	58954300	183	2	2.8053E-07	8	4.3715847	NA	NA
DMRcontig40588:58982859	contig40588	58982859	58983100	242	2	2.2867E-18	13	5.37190083	NA	NA
DMRcontig40592:58988330	contig40592	58988330	58988600	271	1	2.8006E-06	20	7.3800738	NA	NA
DMRcontig40718:59164701	contig40718	59164701	59165000	300	1	5.5729E-06	23	7.66666667	NA	NA
DMRcontig40736:59190501	contig40736	59190501	59190800	300	1	3.1296E-06	18	6	NA	NA
DMRcontig40753:59215001	contig40753	59215001	59215251	251	2	7.7373E-07	8	3.187251	NA	NA
DMRcontig40758:59221801	contig40758	59221801	59222094	294	2	6.3683E-07	14	4.76190476	NA	NA
DMRcontig40821:59309601	contig40821	59309601	59309742	142	1	9.0142E-07	1	0.70422535	NA	NA
DMRcontig40827:59317816	contig40827	59317816	59318200	385	1	1.6741E-07	13	3.37662338	NA	NA
DMRcontig40863:59369001	contig40863	59369001	59369316	316	1	3.2096E-06	22	6.96202532	NA	NA
DMRcontig40868:59376201	contig40868	59376201	59376565	365	2	1.2777E-09	17	4.65753425	NA	NA
DMRcontig40946:59486401	contig40946	59486401	59486600	200	1	4.1411E-07	4	2	NA	NA
DMRcontig41002:59566101	contig41002	59566101	59566400	300	1	3.0451E-06	28	9.33333333	NA	NA
DMRcontig41055:59639601	contig41055	59639601	59639800	200	1	7.5433E-06	5	2.5	NA	NA
DMRcontig41152:59776001	contig41152	59776001	59776256	256	1	6.7905E-06	7	2.734375	NA	NA
DMRcontig41211:59859562	contig41211	59859562	59859800	239	1	2.3195E-06	6	2.51046025	NA	NA
DMRcontig41328:60023101	contig41328	60023101	60023400	300	1	6.8941E-07	8	2.66666667	NA	NA
DMRcontig41364:60074457	contig41364	60074457	60074700	244	3	3.7046E-08	12	4.91803279	NA	NA
DMRcontig41506:60273301	contig41506	60273301	60273600	300	2	1.195E-09	19	6.33333333	NA	NA
DMRcontig41614:60421936	contig41614	60421936	60422400	465	1	4.1553E-06	8	1.72043011	NA	NA
DMRcontig41731:60584201	contig41731	60584201	60584300	100	1	6.0645E-06	4	4	NA	NA
DMRcontig41778:60649227	contig41778	60649227	60649589	363	2	2.5594E-10	18	4.95867769	NA	NA
DMRcontig41820:60709201	contig41820	60709201	60709656	456	2	4.462E-07	10	2.19298246	NA	NA
DMRcontig41857:60760201	contig41857	60760201	60760600	400	1	2.6845E-07	11	2.75	NA	NA
DMRcontig41872:60780801	contig41872	60780801	60781415	615	1	2.8456E-06	31	5.04065041	NA	NA
DMRcontig41902:60822387	contig41902	60822387	60822700	314	2	3.3832E-20	22	7.00636943	NA	NA
DMRcontig41956:60897334	contig41956	60897334	60897711	378	1	8.5083E-09	23	6.08465608	NA	NA
DMRcontig41960:60903201	contig41960	60903201	60903599	399	1	5.9132E-06	6	1.5037594	NA	NA
DMRcontig41983:60935501	contig41983	60935501	60935700	200	1	3.823E-06	5	2.5	NA	NA
DMRcontig42085:61078690	contig42085	61078690	61078969	280	1	6.6356E-06	4	1.42857143	NA	NA
DMRcontig42125:61134453	contig42125	61134453	61134987	535	1	4.5088E-06	22	4.11214953	NA	NA
DMRcontig42131:61143474	contig42131	61143474	61143900	427	2	1.5327E-08	25	5.85480094	NA	NA
DMRcontig42163:61187201	contig42163	61187201	61187446	246	1	7.398E-06	4	1.62601626	NA	NA
DMRcontig42165:61190201	contig42165	61190201	61190400	200	1	4.0263E-06	3	1.5	NA	NA
DMRcontig42203:61242401	contig42203	61242401	61242661	261	1	3.0464E-06	6	2.29885057	NA	NA
DMRcontig42389:61500701	contig42389	61500701	61501300	600	1	7.9808E-06	46	7.66666667	NA	NA
DMRcontig42490:61642904	contig42490	61642904	61643146	243	2	2.8258E-08	17	6.99588477	NA	NA
DMRcontig42522:61686701	contig42522	61686701	61687185	485	4	4.2356E-16	28	5.77319588	IscW_ISCW023383;sox2	Development
DMRcontig42524:61689701	contig42524	61689701	61689900	200	2	3.6798E-06	8	4	NA	NA
DMRcontig42601:61795401	contig42601	61795401	61795600	200	1	8.3176E-08	7	3.5	NA	NA
DMRcontig42783:62046201	contig42783	62046201	62046552	352	1	3.4973E-08	15	4.26136364	NA	NA
DMRcontig42799:62068945	contig42799	62068945	62069100	156	1	1.6534E-08	1	0.64102564	NA	NA
DMRcontig42866:62159767	contig42866	62159767	62159900	134	1	2.4753E-07	2	1.49253731	NA	NA
DMRcontig42967:62300401	contig42967	62300401	62300600	200	1	8.7589E-06	5	2.5	NA	NA
DMRcontig43012:62362401	contig43012	62362401	62362500	100	1	1.4075E-06	0	0	NA	NA
DMRcontig43035:62393690	contig43035	62393690	62394000	311	1	5.6658E-06	10	3.21543408	NA	NA
DMRcontig43037:62396434	contig43037	62396434	62396800	367	2	1.3206E-10	5	1.36239782	NA	NA
DMRcontig43100:62482401	contig43100	62482401	62482755	355	1	2.9654E-08	14	3.94366197	NA	NA
DMRcontig43151:62551701	contig43151	62551701	62552171	471	2	1.252E-09	21	4.45859873	NA	NA
DMRcontig43182:62594201	contig43182	62594201	62594988	788	2	1.2666E-10	43	5.45685279	NA	NA
DMRcontig43187:62601901	contig43187	62601901	62602190	290	2	6.2109E-07	5	1.72413793	NA	NA
DMRcontig43224:62652407	contig43224	62652407	62652700	294	1	1.6912E-07	14	4.76190476	NA	NA
DMRcontig43275:62722845	contig43275	62722845	62723144	300	1	9.189E-07	6	2	NA	NA
DMRcontig43310:62770184	contig43310	62770184	62770500	317	1	1.8751E-06	5	1.57728707	NA	NA

DMRcontig43329:62795758	contig43329	62795758	62796149	392	3	8.2396E-08	9	2.29591837	NA	NA
DMRcontig43341:62811901	contig43341	62811901	62812100	200	1	2.4599E-06	5	2.5	NA	NA
DMRcontig43373:62855544	contig43373	62855544	62856100	557	2	1.4104E-07	36	6.46319569	NA	NA
DMRcontig43500:63031037	contig43500	63031037	63031300	264	1	5.3321E-07	5	1.89393939	NA	NA
DMRcontig43545:63091601	contig43545	63091601	63092095	495	1	5.2626E-06	21	4.24242424	NA	NA
DMRcontig43546:63093101	contig43546	63093101	63093300	200	1	5.8152E-06	5	2.5	NA	NA
DMRcontig43558:63109486	contig43558	63109486	63109800	315	2	4.7656E-10	6	1.9047619	NA	NA
DMRcontig43648:63231915	contig43648	63231915	63232162	248	1	7.314E-06	6	2.41935484	NA	NA
DMRcontig43685:63283121	contig43685	63283121	63283560	440	2	3.7383E-06	12	2.72727273	NA	NA
DMRcontig43699:63302901	contig43699	63302901	63303300	400	1	9.9888E-06	18	4.5	NA	NA
DMRcontig43727:63342406	contig43727	63342406	63342600	195	1	9.2463E-10	11	5.64102564	NA	NA
DMRcontig43737:63356180	contig43737	63356180	63356300	121	1	5.2713E-07	5	4.1322314	NA	NA
DMRcontig43848:63507501	contig43848	63507501	63507747	247	1	1.8054E-06	10	4.048583	NA	NA
DMRcontig43881:63552377	contig43881	63552377	63552586	210	2	1.1386E-08	6	2.85714286	NA	NA
DMRcontig43890:63564638	contig43890	63564638	63564900	263	1	1.1386E-07	6	2.28136882	NA	NA
DMRcontig43917:63600715	contig43917	63600715	63600900	186	2	4.4725E-15	11	5.91397849	NA	NA
DMRcontig43949:63643045	contig43949	63643045	63643400	356	1	5.4619E-07	6	1.68539326	NA	NA
DMRcontig43979:63684201	contig43979	63684201	63684400	200	1	9.6273E-07	5	2.5	NA	NA
DMRcontig44064:63800291	contig44064	63800291	63800500	210	1	2.0788E-06	9	4.28571429	NA	NA
DMRcontig44098:63846701	contig44098	63846701	63847023	323	2	1.014E-06	10	3.09597523	NA	NA
DMRcontig44101:63850815	contig44101	63850815	63850900	86	1	6.0978E-08	3	3.48837209	NA	NA
DMRcontig44132:63893207	contig44132	63893207	63893400	194	1	9.5512E-10	1	0.51546392	NA	NA
DMRcontig44149:63916383	contig44149	63916383	63916700	318	2	4.5879E-06	7	2.20125786	NA	NA
DMRcontig44266:64079201	contig44266	64079201	64079570	370	3	7.5377E-09	11	2.97297297	NA	NA
DMRcontig44330:64166821	contig44330	64166821	64167100	280	1	2.9994E-08	11	3.92857143	NA	NA
DMRcontig44398:64258201	contig44398	64258201	64258386	186	1	8.6678E-06	8	4.30107527	NA	NA
DMRcontig44539:64447787	contig44539	64447787	64448200	414	5	6.3008E-14	12	2.89855072	NA	NA
DMRcontig44563:64480609	contig44563	64480609	64480900	292	2	3.5361E-10	7	2.39726027	NA	NA
DMRcontig44586:64510601	contig44586	64510601	64510984	384	4	3.6241E-15	13	3.38541667	NA	NA
DMRcontig44794:64793201	contig44794	64793201	64793300	100	1	3.6514E-06	1	1	NA	NA
DMRcontig44819:64827701	contig44819	64827701	64827900	200	2	5.8649E-11	12	6	NA	NA
DMRcontig44869:64894090	contig44869	64894090	64894500	411	2	3.9234E-07	15	3.64963504	NA	NA
DMRcontig45033:65113856	contig45033	65113856	65114000	145	1	1.3496E-06	3	2.06896552	NA	NA
DMRcontig45059:65149507	contig45059	65149507	65149690	184	1	6.6177E-06	8	4.34782609	NA	NA
DMRcontig45108:65214201	contig45108	65214201	65214400	200	1	1.6305E-06	2	1	NA	NA
DMRcontig45141:65259785	contig45141	65259785	65260280	496	1	9.0778E-06	16	3.22580645	NA	NA
DMRcontig45169:65298718	contig45169	65298718	65299100	383	2	8.5235E-09	10	2.61096606	NA	NA
DMRcontig45187:65324283	contig45187	65324283	65324500	218	1	7.3515E-07	5	2.29357798	NA	NA
DMRcontig45362:65556901	contig45362	65556901	65557300	400	1	2.518E-09	6	1.5	NA	NA
DMRcontig45389:65594001	contig45389	65594001	65594100	100	1	3.5584E-06	6	6	NA	NA
DMRcontig45856:66218581	contig45856	66218581	66218818	238	1	5.7561E-07	9	3.78151261	NA	NA
DMRcontig45865:66231233	contig45865	66231233	66231414	182	2	2.2242E-06	5	2.74725275	NA	NA
DMRcontig45871:66239077	contig45871	66239077	66239473	397	2	6.7447E-08	6	1.51133501	NA	NA
DMRcontig45985:66393077	contig45985	66393077	66393370	294	1	4.7875E-06	8	2.72108844	NA	NA
DMRcontig46297:66814082	contig46297	66814082	66814200	119	1	4.4027E-06	1	0.84033613	NA	NA
DMRcontig46341:66871701	contig46341	66871701	66872100	400	3	8.4762E-23	36	9	NA	NA
DMRcontig46387:66931801	contig46387	66931801	66931962	162	1	1.3903E-06	3	1.85185185	NA	NA
DMRcontig46399:66948224	contig46399	66948224	66948500	277	1	6.6348E-06	7	2.52707581	NA	NA
DMRcontig46414:66967301	contig46414	66967301	66967641	341	2	5.2546E-08	8	2.34604106	NA	NA
DMRcontig46432:66990539	contig46432	66990539	66990700	162	2	1.4968E-08	2	1.2345679	NA	NA
DMRcontig46473:67046301	contig46473	67046301	67046600	300	2	1.0792E-07	10	3.33333333	NA	NA
DMRcontig46485:67062101	contig46485	67062101	67062218	118	1	3.7309E-06	4	3.38983051	NA	NA
DMRcontig46507:67091350	contig46507	67091350	67091500	151	1	5.4546E-07	3	1.98675497	NA	NA
DMRcontig46542:67136022	contig46542	67136022	67136761	740	1	1.8923E-08	49	6.62162162	NA	NA
DMRcontig46641:67268189	contig46641	67268189	67268600	412	1	5.7798E-06	12	2.91262136	NA	NA
DMRcontig46688:67328114	contig46688	67328114	67328247	134	1	6.4176E-06	3	2.23880597	NA	NA
DMRcontig46758:67421076	contig46758	67421076	67421157	82	2	5.7374E-06	0	0	NA	NA
DMRcontig46836:67524734	contig46836	67524734	67524895	162	1	8.4602E-06	9	5.55555556	NA	NA
DMRcontig47017:67762257	contig47017	67762257	67762400	144	1	2.1546E-06	1	0.69444444	NA	NA
DMRcontig47019:67764964	contig47019	67764964	67765387	424	1	7.8072E-06	12	2.83018868	NA	NA
DMRcontig47036:67787101	contig47036	67787101	67787300	200	1	4.2433E-07	13	6.5	NA	NA
DMRcontig47139:67920601	contig47139	67920601	67920784	184	1	3.4837E-06	8	4.34782609	NA	NA
DMRcontig47297:68131901	contig47297	68131901	68132200	300	1	5.5885E-06	7	2.33333333	trnar-ccu;epha7	Receptor;Translation
DMRcontig47428:68303040	contig47428	68303040	68303292	253	1	2.2996E-10	8	3.16205534	NA	NA
DMRcontig47455:68338005	contig47455	68338005	68338366	362	1	1.7899E-06	14	3.86740331	NA	NA
DMRcontig47498:68394877	contig47498	68394877	68395024	148	2	5.1129E-08	1	0.67567568	NA	NA
DMRcontig47548:68460301	contig47548	68460301	68461000	700	3	2.2449E-12	30	4.28571429	NA	NA

DMRcontig47578:68501701	contig47578	68501701	68502400	700	2	9.6024E-08	21	3	NA	NA
DMRcontig47730:68701501	contig47730	68701501	68701700	200	2	9.1334E-13	1	0.5	NA	NA
DMRcontig47820:68817411	contig47820	68817411	68817680	270	2	1.4062E-06	7	2.59259259	NA	NA
DMRcontig47900:68923681	contig47900	68923681	68923800	120	1	9.1739E-06	2	1.66666667	NA	NA
DMRcontig47967:69011321	contig47967	69011321	69011600	280	1	6.1906E-06	4	1.42857143	NA	NA
DMRcontig47999:69052267	contig47999	69052267	69052575	309	1	1.1545E-07	8	2.58899676	NA	NA
DMRcontig48081:69159201	contig48081	69159201	69159541	341	1	3.0876E-06	13	3.81231672	NA	NA
DMRcontig48094:69175552	contig48094	69175552	69175700	149	2	4.8303E-09	6	4.02684564	NA	NA
DMRcontig48114:69202401	contig48114	69202401	69202585	185	1	3.6516E-11	7	3.78378378	NA	NA
DMRcontig48131:69224201	contig48131	69224201	69224328	128	1	5.843E-13	0	0	NA	NA
DMRcontig48139:69234544	contig48139	69234544	69234653	110	1	1.0882E-06	2	1.81818182	NA	NA
DMRcontig48252:69380601	contig48252	69380601	69381100	500	1	4.5175E-07	39	7.8	NA	NA
DMRcontig48267:69400301	contig48267	69400301	69400450	150	1	1.1518E-06	2	1.33333333	NA	NA
DMRcontig48272:69406855	contig48272	69406855	69407000	146	1	8.4602E-06	3	2.05479452	NA	NA
DMRcontig48316:69464559	contig48316	69464559	69464800	242	2	5.269E-09	6	2.47933884	NA	NA
DMRcontig48318:69467066	contig48318	69467066	69467500	435	2	3.9025E-10	10	2.29885057	NA	NA
DMRcontig48397:69569601	contig48397	69569601	69569796	196	2	2.1253E-06	7	3.57142857	NA	NA
DMRcontig48428:69610787	contig48428	69610787	69610967	181	1	4.3933E-07	13	7.18232044	NA	NA
DMRcontig48430:69613453	contig48430	69613453	69613700	248	1	3.3053E-08	9	3.62903226	NA	NA
DMRcontig48462:69654452	contig48462	69654452	69654900	449	2	8.6316E-07	13	2.89532294	NA	NA
DMRcontig48480:69677880	contig48480	69677880	69678174	295	2	8.6274E-11	5	1.69491525	NA	NA
DMRcontig48514:69723102	contig48514	69723102	69723438	337	1	1.1285E-06	7	2.07715134	NA	NA
DMRcontig48715:69983515	contig48715	69983515	69983813	299	3	2.5321E-08	11	3.67892977	NA	NA
DMRcontig48905:70227601	contig48905	70227601	70227900	300	1	5.7374E-06	7	2.33333333	NA	NA
DMRcontig48930:70258401	contig48930	70258401	70258600	200	2	6.3221E-07	5	2.5	NA	NA
DMRcontig49020:70371462	contig49020	70371462	70371693	232	3	9.9952E-25	11	4.74137931	LDAH	Metabolism
DMRcontig49131:70514501	contig49131	70514501	70514600	100	1	4.7824E-07	6	6	NA	NA
DMRcontig49350:70792801	contig49350	70792801	70793100	300	1	3.9529E-06	13	4.33333333	NA	NA
DMRcontig49399:70853201	contig49399	70853201	70853400	200	1	6.0235E-06	11	5.5	NA	NA
DMRcontig49430:70893540	contig49430	70893540	70893914	375	1	1.172E-06	8	2.13333333	NA	NA
DMRcontig49857:71428001	contig49857	71428001	71428500	500	1	5.517E-07	17	3.4	NA	NA
DMRcontig49881:71457901	contig49881	71457901	71458050	150	2	1.1176E-09	6	4	NA	NA
DMRcontig50069:71691201	contig50069	71691201	71691700	500	1	4.3289E-07	16	3.2	NA	NA
DMRcontig50071:71694401	contig50071	71694401	71694700	300	2	2.2813E-10	22	7.33333333	NA	NA
DMRcontig50270:71941970	contig50270	71941970	71942345	376	1	3.6015E-08	19	5.05319149	NA	NA
DMRcontig50492:72228101	contig50492	72228101	72228192	92	1	2.7459E-06	1	1.08695652	NA	NA
DMRcontig50498:72235001	contig50498	72235001	72235200	200	1	5.5729E-06	10	5	NA	NA
DMRcontig50569:72325794	contig50569	72325794	72326062	269	3	6.1761E-20	6	2.23048327	NA	NA
DMRcontig50684:72473201	contig50684	72473201	72473538	338	1	5.8398E-06	6	1.77514793	NA	NA
DMRcontig50708:72503401	contig50708	72503401	72503587	187	1	5.2713E-07	6	3.20855615	NA	NA
DMRcontig50737:72541557	contig50737	72541557	72542000	444	1	2.2282E-06	14	3.15315315	NA	NA
DMRcontig50863:72707577	contig50863	72707577	72707900	324	1	1.4357E-07	16	4.9382716	NA	NA
DMRcontig50936:72801631	contig50936	72801631	72801885	255	3	3.6982E-20	12	4.70588235	NA	NA
DMRcontig51151:73075864	contig51151	73075864	73076300	437	2	3.9243E-11	15	3.43249428	NA	NA
DMRcontig51153:73078785	contig51153	73078785	73079100	316	1	1.1766E-06	7	2.21518987	NA	NA
DMRcontig51159:73086501	contig51159	73086501	73086668	168	1	4.9541E-39	2	1.19047619	NA	NA
DMRcontig51282:73236101	contig51282	73236101	73236311	211	3	2.4251E-78	6	2.8436019	NA	NA
DMRcontig51292:73249201	contig51292	73249201	73249800	600	3	1.6892E-07	28	4.66666667	NA	NA
DMRcontig51434:73429101	contig51434	73429101	73429475	375	1	1.5663E-06	11	2.93333333	NA	NA
DMRcontig51469:73476301	contig51469	73476301	73476600	300	3	1.3188E-28	9	3	NA	NA
DMRcontig51522:73544801	contig51522	73544801	73545320	520	1	2.9408E-06	27	5.19230769	NA	NA
DMRcontig51622:73674401	contig51622	73674401	73674800	400	1	1.5774E-06	9	2.25	NA	NA
DMRcontig51734:73817101	contig51734	73817101	73817300	200	2	3.492E-06	7	3.5	NA	NA
